# Bilateral Alignment of Receptive Fields in the Olfactory Cortex

**DOI:** 10.1523/ENEURO.0155-24.2024

**Published:** 2024-11-05

**Authors:** Julien Grimaud, William Dorrell, Siddharth Jayakumar, Cengiz Pehlevan, Venkatesh Murthy

**Affiliations:** ^1^Molecules, Cells, and Organisms Graduate Program, Harvard University, Cambridge, Massachusetts 02138; ^2^Department of Molecular and Cellular Biology and Center for Brain Science, Harvard University, Cambridge, Massachusetts 02138; ^3^Center for Brain Science, Harvard University, Cambridge, Massachusetts 02138; ^4^Cell Engineering Laboratory (CellTechs), SupBiotech, 94800 Villejuif, France; ^5^John A. Paulson School of Engineering and Applied Sciences, Harvard University, Cambridge, Massachusetts 02138; ^6^Kempner Institute for Natural and Artificial Intelligence, Harvard University, Cambridge, Massachusetts 02138

**Keywords:** computational model, correlated receptive fields, Hebbian plasticity, interhemispheric coordination, olfactory cortex

## Abstract

Each olfactory cortical hemisphere receives ipsilateral odor information directly from the olfactory bulb and contralateral information indirectly from the other cortical hemisphere. Since neural projections to the olfactory cortex (OC) are disordered and nontopographic, spatial information cannot be used to align projections from the two sides like in the visual cortex. Therefore, how bilateral information is integrated in individual cortical neurons is unknown. We have found, in mice, that the odor responses of individual neurons to selective stimulation of each of the two nostrils are significantly correlated, such that odor identity decoding optimized with information arriving from one nostril transfers very well to the other side. Nevertheless, these aligned responses are asymmetric enough to allow decoding of stimulus laterality. Computational analysis shows that such matched odor tuning is incompatible with purely random connections but is explained readily by Hebbian plasticity structuring bilateral connectivity. Our data reveal that despite the distributed and fragmented sensory representation in the OC, odor information across the two hemispheres is highly coordinated.

## Significance Statement

Like other sense organs, animals typically have two nostrils, but how odor information from the two sides is combined to build bilateral olfactory representations remains largely unknown. Grimaud et al. found that the responses of neurons in the olfactory cortex in awake mice to odors presented separately to the ipsilateral or contralateral nostril are significantly correlated, beyond chance. Such aligned responses could arise from Hebbian plasticity in interhemispheric connections that relies on common odor experiences across the two nostrils. While responses are correlated, the remaining asymmetries in responses to the two nostrils allowed decoding of stimulus laterality. This study points to unexpected order in an olfactory circuit and prompts future work on how olfactory experience can shape interhemispheric information integration.

## Introduction

In vision and audition, information from paired sensors (eyes or ears) is combined in the brain to perform specific computations that are behaviorally relevant—for example, estimating distance to or the direction of the source of the stimuli (Cumming and DeAngelis, 2001; Grothe and Pecka, 2014). The existence of two sensors for olfaction has prompted hypotheses about their function (Mainland et al., 2002; Porter et al., 2005; Rajan et al., 2006; Catania, 2013; Rabell et al., 2017). Information from the two nostrils can be differentiated, or integrated, depending on the goal of such computation for the animal—for example, odor source localization when smooth gradients exist (Catania, 2013; Gire et al., 2016; Rabell et al., 2017; Álvarez-Salvado et al., 2018; Baker et al., 2018) or identification of relevant smells independent of the odor source location or nostril stimulated (Bojsen-Moller and Fahrenkrug, 1971; Kucharski and Hall, 1987, 1988; Eccles, 2000; Mainland et al., 2002; Kikuta et al., 2008; Dalal et al., 2020). These computations will dictate the nature of the interhemispheric communication and the mechanisms of integration of bilateral olfactory cues.

**Figure 1.**
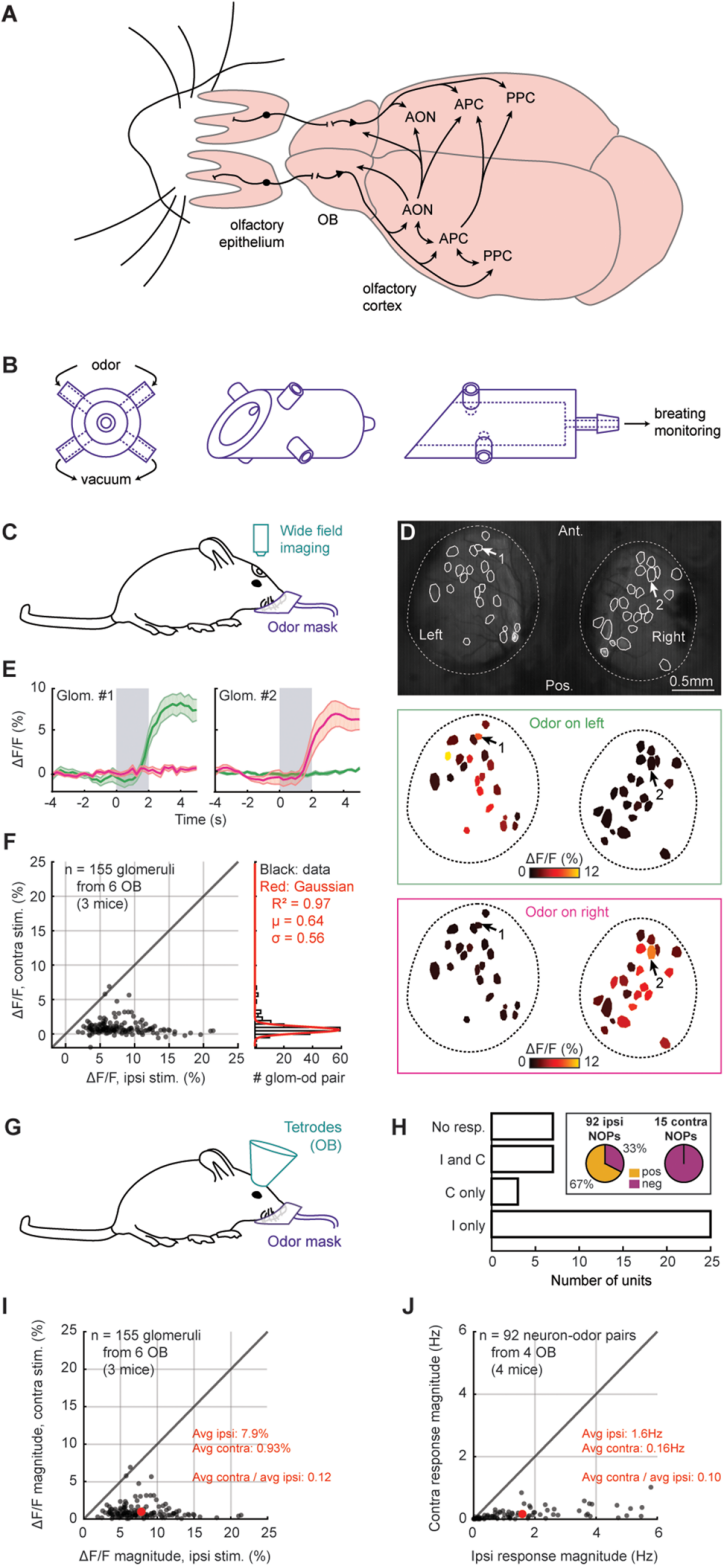
A new method for unilateral odor delivery. ***A***, Projection between the different olfactory regions. Only the OC (in particular, the AON and APC) sends direct contralateral projections. ***B***, Diagram of the odor mask. From left to right, Front, rotated, and side view. ***C***, Calcium imaging setup. ***D***, Exemplar glomerular activity. Example, All glomeruli from OB1, isopropyl tiglate. Arrows, See below. ***E***, Exemplar glomerular activity over time. Gray, Odor on (here, isopropyl tiglate). Green, Odor on the left nostril. Pink, Odor on the right nostril. Average ± SEM. Glomeruli 1 and 2 correspond to Arrows 1 and 2 in panel ***D***. ***F***, Glomerular responses. Each dot is the response of one glomerulus to one odor (glom–od pair). Histogram, Contralateral responses. Red, Gaussian fit. ***G***, Tetrode recording setup. ***H***, Unilateral response profile of M/T cells. NOP, Neuron–odor pair. ***I***, Magnitude of glomerular response. Red, Average for each side. This panel presents the magnitude (i.e., the absolute value) of the responses displayed in panel ***F***. ***J***, Magnitude of M/T cell response. Only the ipsilaterally responding NOPs are shown. Red, Average for each side. See also Extended Data Figure 1-1 and Extended Data Tables 1-1 and 1-2.

Odors are detected by olfactory sensory neurons in the nose, which project to the ipsilateral olfactory bulb (OB). The OB sends information to multiple ipsilateral brain regions, including the anterior olfactory nucleus (AON) and the anterior (APC) and posterior piriform cortices (Mori et al., 1999; Wilson and Sullivan, 2011; Giessel and Datta, 2014; Nagayama et al., 2014; Fig. 1*A*) In many species, including rodents, the two nares are separated by a septum that minimizes transfer of inhaled odors between nostrils (Wilson and Sullivan, 1999; Eccles, 2000; Kikuta et al., 2008). Therefore, information transfer across hemispheres is largely neural and originates in the cortex (Haberly and Price, 1978; Scott et al., 1980; Kikuta et al., 2008; Wilson and Sullivan, 2011; Boyd et al., 2012; Bekkers and Suzuki, 2013; Fig. 1*A*).

10.1523/ENEURO.0155-24.2024.f1-1 Figure 1-1 **A New Method for Unilateral Odor Delivery.** Related to Figure 1. (A) Odorants used in this study. (B) Control of the symmetry of the olfactometer. A PID measured the changes of odor concentration over time at the end of the right or the left end of the facial mask. Each measurement was repeated three times; therefore, each graph shows six over-imposed traces, three with the PID on the right, three on the left. Note the similarity between the traces obtained on the left and right of the mask, and the absence of odor detected on the contralateral side. Grey: odor presentation (here: isopropyl tiglate). All 15 odors were tested and showed similar results. (C) Diagram of the setup used for assessing the reliability of the face mask for respiration monitoring. The breathing of awake mice was monitored simultaneously with the mask and a pressure sensor connected to a chronic intranasal cannula (n = 3 animals, 15  min of continuous recording per mouse). (D) Exemplar breathing traces, simultaneously recorded from the intranasal cannula (light blue) and the face mask (purple). Note the synchrony of both signals and the apparent absence of phase shift. (E) Histogram of instantaneous breathing rates, calculated with the exhalation (top panel) or inhalation (bottom panel) onsets. The data shown here comes from the full monitoring session of one exemplar mouse. The distributions obtained from the intranasal cannula (light bars) or the face mask (dark bars) are almost identical. (F) Delay from cannula to mask, calculated with the exhalation (green) or inhalation (orange) onsets. The data from the three mice were combined here. Values: median ± SD. The phase shift between the intranasal cannula and the face mask is small and reliable. (G) Example of tetrode recording. The four electrodes belong to the same tetrode. Grey: odor delivery. Green: unsorted electrode traces. Blue and red: two single units. Purple: sniffing acquired simultaneously (up: inhalation). These exemplar traces were recorded in mouse APC1. (H) Example of single unit clustering. The red and blue units are the same as in (G). (I) Example of clustered single units. The red and blue units are the same as in (G). Lighter traces: all the peaks from the recording session were over-imposed. Darker traces: average peaks. (J) Spike count versus inter-spike interval (ISI) for the two units shown in (G) (blue and red). Note the presence of a refractory period. Download Figure 1-1, TIF file.

**Figure 2.**
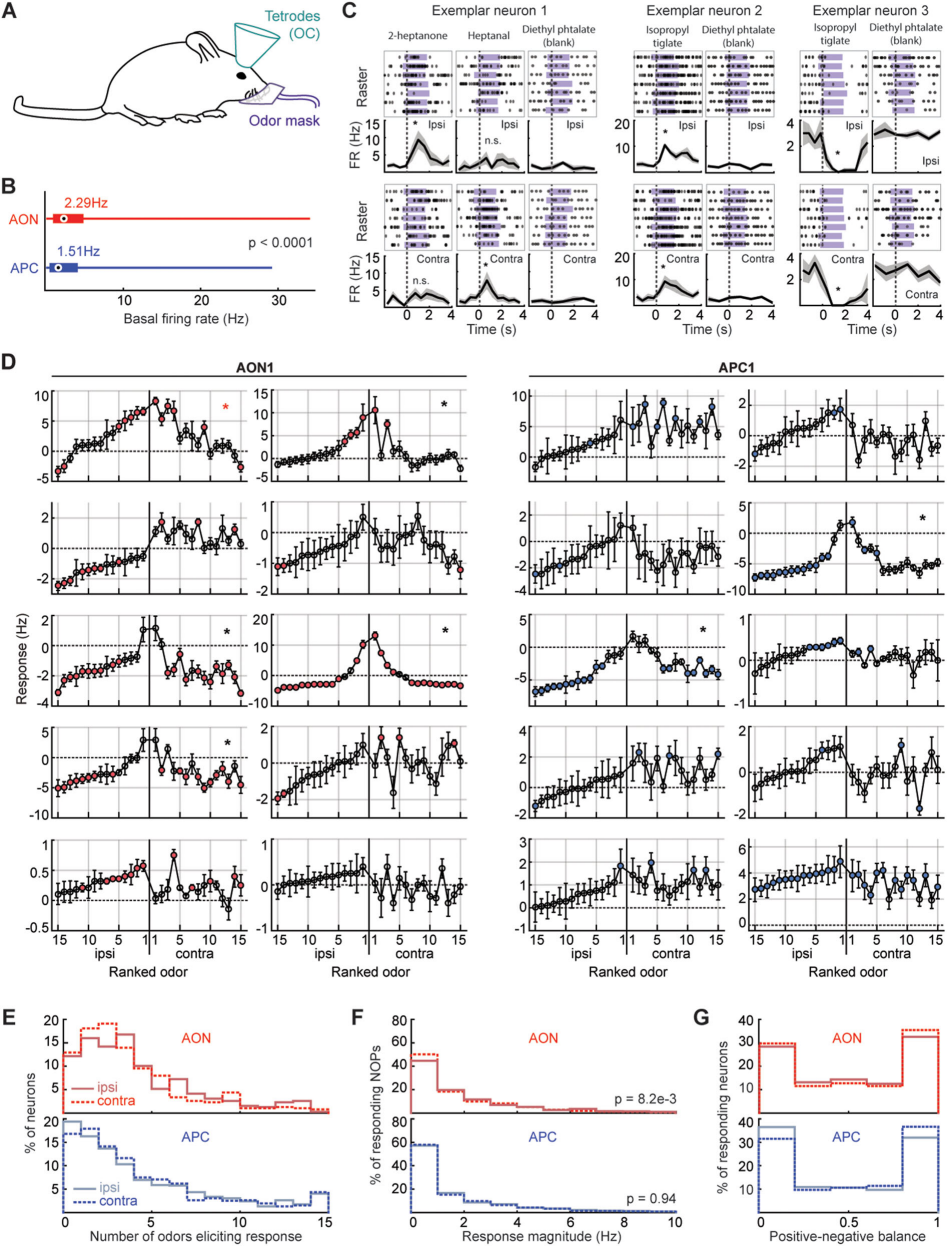
Odor receptive fields of neurons in the OC. ***A***, Experimental setup. ***B***, Basal activity per region. Circle and value next to it, Median. Thick bar, Quartiles. Thin bar, Range of values. ***C***, Exemplar odor responses over time. Purple, Odor delivery. Responses were aligned to the first sniff following the odor onset (dotted line). PSTH, average ± SEM. Examples taken from mouse APC1 (see Extended Data Table 1-1 for a detailed list of all mice recorded for this study). For all tetrode recordings reported in this study, changes of the firing rate are calculated by subtracting the firing rate during the 4 s preceding odor delivery to the firing rate during the 2 s of odor delivery. Odor responses are averaged over all seven trials and blank subtracted. See Materials and Methods for details. ***D***, Odor responses, illustration. Neurons randomly picked from mice AON1 (left) and APC1 (right). For each neuron, odors were ranked based on the response magnitude (i.e., the absolute value of their response) to ipsilateral presentations, which are shown in the left half of each plot. The contralateral responses are then shown with the same rank order of odors for each neuron. Each circle gives the average response ± SEM across the seven trials, and filled circles represent significant responses (circles filled in red for AON recordings, blue for APC). Asterisk, The ipsi- and contralateral odor responses are significantly correlated. See Figure 3*A* for details. ***E***, Distribution of the number of odors eliciting a significant response in a given neuron, per region and side. ***F***, Distribution of significant response magnitudes for all neurons and odors, per region and side. ***G***, Positive–negative balance, per region and side. 0 = all negative significant responses; 1 = all positive. See also Extended Data Figure 2-1.

10.1523/ENEURO.0155-24.2024.f1-2 Figure 1-2 **Instructions to Build an Odor Delivery Mask.** Related to Figure 1. Below we explain how to build a mask, including all necessary measurements and materials. (A) Cut a piece of PVC tubing (outer diameter: 18  mm; inner diameter: 13  mm) as indicated on the diagram. PVC allows for a gentle yet firm seal around the mouse's face. (B) Using injection needles of gradually increasing diameters, puncture the piece of tubing at the positions indicated by the red marks on the diagram. When puncturing, insert the needle tangentially to the piece of tubing. Each hole should be less than 0.09in (2.2  mm) in diameter, to ensure that the tube fitting fits snugly. (C) Insert the nylon tube fittings as indicated on the diagram. For the odors and vacuums, use tube fittings ref. 51525K211 (McMaster-Carr, USA). For the breathing, use a tube fitting ref. 5463K439 (McMaster-Carr, USA), and cover the threads with teflon tape before insertion. Secure all tube fittings in position by applying a few drops of epoxy glue around each of them, on the outside of the mask. Let the mask rest for at least a week in a well-ventilated area, so as to eliminate any remaining epoxy fumes. Download Figure 1-2, TIF file.

**Figure 3.**
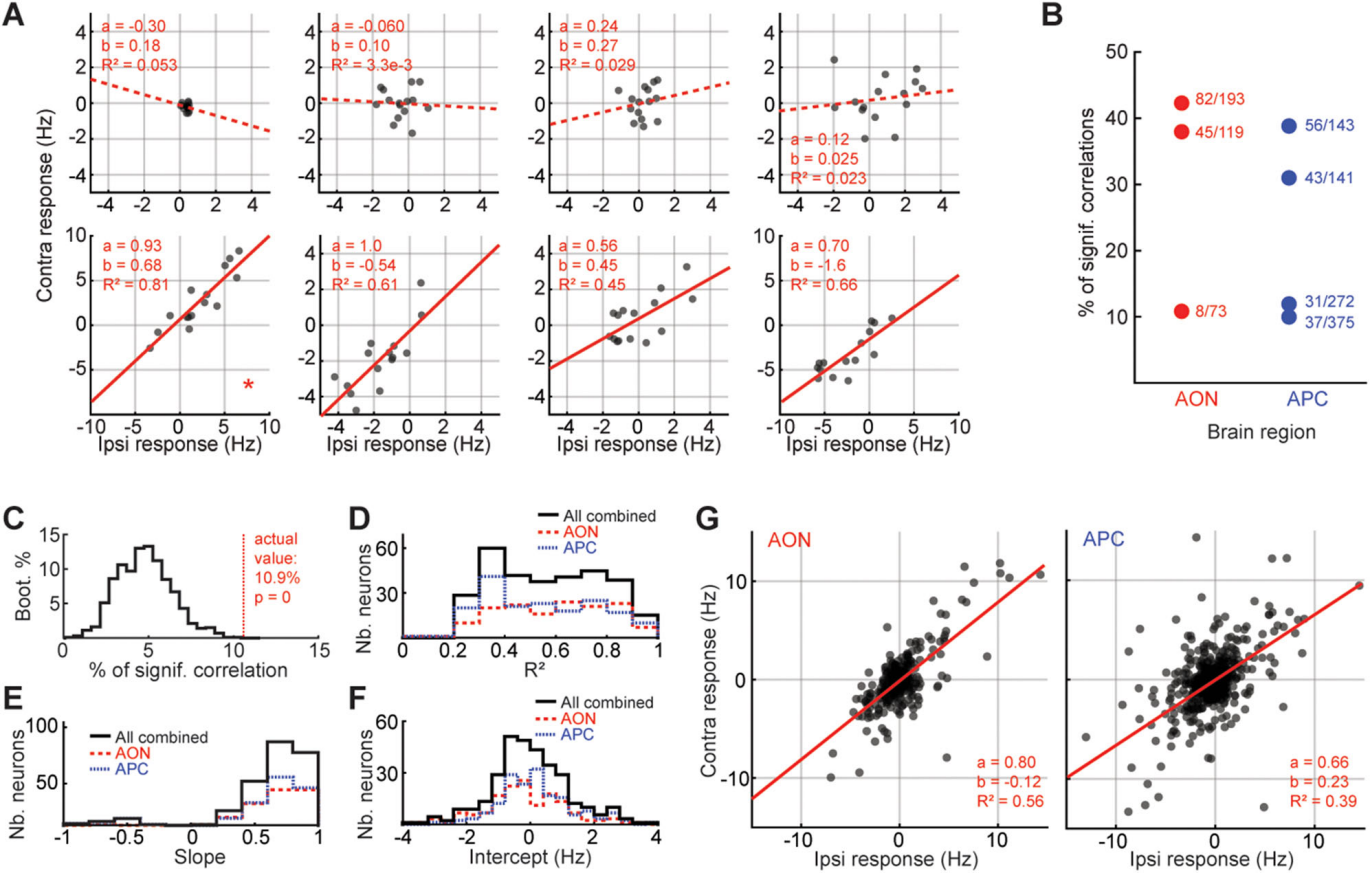
Side-invariant odor responses in the OC. For panels ***A*** and ***G***, red line, linear regression (contra = a × ipsi + b). Solid lines show significant regressions (*F* test, *p* < 5%). ***A***, Exemplar ipsi- versus contralateral responses per neuron. Examples picked from AON1. Each dot is a different odor. The neurons in the bottom panels are bilaterally correlated. Asterisk, Same neuron as in Figure 2*D*, left panel, red asterisk. ***B***, The percentage of BCNs per mouse. Each dot is a mouse. Fractions next to each dot, Number of BCNs / total number of neurons. Full circle, The fraction is significantly higher than chance. ***C***, Significance of the percentages of BCNs. Here we show the distribution of chance percentages for AON1, obtained with a bootstrapping procedure. ***D–F***, Coefficients of determination, slopes, and intercepts of the BCNs in the AON and APC (Nb, number). ***G***, Ipsi- versus contralateral responses per region for Odor 1 (isopropyl tiglate). Each dot represents one neuron. For the equivalent graphs across all odors, see Extended Data Figure 3-2. See also Extended Data Figures 3-1–3-3.

10.1523/ENEURO.0155-24.2024.f1-3 Figure 1-3 Glomerular Activity at Various Odor Concentrations. Related to Figure 1. (A) Activity of one exemplary glomerulus to unilateral presentations of ethyl tiglate at various concentrations. Purple: odor delivery. Each trace is one odor delivery (3 repeats per side, per concentration). (B) Glomerular activity. Each line represents the activity of one glomerulus to one odor, averaged over three repeats on each side (N = 52 glomeruli from 3 mice). Four odors were tested in total (ethyl tiglate, isopropyl tiglate, hexanal, allyl butyrate), each odor was tested at three different concentrations (5%, 0.5%, and 0.05% volume/volume in diethyl phthalate), with three repeats for each condition. All recordings were performed on awake mice. Responses are shown as “delta F” (mean fluorescence during the 2  s of odor presentation minus mean fluorescence over the two seconds before) rather than “delta F over F0” as some glomeruli showed no detectable activity before odor presentations. The distributions of glomerular responses are also shown as box plots (center dot: median; thick bar: quartiles; thin bar: 95% confidence interval). (C) Left: ipsi- versus contralateral responses at 5% concentration. Right: ipsilateral responses at 5% concentration versus ipsilateral responses at 0.5% concentration. Dotted line: linear regression (response on the y-axis = slope x response on the x-axis). The regression slope is given on each graph. Download Figure 1-3, TIF file.

**Figure 4.**
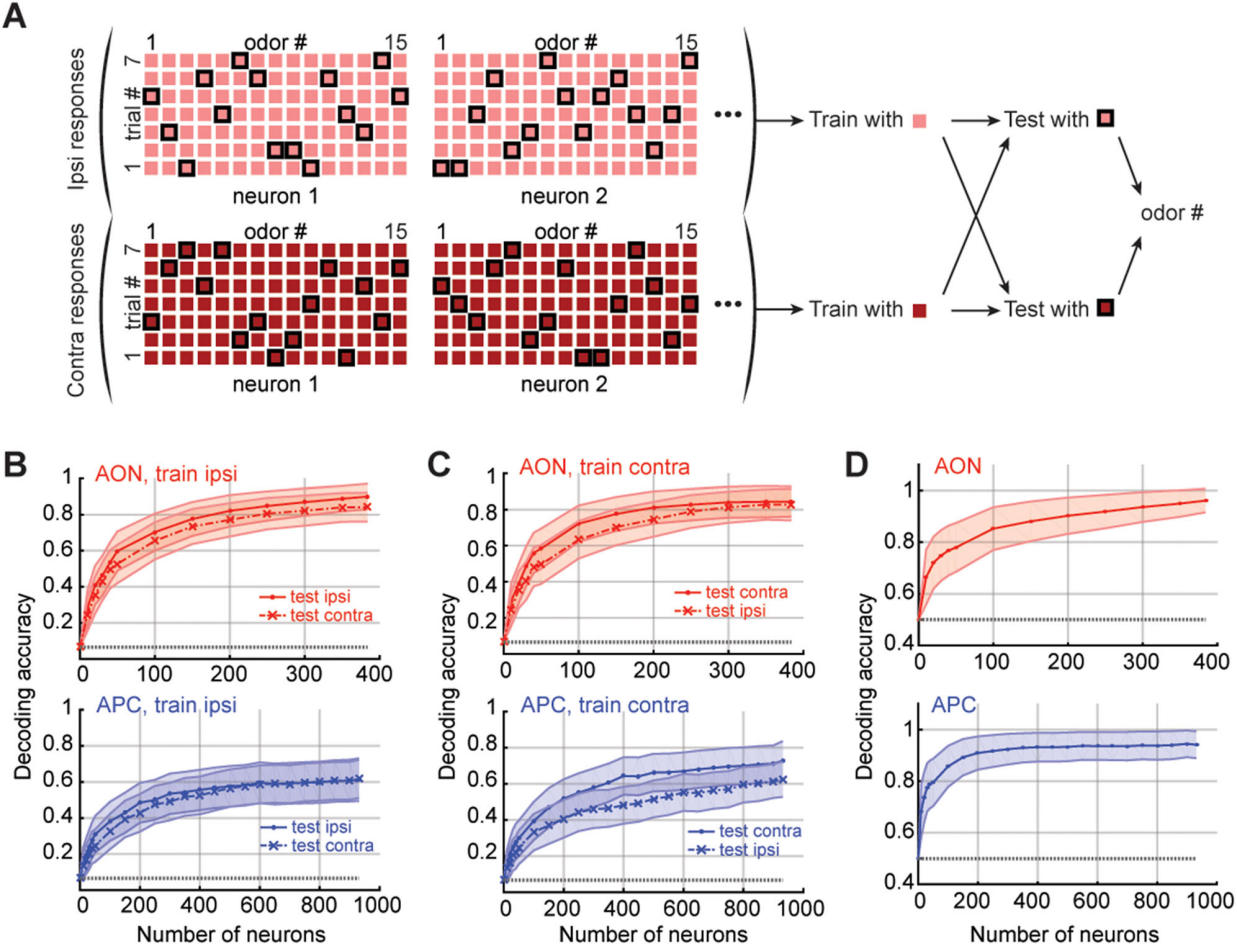
Decoding odor identity and laterality. ***A***, Diagram of the odor decoding process. ***B***,***C***, Odor identity decoding per region. Mean ± SEM over 200 repetitions of the decoding process. For ***B***, the decoder was trained with data from ipsilateral presentations. For ***C***, the decoder was trained on contralateral odor responses. ***D***, Side identity decoding per region. Mean ± SEM over 200 repetitions of the decoding process. For ***B*–*D***, gray dotted line, Chance. See also Extended Data Figures 4-1 and 3-3.

10.1523/ENEURO.0155-24.2024.f1-4 Figure 1-4 Glomerular Activity After Naris Occlusion. Related to Figure 1. (A) Activity of one exemplary glomerulus to unilateral presentations of ethyl tiglate, after the mouse's contralateral nostril has been occluded. Purple: odor delivery. Each trace is one odor delivery (3 repeats per side). (B) Glomerular activity after naris occlusion. Each line represents the activity of one glomerulus to one odor, averaged over three repeats on each side (N = 42 glomeruli from 4 imaging sessions - two mice were imaged once with their right nostril occluded, while a third mouse was imaged on two separate sessions, once with its right naris occluded and once with its left naris). While we blindly looked for glomeruli on both OB, only 4 glomeruli out of 42 were located on the OB ipsilateral to the occlusion. Four odors were tested in total, three repeats each (ethyl tiglate, isopropyl tiglate, hexanal, allyl butyrate; 5% volume/volume dilution in diethyl phtalate). All recordings were performed on awake mice, the day following the naris stitching. Responses are shown as “delta F” (mean fluorescence during the 2  s of odor presentation minus mean fluorescence over the two seconds before) rather than “delta F over F0” as some glomeruli showed no detectable activity before odor presentations. The distribution of glomerular responses to odors delivered to the not occluded and occluded nostrils are also shown as box plots (center dot: median; thick bar: quartiles; thin bar: 95% confidence interval). The median response to the non occluded nostril is 20.7 times greater than to the occluded nostril. (C) Same as (B), except we show the glomeruli from the OB contra- and ipsilateral to the naris occlusion separately. On the OB contralateral to the occlusion, the median response to the non occluded nostril is 15.4 times greater than to the occluded nostril. Download Figure 1-4, TIF file.

10.1523/ENEURO.0155-24.2024.t1-1 Table 1-1 **Mice Recorded in this Study**. All tetrode recordings were performed in awake, head-restrained mice. Download Table 1-1, DOCX file.

10.1523/ENEURO.0155-24.2024.t1-2 Table 1-2 **Statistical Tests Performed in this Study**. The statistical tests showing significant differences are in red (critical value: 5%). For post-hoc tests, the p-values reported in the table have been corrected for multiple testing problem (Bonferroni method). Download Table 1-2, DOCX file.

Bilateral cortical interactions have been investigated in other senses including vision, where the relevant brain areas have ordered representations of the world using topographic maps, which allow neural matching through precise point-to-point mapping (Luo and Flanagan, 2007; Cang and Feldheim, 2013; Gu and Cang, 2016). However, the olfactory cortex (OC) contains no recognizable topography (Illig and Haberly, 2003; Stettler and Axel, 2009; Ghosh et al., 2011; Miyamichi et al., 2011; Sosulski et al., 2011; Wilson and Sullivan, 2011; Bekkers and Suzuki, 2013; Pashkovski et al., 2020), making point-to-point matching of bilateral information through continuous maps implausible. It is possible that odor representations in the two primary OC hemispheres are essentially independent, and downstream areas align them for coherent perception, as implicitly proposed by Schaffer et al. (2018).

Framing hypotheses about bilateral olfactory processing requires basic information about how single cortical neurons respond to the same odor presented to the two nostrils. Such information is fundamental for uncovering the computations performed, just as details about binocular matching and disparity in cortical responses were for vision (Blake and Wilson, 2011). Individual neurons could respond to ipsi- and contralateral odor stimuli in a congruent or disparate manner. There is some evidence for responses in olfactory cortical areas to contralateral odor stimulation, but there is little information on the alignment of ipsi- and contralateral responses (Wilson, 1997; Kikuta et al., 2008, 2010). Recent evidence suggests that mitral cells in the OB may have matched responses through organized feedback projections from the AON pars externa (Yan et al., 2008; Grobman et al., 2018). A cortical neuron may then be able to inherit the matched response from mitral cells (Dalal et al., 2020), but a direct evaluation of cortical matching is lacking. As this work was being prepared, Dikeçligil et al. (2023) reported that the piriform cortex responses to stimuli in the contralateral nostril were time delayed but similar to those evoked from ipsilateral stimulation, suggesting that OC neurons may inherit such matched responses.

To investigate how the OC combines information from the two nostrils, we recorded odor-evoked spiking activity of individual neurons in the OC of awake mice. We observed neuron responses to selective odor stimulation through the contralateral nostril in all cortical regions recorded. A significant fraction of OC neurons showed a strong match of ipsi- and contralateral response profiles. Population activity in OC could be used to accurately decode odor identity, as well as side identity. A computational model of the olfactory system suggested that the bilateral overlap we observed in mice is only possible with nonrandom bilateral projections. Our results provide fundamental and novel insight that defines how ipsi- and contralateral information streams converge and ultimately enhance the processing capabilities of the brain.

## Materials and Methods

### Experimental model and subject details

All tetrode recordings were obtained from male C57Bl/6J mice. In addition, we used male OMP-GCaMP3 mice (Isogai et al., 2011; Fig. 1), as well as male and female OMP-Cre crossed to floxed jGCaMP8s mice (The Jackson Laboratory, strain 037719; Extended Data Figs. 1-3, 1-4) for the glomerular imaging experiments. All the mice were 3–8 months old at the time of the experiments. Mice were singly housed after chronic tetrode implantation. A summary of the mice used for tetrode recording can be found in Extended Data Table 1-1. All experiments were performed in accordance with the guidelines set by the National Institutes of Health and approved by the Institutional Animal Care and Use Committee at Harvard University.

### Bilateral odor stimulation

The odor panel was composed of 15 monomolecular odors, purchased from Millipore Sigma: isopropyl tiglate, ethyl tiglate, propyl acetate, isoamyl acetate, ethyl valerate, hexanal, heptanal, allyl butyrate, citronellal, hexyl tiglate, 4-allyl anisole, isobutyl propionate, 2-heptanone, ethyl propionate, and eucalyptol (Extended Data Fig. 1-1*A*). The odors were diluted in diethyl phthalate, also purchased from Millipore Sigma (final odor dilution, 5% volume/volume). In addition to these 15 odors, we also tested the response to diethyl phthalate alone, referred to as the “blank” trials (see below, Neuronal recording). The olfactometer was controlled by a custom LabVIEW script (National Instruments).

Odors were delivered through a custom-built facial mask (Fig. 1*B*; Extended Data Fig. 1-2). The mask was made of a large piece of tubing surrounding the mouse's muzzle. On each side of the mask, close to each nostril, a piece of tubing delivered odors at a small flow rate (50 SCCM). Also on the mask, right below each odor delivery tube, a vacuum (50 SCCM, similar to the odor line) ensured proper air cleaning. We used a flowmeter to inspect and regulate both the odor and vacuum lines daily. In addition, before each recording session, we ensured that the odor and vacuum lines were at a similar flow rate by visually inspecting that the breathing signal, which we monitored from a dedicated airflow sensor, was centered around zero, once the mask was pressed against the mouse's head (see below, Breathing monitoring). We confirmed that the mask could deliver similar amounts of odor on each side of the mask with a photoionization detector (PID) (miniPID 200B, Aurora Scientific; Extended Data Fig. 1-1B). We confirmed the absence of significant contralateral stimulations through calcium imaging at the surface of the OB and mitral/tufted (M/T) cell recordings (Fig. 1).

Odors were delivered for 2 s for each trial. To avoid odor habituation, a period of 15 s separated consecutive trials (i.e., 15 s from the end of a trial to the beginning of the next trial). Each odor was delivered in two configurations: ipsilateral and contralateral to the implantation. Each odor delivery was repeated seven times. Therefore, each recording session contained (15odors+1blanktrial)×2sides×7repetitions=224 odor presentations. The order of odor presentations was randomly shuffled for each recording session.

### Breathing monitoring

The face mask was connected to an airflow sensor (Honeywell AWM3300V) to monitor the animal's respiration during the experiments (Fig. 1*B*) and later align neuronal activity with the sniffs (Fig. 2*C*; Bolding and Franks, 2017). The accuracy of the face mask in monitoring respiration was tested against intranasal pressure transients (Extended Data Fig. 1-1*C*–*F*) as described previously (Reisert et al., 2014).

10.1523/ENEURO.0155-24.2024.f2-1 Figure 2-1 Post-Mortem Confirmation of Tetrode Placement in the Olfactory Cortex. Related to Figure 2. Sagittal sections of two exemplar animals, implanted in the AON (left) and APC (right). The round shape on the ventral end of the tetrode scar in the left panel was caused by post-mortem electrolesion. For animals recorded in the APC, the tetrodes went all the way through the brain, until they reached the ventral part of the skull. As a consequence, it was not possible to save the most anterior part of the section in the right panel. A: anterior. P: posterior. D: dorsal. V: ventral. CC: corpus callosum. LV: lateral ventricle. LOT: lateral olfactory tract. Scale bar: 0.25  mm (left) or 1  mm (center, right). DAPI staining. We did not trace the border between striatum and amygdala as we could not confidently determine it. Download Figure 2-1, TIF file.

### Chronic tetrode implantation

Mice were anesthetized with an intraperitoneal injection of ketamine and xylazine (100 mg/kg and 10 mg/kg, respectively) and then placed in a stereotaxic apparatus. After the skull was cleaned and gently scratched, a custom-made titanium head bar was glued to it. A small craniotomy was performed above the implantation site, before eight custom-built tetrodes (Chang et al., 2013) were lowered together into the brain (coordinates for the AON pars principalis, anteroposterior 3 mm, mediolateral 1.5 mm, dorsoventral 2.3 mm; APC, anteroposterior 1.6 mm, mediolateral −2.8 mm, dorsoventral 3.4 mm; OB, anteroposterior 1.2 mm, mediolateral 1.1 mm, dorsoventral 0.3 mm; all anteroposterior and mediolateral coordinates are given relative to the bregma, except for the OB, for which they are given relative to the junction of the inferior cerebral vein and superior sagittal sinus; all dorsoventral coordinates are given relative to the brain surface). A reference electrode was implanted on the occipital crest. All the mice were implanted in the right hemisphere, except mouse APC2, which was implanted in the left hemisphere. We did not find any difference between APC2 and the other mice. The whole system was stabilized with dental cement. Mice were given a week of recovery before any new manipulation.

### Neuronal recording

A week after chronic tetrode implantation, mice were progressively habituated to stay calm while head-fixed on the recording setup. The habituation process typically took a week. Brain activity was then recorded once a day, always at the same period of the day, while delivering odors to the ipsilateral or contralateral nostril. The mice were awake, head-restrained, and *ad libitum* breathing during all the recordings.

Since the tetrode headstage allowed for the tetrodes to be finely adjusted up or down, the tetrodes were slightly lowered in the brain after each recording session (∼40 µm deep), ensuring that different neurons were recorded each day.

Electrical activity was amplified, filtered (0.3–6 kHz), and digitized at 30 kHz (Intan Technologies, RHD2132, connected to an Open Ephys board). Single units were sorted off-line manually using the MClust script (Schmitzer-Torbert et al., 2005) written for MATLAB (MathWorks). Units with ∼1% of their interspike intervals below 2 ms refractory period were discarded. Units displaying large changes of amplitude or waveform during the recording were also discarded (Rey et al., 2015; Extended Data Fig. 1-1*G*–*I*). The position of the tetrodes in the brain was confirmed postmortem through electrolesion (200 μA for 4 s per channel; Extended Data Fig. 2-1).

The average response *R* to an odor *o* (in Hz) over the seven trials *t* was calculated as follows:

$$R=\overset{7}{\underset{t=1}{\sum }}\;({\mathrm{Dur}}_{o}^{t}-{\mathrm{Bef}}_{o}^{t})/7-\overset{7}{\underset{t=1}{\sum }}\;({\mathrm{Dur}}_{b}^{t}-{\mathrm{Bef}}_{b}^{t})/7,$$

where, for each trial *t*, ${\mathrm{Dur}}_{o}^{t}$ (resp. ${\mathrm{Dur}}_{b}^{t}$) is the firing rate during the odor (resp. “blank”) delivery, while ${\mathrm{Bef}}_{o}^{t}$ (resp. ${\mathrm{Bef}}_{b}^{t}$) is the firing rate before the odor (resp. “blank”) delivery.

${\mathrm{Dur}}_{o}^{t}$ (resp. ${\mathrm{Dur}}_{b}^{t}$) was calculated for the first 2 s following the first sniff after the odor (resp. “blank”) onset. ${\mathrm{Bef}}_{o}^{t}$ (resp. ${\mathrm{Bef}}_{b}^{t}$) was calculated for the 4 s preceding the odor (resp. “blank”) onset.

To determine the significance of a response to an odor *o* over the seven trials *t*, we compared the activity evoked by the odor $\left({\mathrm{Dur}}_{o}^{t}-{\mathrm{Bef}}_{o}^{t}\right)$ with the “blank” activity $\left({\mathrm{Dur}}_{b}^{t}-{\mathrm{Bef}}_{b}^{t}\right)$ (Wilcoxon signed-rank test, critical value set to 5%; Bolding and Franks, 2017).

Finally, we investigated whether a neuron that responds to a particular odor with an increase in the firing rate tends to exhibit increases in firing rates for any other odor it responds to and, similarly, whether a neuron having an inhibitory response to an odor is also inhibited by any other odor it responds to. To do so, we calculated a “positive–negative balance” metric. For each neuron responding significantly to at least one odor, the positive–negative balance corresponds to the fraction of significant responses that are positive. For example, if a neuron has a positive–negative balance of 0.5, then we know that, among the odors eliciting a significant response, half of them elicit an increase of activity, while the other half elicit a decrease. Similarly, a balance of 0 means that all the significant odor responses are negative, and a balance of 1 indicates that all the significant responses are positive.

### Calcium imaging

Mice were anesthetized with an intraperitoneal injection of ketamine/xylazine mixture (100 mg/kg and 10  mg/kg, respectively). After the skull was cleaned and gently scratched, a custom-made titanium head bar was glued to it. A large craniotomy was performed above the two OBs and covered with 1% low melting point agarose (Thermo Fisher Scientific). While the mouse was still anesthetized, calcium activity was imaged above the craniotomies with a custom-made epifluorescence microscope (10× objective, Olympus, NA 0.3; blue excitation LED, Thorlabs; emission filter, 500–550 nm, Thorlabs; CMOS camera, The Imaging Source) at 4 frames/s while presenting odors with the custom-built facial mask (Fig. 1*A*,*B*).

Alternatively, we also implanted glass coverslips over the craniotomy using Vetbond (3M). Briefly, two 3 mm No. 1 glass coverslips (Warner Instruments) were glued together with an optical adhesive (Norland Optical Adhesive 61). Mice postsurgery were treated with carprofen (6 mg/kg) and extended-release buprenorphine (3.25 mg/kg). Animals were allowed to recover for at least five days prior to imaging. For data from Extended Data Figures 1-3 and 1-4, images were acquired at 10 frames/s.

The acquired sequences of images were converted to Δ*F*/*F* images (change in fluorescence over resting fluorescence). Then the maximal response at each pixel was calculated for each of the 15 odors, for each stimulus configuration (right or left nostrils), and these maximum response maps were used to manually identify glomeruli for further analysis. Finally, for each odor, for each stimulus configuration, the response at each glomerulus was quantified by integrating the Δ*F*/*F* signal 4 s after the odor onset (which corresponds to the 2 s of odor presentation and the 2 s immediately following).

### Nostril occlusion

We occluded the nostril of OMP-Cre; jGCaMP8s animals using 7–0 gauge MicroSutures (S-N718SP13). Mice were given lidocaine on the nostril and were anesthetized using an intraperitoneal injection of ketamine/xylazine mixture (100 mg/kg and 10 mg/kg, respectively). The suture was pulled through the upper lip of the nostril, and the nostril was closed completely. The closure of the nostril was confirmed using a drop of water placed on the nostril. Mice were administered extended-release buprenorphine (3.25 mg/kg). The occlusion was tested using the same method before and after the imaging sessions.

### Decoding task

#### Neuronal decoding

For each neuron we created a feature vector by calculating the summed spike count in a 2 s window after the mouse first sniffs the odor and subtracting from it the same sum from the “blank” odor averaged across seven trials.

Odor identity decoding ability was assessed using linear classifiers and leave-out cross–validation on a pseudopopulation made up of all neurons recorded in a particular cortical area—AON or APC (Bolding and Franks, 2017). At each instance, we chose a particular dataset (either ipsilateral or contralateral odor presentation) and formed from it a training set by randomly removing one trial per odor. We calculated mean responses for each odor by averaging across the training set. We then chose our test set in two related ways. If we were testing on the same side as we trained (e.g., train ipsilaterally, test ipsilaterally), then the 15 trials (one per odor) outside the training set were used as the test set. If we were testing on the opposite side (e.g., train ipsilaterally, test contralaterally), then one trial per odor from the opposite side was randomly chosen to form the test set. Classification accuracy was measured by assigning each test trial to the closest Euclidean mean odor response and calculating the percentage of trials correctly assigned (Bolding and Franks, 2017). This process was repeated 200 times using different random test and train trial choices.

To examine the effect of changing number of neurons on classification accuracy, we randomly selected neurons from the pseudopopulation and performed classification using only the selected cells. This process was repeated 200 times. We then reported the average accuracy and standard deviation (SD) over those 200 selections of neurons and, for each set of neurons, 200 sets of train/test trials in Figure 4 and Extended Data Figure 4-1.

A very similar process was employed for side decoding. One trial per side was removed; the remaining trials were averaged to form a mean side response for both ipsilateral and contralateral presentations. The removed trials were then classified to the nearer mean side response, and accuracy was the percentage correctly assigned. This was repeated 200 times for different random removed trial choices.

#### Comparisons to the theoretical model

Our model predicts the trial-averaged neural responses. To make comparisons to the neuronal data, we designed a new classification task. For simulations and trial-averaged neuronal data, we performed odor decoding as follows: we assigned each of the 15 odor centroids from one side (contralateral or ipsilateral) to their closest neighbor on the other side. This classification was correct if an odor from one side was classified to the same odor on the other side. The accuracy was then the proportion of odors correctly assigned. Again, average accuracy and SD over 200 randomly chosen neurons were reported in Figure 4 and Extended Data Figure 4-1. One exception to the procedure above was Extended Data Figure 6-1*D*, right panel, where we classified centroids to their nearest angular neighbor.

For side classification comparisons, the two centroids corresponding to a given odor presented on both sides were removed. From the remaining 28 centroids, two points representing the ipsilateral and contralateral cortex were made by averaging over all remaining centroids from a particular side. The two points we removed were then classified as ipsilateral or contralateral depending on which mean they were nearest to. This procedure was repeated 15 times, removing a different odor each time. We reported the average accuracy and SD over 200 randomly chosen pseudopopulations in Figure 4 and Extended Data Figure 4-1.

### Computational modeling

We built a feed-forward neural network model to explore the implications of a random or structured connection between the OC in each hemisphere. We effectively modeled one cortical region in each hemisphere, based on the AON.

To begin, we considered the cortical response to odors presented ipsilaterally; later we will link these to explore interhemispheric effects. Each cortical neuron's activity to an ipsilateral odor o is a weighted sum of glomerular activities passed through an activation function as follows:

$$y_{o,i}=f(\Sigma_{\;j=1}^{N_{x}}J_{\mathrm{ij}}x_{o,j})=f(h_{o,i}),$$

where ***J*** is the glomerular to cortical connectivity matrix; $x_{o,j}\;\mathrm{and}\;y_{o,i}$ are the firing rates of glomerulus j and cortical neuron i, respectively, to odor o; $h_{o,i}$ is the input to cortical neuron i; *j* runs from 1 to $N_{x}$ (the total number of glomeruli); and *f* is the activation function.

The zero point of these neurons is set to be the basal firing rate plus response to a blank odor. Therefore, we modeled only odor-related perturbations from the blank and base to match all comparisons to the blank and base subtracted data.

The nonlinearity, f, was chosen to recreate features of the electrophysiological recordings. First, it was found that the odor-related perturbations are equally likely to be positive or negative (of all the significant neuron–odor pairs, 46% are positive in the AON and 48% in the APC); therefore, we chose a nonlinearity that was symmetric around zero. Negative activity does not mean the neurons have a negative firing rate but that their firing rate is inhibited relative to their response to base plus blank. Second, consistent with previous findings (Poo and Isaacson, 2009; Bolding and Franks, 2017; Iurilli and Datta, 2017), the cortical response to odors was relatively sparse. Therefore, we chose the shrink function as follows:

$$f(h_{o,i})=\left\{ \begin{array}{cc} h_{o,i}-\varphi & if\;h_{o,i}\geq \varphi \\ 0 & if\;|h_{o,i}|<\varphi \;,\\ h_{o,i}+\varphi & if\;h_{o,i}\leq -\varphi \end{array}\right.$$

where *ϕ* is a threshold that controls sparseness.

Based on previous work, we then modeled the connectivity matrix between the bulb and the cortex to be completely random (Schaffer et al., 2018) with elements either equal to 0, with probability 1−ξ, or drawn independently from a zero mean distribution with finite variance $\frac{\sigma^{2}}{N_{x}}$, with probability *ξ*. *ξ*, therefore, controls the sparseness of the matrix (note that negative weights do not mean that OB inputs are directly inhibitory; they rather model OB inputs projecting to inhibitory OC neurons, which in turn project to our OC neurons of interest). Now by central limit theorem, *h*_o,i_ is distributed in a Gaussian manner and with zero mean.

Since input to each cortical neuron is statistically independent, to fully specify its distribution, we must simply set the odor–odor covariance matrix of the Gaussian. Under the assumption that the size of the OB output response is identical for each odor ( $|x_{o}{|}^{2}=|x_{o^{\prime }}{|}^{2}\;\;\forall \;$odors o and o′), which can be justified by the divisive normalization that appears to happen in the OB (Zhu et al., 2013; Banerjee et al., 2015), the elements of the covariance matrix can be found as follows:

$$E_{i}[h_{o,i}^{2}]=\xi \sigma^{2}\frac{1}{N_{x}}|x_{o}{|}^{2}=\gamma^{2},$$

$$E_{i}[h_{o,i}h_{o^{\prime },i}]=\xi \sigma^{2}\frac{1}{N_{x}}x_{o}.x_{o^{\prime }}=\gamma^{2}\cos(\theta_{o,o^{\prime }}),$$

where $E_{i}$ refers to an expectation over the cortical population and we defined a new variable $\gamma^{2}:=\xi \sigma^{2}\frac{1}{N_{x}}|x_{o}{|}^{2}$ that controls the variance, and $\theta_{o,o^{\prime }}$ is the angle between two glomerular representations. For the 15 odors under consideration, we used calcium imaging to extract the cosine similarity which serves as the Gaussian covariance. To complete our ipsilateral specification, we must, therefore, only choose *γ* and a threshold *ϕ*—we shall rationalize these terms in two ways.

The threshold controls sparseness of cortical response; we match this to the AON recordings. The sparseness of the recordings is measured as the proportion of neuron–odor pairs for which the Wilcoxon signed-rank test *p* value comparing the set of seven trials to mean blank odor response is below 5% (22.4% ipsilateral, 25.2% contralateral). Labeling the sparseness as *S*, because of the assumed Gaussianity of cortical inputs, we get the following:

$$S=\frac{1}{\sqrt{2\pi \gamma^{2}}}\left(\int_{\phi }^{\infty }\;e^{-\frac{h^{2}}{2\gamma^{2}}}\mathrm{dh}+\int_{-\infty }^{-\phi }\;e^{-\frac{h^{2}}{2\gamma^{2}}}\mathrm{dh}\right)=\mathrm{erfc}\left[\frac{\phi }{\gamma \sqrt{2}}\right],$$

where erfc is the cumulative error function. Therefore, the sparseness sets the ratio of threshold to variance (Extended Data Fig. 5-1) as follows:

$$\frac{\phi }{\gamma }=\sqrt{2}\mathrm{erf}c^{-1}[S].$$

Finally, we used the ipsilateral distribution of significant response magnitudes (i.e., the absolute values of the significant responses) in the AON (Fig. 2*E*, top panel) to fit the conversion of numbers to the firing rate (Hz), *γ*. Our model predicts the significant response magnitude distribution to be the tail of a Gaussian; by changing *γ* we are simply rescaling the *x* axis of this distribution; we chose the *γ* that permits the best model-data fit. This fully specified the ipsilateral simulations and fit the data remarkably well (Fig. 6; Extended Data Fig. 6-1).

To compare ipsilateral to contralateral responses, we created two ipsilateral responses according to the scheme above. One of these was then mapped through a cross-cortical connectivity matrix to create a contralateral response. We used this contralateral response and the second ipsilateral creation for our comparisons to mice.

Our final task, therefore, was to design a cross-cortical connectivity matrix. In the body of the paper, we used a simple Hebbian structured matching of each odor between hemispheres. Here we include two additional connectivity schemes that are low-dimensional approximations to $G_{\mathrm{Struct}}$. The first uses SVD to create a low-rank matrix closest to $G_{\mathrm{Struct}}$ as measured by the Frobenius norm. The second uses the principal components to create $G_{\mathrm{Struct}}$ using a smaller number of Hebbian matching experiences. These show that the particular form of the structured connectivity is not important, as long as they tie together the important subspaces in the two hemispheres. In each case we combine the structured matrix with a random matrix and examine how much structure is required to fit the observed data.

The random component has elements drawn from a zero mean normal distribution with Variance 1 (this choice of variance is unimportant as the matrix will be rescaled in future) as follows:

$$G_{\mathrm{ij}}^{\mathrm{Rand}}∼N(0,1),$$

with probability ξ, else 0.

The structured Hebbian component maps from contralateral to ipsilateral odor responses using the covariance rule formulation that arises from Hebbian synaptic plasticity (Dayan and Abbott, 2001) as follows:

$$G_{\mathrm{Struct}}^{\mathrm{Unscaled}}=\overset{P}{\underset{o=1}{\sum }}\;(Y_{o}^{Ipsi}-C^{Ipsi})(Y_{o}^{Opp}-C^{Opp}{)}^{T},$$

where $Y_{o}^{Ipsi}$ is the response vector to odor o presented ipsilaterally in the ipsilateral cortex, $C^{Ipsi}$ is the response in the ipsilateral cortex averaged over odors, *p* = 15 is the total number of odors, and $Y_{o}^{Opp}\;$is the response in the opposite cortex to an odor presented in the opposite nostril.

These two matrices currently have outputs that are of arbitrary magnitudes. To ensure each contribution has a roughly similar sized output, the structured section is rescaled by a factor as follows:

$$G_{\mathrm{Struct}}={\chi G}_{\mathrm{Struct}}^{\mathrm{Unscaled}}\;\mathrm{where}\;\;chi=\frac{\Sigma_{o=1}^{P}|G_{\mathrm{Rand}}Y_{o}^{\mathrm{Opp}}|}{\Sigma_{o=1}^{P}|G_{\mathrm{Struct}}^{\mathrm{Unscaled}}Y_{o}^{\mathrm{Opp}}|},$$

before being combined using the parameter *α* to create a cross-cortical link with varying levels of randomness/structure as follows:

$$G=\alpha G_{\mathrm{Struct}}+(1-\alpha )G_{\mathrm{Rand}}.$$

Since the size of the outputs of each matrix is equal, we can now interpret *α* as the proportion of contralateral input magnitude that is formed by structured connectivity.

The magnitude of the elements of this matrix is then scaled again such that the resulting contralateral representation has the desired sparseness when the same piriform threshold is applied as was used to create the ipsilateral sparseness. Finally, the conversion to hertz, *γ*, is used to compare with data.

We varied the *α* and looked at how four measures of cross-cortical alignment changed (Fig. 6*E*–*J*; see main text for details) and how simulations compared with our actual AON data. We chose the optimal *α* to be the point with the minimum *z*^2^-score across the four—the smallest squared sum of sigmas (error bars) between the point and the AON data line in all four plots.

We modeled our AON with 50,000 neurons, this is a rough underestimate based on other paleocortical neuronal densities (Srinivasan and Stevens, 2017) and measurements of AON size from the Allen Brain Atlas. Our model, however, was robust to changes in number of neurons, for example, we tried a 25,000-neuron version (Extended Data Fig. 6-1*E*–*H*), and the optimal *α* did not change.

Our first low-rank approximation uses singular value decomposition to factorize $G_{\mathrm{Struct}}$ as follows:

$$G_{\mathrm{Struct}}=U\Sigma V^{T},$$

where *U* and *V* are orthogonal matrices and *Σ* is a diagonal matrix. We vary the dimensionality of our approximation through setting all but the largest *Q* singular values in *Σ* to 0, creating Σ* which we then use to recreate $\;G_{\mathrm{Struct}}$:

$$G_{\mathrm{Struct}\;2}^{\mathrm{Unscaled}}=U\Sigma^{*}V^{T}.$$

The last type of structured connectivity is a simple extension, comprising a Hebbian matching of vectors between the two hemispheres. However rather than matching the odors explicitly, we try to align the odorant subspace in which the odorants sit. To do this, we calculate the principal components of the contralateral representation and design a connectivity scheme that aligns the first few of the contralateral principal components between cortices as follows:

$$G_{\mathrm{Struct}\;3}^{\mathrm{Unscaled}}=\overset{Q}{\underset{q=1}{\sum }}\;Y_{q}^{Ipsi}Y_{q}^{OppT},$$

where *Q* is the number of principal components used, $Y_{q}^{Opp}$ is the *q*th principal component of the opposite cortical representation, and $\;Y_{q}^{Ipsi}$ is the ipsilateral representation of the same mixture of odors that comprises $Y_{q}^{Opp}$**.** More explicitly, the *q*th principal component in the opposite cortex is a mixture of normalized odorant vectors as follows:

$$Y_{q}^{Opp}=\overset{P}{\underset{o=1}{\sum }}\;\rho_{o}^{q}Y_{o}^{Opp},$$

where $\rho^{q}$ is the vector of coefficients for the *q*th principal components. We then use the same set of coefficients to create $Y_{q}^{Ipsi}$as follows:

$$Y_{q}^{Ipsi}=\overset{P}{\underset{o=1}{\sum }}\;\rho_{o}^{q}Y_{o}^{Ipsi}.$$

The dimensionality of each approximation, *Q*, must be chosen such that most of the response variation is captured; however, the low dimensionality of the responses means high alignment can be achieved despite a small *Q*. For example, in Extended Data Figure 6-2, *A* and *B*, we show how cross-cortical decoding accuracy varies with the dimensionality of each approximate structured matrix. Only seven singular values (Extended Data Fig. 6-2*C*–*F*), or six principal components (explaining 73% of the variance; Extended Data Fig. 6-2*G*–*J*), are needed for the alignment metrics to reach the values seen in neuronal recordings.

### Quantification and statistical analysis

All quantifications and statistical analyses were performed using custom scripts in MATLAB (MathWorks), except (1) the circular statistical analyses, which were performed with the CircStat toolbox written by Philipp Berens (Berens, 2009), and (2) Hartigan's dip tests, which were performed with the code written by Ferenc Mechler (Mechler and Ringach, 2002). The linear regressions were obtained with the least square method. The data are given as median ± SD, unless specified otherwise. For all statistical tests, the critical value was set to 5%. All statistical tests were two-sided, with the exception of the bootstrap strategies (where we asked whether the measurements were significantly higher than chance).

Additionally, the significance of the linear regressions was tested using either an *F* test (Fig. 3; Extended Data Figs. 3-1, 3-2). The significance of the percentage of bilaterally correlated neurons (BCNs) was tested using a bootstrap strategy (see figure caption for details). Extended Data Table 1-2 summarizes all the statistical tests performed in this study.

10.1523/ENEURO.0155-24.2024.f3-1 Figure 3-1 **Bilaterally-Correlated Neurons**. Related to Figure 3. (A) to (D) Percentage of bilaterally-correlated neurons per mouse. (A) Identical to Figure 3B. (B) Same as Figure 3B, except we kept only the neurons responding to at least one odor on one side for this analysis. (C) Same as Figure 3B, except we kept only the neurons responding to at least one odor on each side for this analysis (note that the odors eliciting responses can be different on each side). (D) Correlation across artificially-generated trials. For each mouse, each side, each neuron, each odor, two sets of 7 artificial trials were generated using a Poisson process. Then for each side, an analysis similar to Figure 3B was performed, except we looked for correlations between the first and second sets of 7 artificial trials, instead of correlations between ipsi and contralateral trials. A full circle means the percentage is higher than chance (significance is determined with a bootstrapping method similar to Figure 3C). Panel (D) allows us to estimate the apparent percentage of bilaterally-correlated neurons one would expect if all neurons were bilaterally-correlated. (E) Upper estimates of bilateral correlations in ideal conditions. For each mouse, each side, each neuron, we randomly split the 7 trials into two groups of 3 and 4, to calculate the correlation between the two groups of trials. We then computed the percentage of bilateral correlations among all neurons. We repeated this process for all the possibilities of trial splits. The + signs to the right of each distribution show the actual values from Figure 3B for comparison. The goal of this test is to estimate an upper bound for the percentage of bilateral correlations one could expect in each mouse. (F) Spike traces from mouse PPC1. The average spike of all the neurons recorded from APC1 are shown. The spike amplitudes were normalized for display. Note the apparent bimodal distribution of spike widths. (G) Histograms of the spike widths at half-maximum amplitude, per OC region. For each histogram, a Hartigan’s dip test was performed to confirm that the distribution of spike widths was not unimodal (AON p = 0.020, APC p = 0.016). The corresponding p-values are reported next to each graph. We also used a maximum likelihood estimate approach to fit a mixture of two Gaussian distributions to each distributions (AON: weight of the 1st Gaussian relative to the mixture w = 0.07, μ1 = 85.6, σ1 = 4.5, μ2 = 130.1, σ2 = 22.9; APC: w = 0.11, μ1 = 94.2, σ1 = 8.1, μ2 = 146.3, σ2 = 26.2). For each distribution, the vertical bar indicates spike width at which the Gaussian mixture reaches its minimum between the two peaks: we chose this value as the definition for “narrow” and “broad” spikes (AON 95μs; APC 110μs). Using this criterion, we found 85% of broad spikes in the AON and 89% in the APC. (H) Basal firing rate of the neurons with narrow versus broad spikes. Based on the histograms in (G), spikes were categorized as narrow or broad. White circle and value next to it: median. Thick bar: quartiles. Thin bar: range of values. The neurons with narrow spikes showed a significantly higher basal activity than the broad ones. This finding, along with the presence of a bimodal spike width distribution, suggests that the neurons with narrow spikes are mostly inhibitory, while the neurons with larger spikes are mostly excitatory. The purple bar shows the same analysis performed on the bilaterally-correlated (noted “cor”) neurons of the AON and APC (Kruskal-Wallis test across the “narrow”, “broad’, and “cor” categories: p = 3.2e-6; post-hoc Wilcoxon rank-sum test, “narrow” versus “broad” p < 0.0001, “narrow” versus “cor” p = 0.0008, “broad” versus “cor” p = 0.60). (I) Comparison of the spike width distribution of all AON and APC neurons versus bilaterally-correlated neurons. Unlike the overall distribution in the AON and APC, the spike width distribution for the bilaterally-correlated neurons is not significantly different from a unimodal distribution (Hartigan’s dip test, p = 0.06) Almost all the bilaterally-correlated neurons have a broad spike (97% neurons have a broad spike), suggesting they are putative excitatory neurons. (J) PID signal amplitude at different odor dilutions. All amplitudes are reported relative to the amplitude of the dilution used for the rest of this study (5%). Each grey dot is one PID measurement (10 repeats per condition), the black dots are the average amplitudes. For each odor, the red dot indicates the dilutions at which the PID signal is about 10 times weaker than the initial 5% dilution. (K) Response magnitude of the initial versus weaker dilutions. Each graph is one side of the mask. Each dot is one neuron-odor pair (n = 2 mice recorded in the AON, 120 neurons). We only tested the 6 odors shown in panel I (initial dilution: 5%; new dilution: see red dot in I). Only the neuron-odor pairs significantly responding to the initial 5% concentration are shown. Red: average for each side. When we stimulated the same side of the mask with 10 times less odorant, we obtained neuronal responses about 6 to 7 times weaker than with our initial 5% concentration. Therefore, cross-contamination in the mask, if any, cannot explain the presence of side-invariant odor responses. Download Figure 3-1, TIF file.

10.1523/ENEURO.0155-24.2024.f3-2 Figure 3-2 **Correlation of Population Odor Representations**. Related to Figure 3. (A) and (B) Ipsi- versus contralateral responses per odor in both OC regions tested. (A) AON. (B) APC. Red line: linear regression (contra = a x ipsi + b). Solid lines show significant regressions (F-test, p < 5%). Odor # is the same as Figure 1-1A. Each dot is one neuron. The top left graph for each region is also shown in Figure 3G. (C) Same as panel (A), odor 1, except neuron identity has been randomly shuffled on each side. Note the absence of significant correlation. (D) Significance of the regression slope for panel (A), odor 1. We built a chance distribution by repeating the shuffling shown in panel (C) 1000 times. We then compared the actual value of the slope to this distribution to determine its significance. Here we show the distribution of chance slopes for the AON, odor 1. The results of our bootstrap strategy matched the results of the F-test mentioned above. (E) We performed the same analysis as panels (A) and (B), but we looked at each mouse separately. Here we report the R2 value of each regression, as well as whether the correlation was significant (F-test). N.s. means that the correlation was not statistically significant (p ≥ 5%). Download Figure 3-2, TIF file.

10.1523/ENEURO.0155-24.2024.f3-3 Figure 3-3 **Variability Across Mice**. Related to Figures 3 and 4. In this figure we point at factors that may explain the variability we observed across mice in terms of percentage of bilaterally-correlated neurons, as well as odor decoding accuracy. (A) Mean number of odors eliciting response (related to Figure 2E). (B) Mean response magnitude (related to Figure 2F). (C) Percentage of bilaterally-correlated neurons (related to Figure 3B). Solid dot: significant percentage. (D) Mean accuracy of the odor decoding process with model trained on responses to ipsilateral presentations (related to Figure 4-1B). (E) Similar to (D) with training on contralateral presentations. Download Figure 3-3, TIF file.

### Data and code availability

All data and code used to compute quantities presented in this study is available on the Murthy lab GitHub page: https://github.com/VNMurthyLab/IpsiContra_v2.

## Results

### A new method for unilateral odor delivery

A major goal of this study was to compare the bilateral odor receptive fields of OC neurons of awake mice. To do so, we needed a way to reliably deliver odors to one nostril at a time. Previous studies have used two different techniques to achieve such unilateral odor stimulations. Either a piece of tubing was inserted in each nostril (Wilson, 1997) or the two nostrils were separated by a plastic septum (Kikuta et al., 2008, 2010). Since mice have very sensitive muzzles, neither of these approaches is applicable to awake animals.

We developed a new facial mask for unilateral odor delivery in awake mice (Fig. 1*B*). On each side of the mask, close to the nostrils, we used a computer-controlled olfactometer to deliver 15 monomolecular odors, one at the time, to the left or the right nostril (Extended Data Fig. 1-1*A*,*B*). The mask was connected to an airflow sensor for respiration monitoring (Extended Data Fig. 1-1*C*–*F*). We aligned all neuronal odor-evoked activity to the first sniff after odor onset (see Materials and Methods).

We tested the side selectivity of the mask through two methods. First, we simultaneously monitored the glomerular activity at the surface of the OBs of OMP-GCaMP3 mice (Isogai et al., 2011) through calcium imaging while delivering odors to either nostril (Fig. 1*C*; *n* = 155 glomeruli, six OBs, three mice). When odors were presented on the contralateral nostril, glomeruli showed little to no response (Fig. 1*D*–*F*).

We also performed extracellular recordings of M/T cells in the OB of awake mice using chronically implanted tetrodes (*n* = 42 single units isolated from four mice; Fig. 1*G*; Extended Data Fig. 1-1*G*–*L*; Extended Data Table 1-1). M/T cells hardly responded to contralateral odor presentations: out of the 42 isolated neurons, 32 responded to ipsilateral stimulations, while 10 showed significant contralateral responses (Fig. 1*H*; Extended Data Fig. 1-1*M*). Furthermore, while the ipsilateral M/T cell responses were largely positive, the few contralateral responses were always negative (Fig. 1*H*).

The ratio of contra- to ipsilateral response magnitude elicited by odors presented within the mask was 0.12 in our glomerular imaging data (Δ*F*/*F* magnitude; average ± SEM; ipsilateral, 7.9 ± 0.025%; contralateral, 0.93 ± 7.0 × 10^−3^%; Fig. 1*I*) and 0.10 in our M/T recordings (response magnitude; average ± SEM; ipsilateral, 1.6 ± 0.019 Hz; contralateral, 0.16 ± 2.1 × 10^−3^ Hz; Fig. 1*J*). Therefore, on average, odor responses were 8–10 times stronger on the ipsilateral side of the mask, compared with the contralateral side. In addition, by systematically lowering the concentration of odor stimuli presented to the two nostrils, we found that the glomerular responses to contralateral stimuli were comparable with presenting a 10-fold lower-intensity ipsilaterally (Extended Data Fig. 1-3). Finally, we further evaluated potential cross-contamination by imaging the glomerular activity of awake mice after naris occlusion while presenting odors through our mask (Extended Data Fig. 1-4). This experiment confirmed our other controls: when considering the odor-evoked activity of the OB contralateral to the occlusion (i.e., the OB on the unoccluded side), the glomerular responses were >10 times when stimulating the open nostril, compared with puffing odors on the occluded naris (Extended Data Fig. 1-4*C*).

Previous work has indicated that M/T cell responses to a given odor at a concentration of 0.1% were quite similar to the same odor at a concentration of 1% (Bolding and Franks, 2017, 2018). Since the M/T cell responses we observed are substantially smaller for contralateral presentations, the effective odor concentration in the ipsilateral nostril, when odors are presented contralaterally, is likely to be much <10% of the initial concentration from ipsilateral presentations.

Together, these results confirmed that odors, when delivered to one nostril through our custom-built mask, activate sensory neurons in the other nostril at least an order of magnitude less than ipsilateral activation.

### Unilateral odor tuning of neurons in different regions of the OC

We next used our unilateral odor delivery system to characterize how the OC responds to unilateral odor presentations. To do so, we monitored the activity of the AON and APC through extracellular recordings in awake mice (*n* = 7 mice; 1,316 neurons total; AON, 3 mice, 385 neurons; APC, 4 mice, 931 neurons; Fig. 2*A*; Extended Data Fig. 2-1; Extended Data Table 1-1).

Overall, neurons in the OC had low spontaneous activity (Fig. 2*B*), in accordance with previous studies (Litaudon et al., 2003; Zhan and Luo, 2010; Bolding and Franks, 2017). Spontaneous activity in the AON was significantly higher than in the APC (average spontaneous activity ± SEM; AON, 3.9 ± 0.012 Hz; APC, 2.8 ± 3.6 × 10^−3^ Hz; rank-sum test; *p* < 0.0001).

We found a diversity of responses to ipsi- as well as contralateral odor stimulations in both the AON and APC, including neurons that responded to odors presented to either one or the other nostril or to both nostrils (Fig. 2*C*,*D*). Importantly, in both OC regions, a sizeable fraction of neurons responded to contralateral odor presentations (fraction of neurons significantly responding to at least one odor presented on the ipsilateral nostril; AON, 86%; APC, 81%; contralateral side, AON, 87%; APC, 83%), expanding earlier anesthetized observations to the awake OC (Wilson, 1997; Kikuta et al., 2008, 2010).

OC neurons typically responded to at most a few odors from our odor panel (Fig. 2*E*), as previously reported with bilateral odor stimulations (Bolding and Franks, 2017; Iurilli and Datta, 2017). We found a wide range of response magnitudes across the OC, and contralateral responses were as strong as ipsilateral ones in the APC and slightly weaker in the AON (Fig. 2*F*; Wilcoxon signed-rank test, ipsi- vs contralateral distribution; AON, *p* = 8.2 × 10^−3^; APC, *p* = 0.94).

An idiosyncratic feature of olfactory cortical responses is that a neuron that responds to a particular odor with an increase in the firing rate tends to exhibit increases in firing rates for any other odor it responds to; similarly, a neuron having inhibitory response to an odor is also inhibited by any other odor it responds to (Kikuta et al., 2008; Zhan and Luo, 2010; Otazu et al., 2015; Bolding and Franks, 2017; Hu et al., 2017). We find that this feature applies to both cortical regions we examined (Fig. 2*G*).

Overall, we found reliable odor responses in the OC to unilateral stimulation through both the ipsi- and contralateral nostrils.

### Strong bilateral correlations in the OC

Our data indicate that the OC receives robust contralateral odor information, but it is unclear whether this information is similar to that arriving ipsilaterally. To examine this issue, we related the average odor-evoked responses to ipsilateral against contralateral presentations.

First, we plotted the ipsi- versus contralateral odor responses for each neuron individually. Given the prevalent view of OC connectivity as largely random, we expected OC neurons to show little to no match between their ipsi- and contralateral odor response profiles. To our surprise, we found many neurons with bilaterally correlated odor responses (Fig. 3*A*; *F* test; significance of the linear model, from left to right neuron; top row, *p* = 0.41; *p* = 0.84; *p* = 0.54; *p* = 0.59; bottom row, *p* = 5.3 × 10^−6^; *p* = 5.5 × 10^−4^; *p* = 6.6 × 10^−3^; *p* = 2.2 × 10^−4^; see also Fig. 2*D*, neurons with an asterisk). Overall, for each mouse recorded in the OC, we consistently found a sizeable fraction of bilaterally-correlated neurons (BCNs) (Fig. 3*B*).

We used a bootstrap strategy to determine that the fraction of BCNs in each mouse was significantly higher than what one would expect from chance. In brief, for each mouse, each neuron, we randomly shuffled odor identities on each nostril separately. We repeated this shuffling for all neurons and calculated the resulting percentage of BCNs. We reiterated this procedure 10,000 times in order to build a “chance” distribution. Finally, we compared this distribution with the actual fraction of BCNs. In all mice recorded in the OC, the fraction of BCNs was greater than one would expect from chance (Fig. 3*B*,*C*; Extended Data Fig. 3-1*A*–*D*).

While our initial estimates of the fraction of BCNs include all neurons recorded (Fig. 3*B*; Extended Data Fig. 3-1*A*), we verified that our conclusions remained true when selecting only the significantly responding (Extended Data Fig. 3-1*B*,*C*).

We performed additional analysis to determine the extent to which response variability masks response correlations. We generated individual trials from a Poisson process with rates determined from our recordings. We then enquired how correlated responses would be for two independent sets of seven trials generated with the same set of data. A large number of such sampling revealed that the fraction of (simulated) neurons that show significant response correlations was on the order of 40–80%. Therefore, even when excluding virtually all variability sources except for the neurons’ trial-to-trial variability, we still cannot reach a perfect correlation (100%). In other words, response variability probably lowers the apparent bilateral correlations we observe (Extended Data Fig. 3-1*D*). This analysis indicates that the fraction of significantly correlated neurons in the AON observed in our recordings may underestimate the real fraction of BCNs.

In addition, for each mouse, each side, and each neuron, we randomly split the seven trials into two groups of three and four, to calculate the correlation between the two groups of trials. We then computed the percentage of bilateral correlations among all neurons. We repeated this process for all the possibilities of trial splits. The goal of this test was to estimate an upper bound for the percentage of bilateral correlations one could expect in each mouse. The correlations obtained through this method were visually similar to the actual percentages. This suggests that the variability across mice in the actual data comes, at least partly, from the trial-to-trial response variability and/or overall weak odor responses, rather than a sizable difference of fraction of BCNs across mice. The analysis also suggests that the percentages of BCNs that were obtained in each mouse are rather close to the maximum correlation one could expect (Extended Data Fig. 3-1*E*).

Overall, the bilateral correlations were strong (Fig. 3*D*), with mostly positive slopes (Fig. 3*E*), and a distribution of intercepts centered on zero (Fig. 3*F*). In other words, BCNs appear to respond to unilateral odor presentations in a mostly side-invariant way, with their ipsi- and contralateral odor responses similar in sign and amplitude. The distribution of slopes was similar between the bilateral correlations originating from the AON and APC (mean ± SEM; AON, 0.79 ± 0.025; APC, 0.69 ± 0.036; Wilcoxon rank-sum test; *p* = 0.25). In addition, we found minimal but significant differences between the distribution of coefficients of determination (mean ± SEM; AON, 0.60 ± 0.017; APC, 0.54 ± 0.016; Wilcoxon rank-sum test; *p* = 0.013), as well as intercepts (mean ± SEM; AON, −0.24 ± 0.11; APC, 0.37 ± 0.16; Wilcoxon rank-sum test; *p* = 3.9 × 10^−3^) between the two regions.

We wondered if the bilaterally matched responses occurred preferentially in a particular neuron type. We used a classic method for identifying putative excitatory and inhibitory neurons—the width of extracellular action potentials (Suzuki and Smith, 1985; Gur et al., 1999; Frank et al., 2001; Swadlow, 2003; Povysheva et al., 2006; Hassani et al., 2009; Weir et al., 2015). Most of the BCNs in the AON and APC had broader spikes and lower spontaneous activity (Extended Data Fig. 3-1*F*–*I*), suggesting they are excitatory.

When testing our mask for cross-contamination (Fig. 1), we estimated that at most 10% of odors puffed on one side of the mask could reach the contralateral nostril. Can this cross-contamination alone explain the side-invariant responses we observed in the OC? To test this hypothesis, we first determined how much each odor from our panel needed to be diluted in the solvent to elicit a PID signal 10 times weaker than our initial dilution (5% volume/volume; Extended Data Fig. 3-1*J*). We then recorded individual neurons in the AON of awake mice (*n* = 2 mice; 120 neurons) while delivering odors on either side of the mask, both at initial (5%) and new dilutions (i.e., dilutions resulting in PID signals 10 times weaker; Extended Data Fig. 3-1*K*). On average, for a given side of the mask, new dilutions elicited neuronal responses 6–7 times weaker than our initial dilutions (neuronal response; mean ± SEM; for ipsilateral presentations; initial dilution, 2.1 ± 0.023 Hz; final dilution, 0.34 ± 2.7 × 10^−3^ Hz; for contralateral presentations; initial dilution, 2.5 ± 0.031 Hz; final dilution, 0.37 ± 2.5 × 10^−3^ Hz). In other words, since weak ipsilateral presentation generates responses that are much weaker than the contralateral responses we measured, contralateral cross-contamination in the mask, if any, is not sufficient to explain the presence of side-invariant odor responses.

Finally, we compared the ipsilateral and contralateral population representation in the two OC regions recorded for each odor (Fig. 3*G*; Extended Data Fig. 3-2*A*–*C*). Consistent with our analysis of individual BCNs, we found that unilateral population representations were significantly and strongly correlated for all odors tested in the AON and APC (Extended Data Fig. 3-2*A*,*B*; see Extended Data Table 1-2 for detailed statistics). Analyzing population correlations for each odor individually ensures that the correlations we observe are not driven by few neurons responding strongly and similarly to all odors, which would otherwise create an artificially strong positive correlation. Furthermore, a bootstrap analysis similar to what we described above confirmed that the strong correlation we observed for each odor individually was unlikely to occur by chance (Extended Data Fig. 3-2*C*,*D*). When considering each mouse separately, our conclusions held true for most odors (Extended Data Fig. 3-2*E*).

Together, these data indicate that the odor information arriving contralaterally to the OC strongly matches that arriving ipsilaterally.

### Accurate decoding of odor identity from contralateral activity

Odor selectivity in the OC allows a population representation of odor identity, and odor identity can be decoded from the activity of a sufficient number of neurons (Bolding and Franks, 2017). If odor responses are similar for ipsi- and contralateral presentations, decoding of identity should be transferrable. To formally test this, we asked a linear classifier to decode odor identity from OC responses to stimuli in one nostril, after we trained it with OC responses to stimuli in the opposite nostril.

In brief, at each instance we created a training set by randomly removing one trial per odor (Bolding and Franks, 2017). If we were testing on the same side as we trained (e.g., train ipsilaterally, test ipsilaterally), then we tested the classifier on the trials outside of the training set. If we were testing on the opposite side (e.g., train ipsilaterally, test contralaterally), then one trial per odor from the opposite side was randomly chosen to form the test set (Fig. 4*A*). We performed all our decoding analyses over an odor response window of 2 s, as it maximized performance of odor decoding on the same side (Extended Data Fig. 4-1*A*).

10.1523/ENEURO.0155-24.2024.f4-1 Figure 4-1 **Ipsi- and Contralateral Odor Decoding**. Related to Figure 4. (A) Decoding accuracy with varying odor response time windows. For each data point, response vectors were built by summing spikes from 0  s to the time indicated on the x-axis (responses aligned to the first sniff after odor onset, see Methods for details). This example shows the odor decoding for all neurons in the AON, train ipsi, test ipsi. Odor decoding accuracy is maximal at 2  s, therefore we decided to perform all our decoding analysis over an odor response window of 2  s. (B) Same as Figures 4B-4C, except the decoding process was applied to each mouse separately. Accuracy at n = 50 neurons is significantly higher than chance for all mice. The mice with poorer decoding accuracy were usually the ones in which neurons showed fewer / weaker responses on average, as well as fewer bilaterally-correlated neurons (see Figure 3-3). Download Figure 4-1, TIF file.

In both the AON and APC, the decoder performed with similarly high levels of accuracy when tested on data from the same or the opposite side (Fig. 4*B*,*C*). In other words, odor identity decoding optimized with responses to stimulation from one nostril transfers very well to stimulation of the other side.

Note that the seeming decrease of decoding performance from AON to APC is most probably due to variability across mice (Extended Data Fig. 4-1*B*). More precisely, the three mice with overall weaker and fewer odor-evoked responses (i.e., mice AON2, APC1, and APC2) showed lower odor decoding performances, no matter the side tested (Extended Data Fig. 3-3). In addition, AON2 is the mouse from which we isolated the fewest neurons (73 neurons, while all other OC recorded mice have over a hundred neurons each; Extended Data Table 1-1). Predictably, in these same three mice, the fractions of BCNs, while significantly higher than chance, were the lowest.

Rodents are able to determine the direction of the odor stimulation at short distances (Rajan et al., 2006; Rabell et al., 2017). We asked whether population activity in a single hemisphere differs sufficiently for stimulation of ipsi- and contralateral nostril to allow decoding of intensity differences between the two sides. Side identity, or laterality, could indeed be decoded with high accuracy (Fig. 4*D*), which confirms that odor representations from both sides, despite similarities, remain distinct and separable.

In addition, we asked whether the way we compute odor responses could influence the decoder's results. To do so, we replicated the decoding results from Figure 4*B*,*D*, using odor responses calculated with no blank trial subtraction (Extended Data Fig. 4-2). Overall, the decoding results obtained with and without blank subtraction are very similar, suggesting that the way we built our initial metric had little impact on the decoder's performance.

10.1523/ENEURO.0155-24.2024.f4-2 Figure 4-2 **Ipsi- and Contralateral Odor Decoding with No Blank Subtraction**. Related to Figure 4. We replicate the decoding results from Figure 4B-D, using a new metric to compute odor responses with no blank trial subtraction. More precisely, we performed decoding on odor responses R calculated using the following formula: $R=\overset{7}{\underset{t=1}{\sum }}\;(Dur_{o}^{t}-Bef_{o}^{t})/7$ (see Methods for the significance of each symbol). The goal of this analysis is to verify that the way we compute odor responses does not influence the decoder’s results. (A) Similar to Figure 4B-C (odor decoding). (B) Similar to Figure 4D (side decoding). Download Figure 4-2, TIF file.

Finally, we tested how BCNs affected the decoding results, by performing the decoding analysis described in Figure 4 while varying the proportion of BCNs in the dataset fed to the decoder (Extended Data Fig. 4-3). Decoding performance degraded as we removed BCNs from the dataset, which indicates that BCNs are the main drivers of the high performances of our odor decoder. In addition, same-side decoding was also affected by the removal of BCNs. This suggests that BCNs carry a sizable fraction of the total odor information, presumably because of stronger responses. Lastly, side identity could be decoded with high accuracy, even in the absence of BCNs, which confirms that the odor response profiles from both sides remain distinct, even in the noncorrelated population.

10.1523/ENEURO.0155-24.2024.f4-3 Figure 4-3 **Bilaterally-Correlated Neurons and Decoding Performance**. Related to Figure 4. (A) Same as Figure 4B, except we removed all the bilaterally-correlated neurons before computing the accuracies. (B) For each decoding, we randomly took 135 neurons, including a certain fraction of bilaterally-correlated neurons (as indicated on the y-axis). For each region, each fraction of bilaterally-correlated neurons, each test side, we repeated this decoding 50 times, each time with a new set of randomly-picked neurons. The box plots show the median (white and black dot), 1st and 3rd quartiles (thick bar), 95% confidence interval (thin bar), and the outliers (black circles). All training data used for this panel are from the ipsilateral side. (C) Same as panel (A), for side decoding instead of odor decoding. (D) Same as panel (B), for side decoding instead of odor decoding. (E) Same as panel (A), except the analysis was performed on each individual mouse. (F) Same as panel (B), except the analysis was performed on each individual mouse. Here, for each decoding, instead of 135 neurons, we took N neurons, N being the total number of bilaterally-correlated found in each mouse. For all panels, the gray dotted line shows chance level. Download Figure 4-3, TIF file.

Together, our data indicate that odor decoding is transferrable across sides. Ipsi- and contralateral odor representations, while distinct, share a high degree of similarity.

### A computational model of bilateral matching in the OC

How does one OC respond so similarly to inputs from two distinct routes/nostrils? So similarly, in fact, that a linear classifier trained on one set of representations can decode the other?

**Figure 5.**
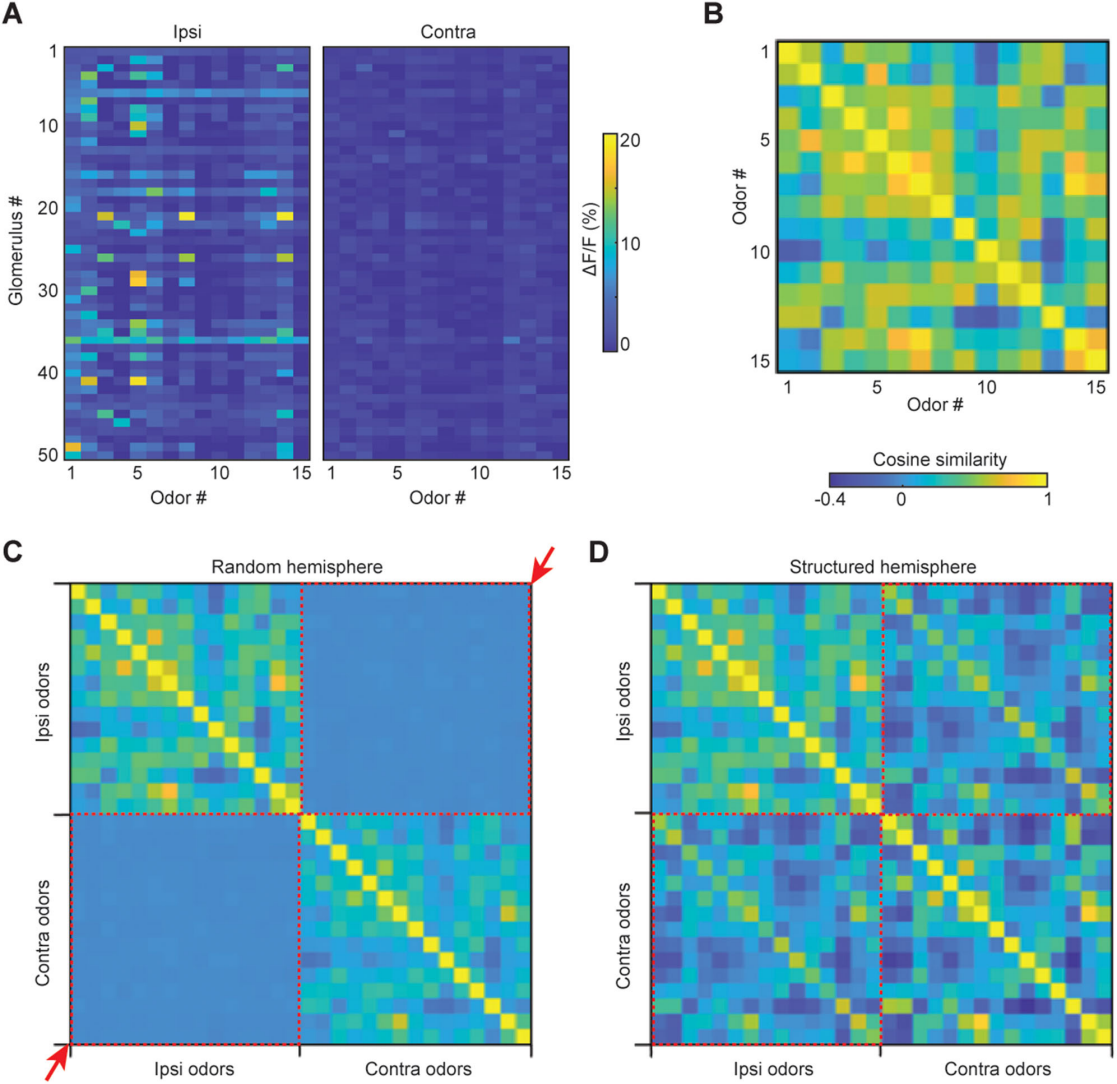
Structure is necessary for alignment between responses to different nostrils. ***A***, Odor tuning of 50 randomly selected glomeruli. ***B***, The OB odor–odor cosine similarity matrix measured using calcium imaging. Color scale at right applies to panels ***B***–***D***. ***C***, We modeled the two OCs and connected them using a random matrix (see Materials and Methods), the correlations between odors from the same nostril are preserved, but correlations between nostrils are completely lost (quadrants pointed to by red arrows and red dotted squares). ***D***, Including a structured cross-cortical connectivity produces correlations between ipsi- and contralateral odors. See also Extended Data Note 5-1.

An appealingly simple resolution might suggest itself based on recent theoretical work. It has been shown that if two correlated representations are projected through the same random matrix, the output representations are also correlated (Babadi and Sompolinsky, 2014; Schaffer et al., 2018). Thus, one could hypothesize that any interhemispheric projection between two OCs could preserve the structure inherited from the underlying OB representations, hence explaining the similarity. However, this argument is insufficient, as illustrated in Figure 5. The correlations in glomerular neural representations of the 15 odors used in our study are shown in Figure 5*B*. These correlations are partially preserved when projected through two different random matrices (to represent the OB to OC projections of each hemisphere; Fig. 5*C*, top and bottom block diagonals). However, the two OC hemispheres connected with random projections will not yield correlated responses to the same odor presented to the left or right side (Fig. 5*C*). In the extended data (Extended Data Note 5-1) we show that, even in the limiting case where the response of ipsi- and contralateral cortices are identical, unstructured interhemispheric connectivity ensures that the responses of one cortex to odors presented in different nostrils will be uncorrelated. Hence, there must exist some structured connectivity that is aligning the cortical responses (Fig. 5*D*; note the much higher similarity values in the two off-diagonal blocks).

10.1523/ENEURO.0155-24.2024.f5-1 Figure 5-1 **Visualization of the Relationship Between Phi and Gamma.** Related to Figure 5. This figure shows a heatmap representation of the relationship between gamma, phi, and S, as shown in equation 7. In addition, we provide variance equivalents to the gamma values, for an exemplar data set with the following characteristics: 50 glomeruli, 15% chance that a given neuron-odor pair is significantly responding, magnitude of average odor response 1.6  Hz. Download Figure 5-1, TIF file.

10.1523/ENEURO.0155-24.2024.d1 Extended Data Note 5-1 Random Connections Produce Zero Correlations – a Derivation. Download Extended Data Note 5-1, DOCX file.

We then asked what type of structured interhemispheric connectivity is sufficient to achieve the observed tight alignment of responses from different nostrils. Through computational modeling, we tested a simple and plausible model for structured matching, a Hebbian connectivity matrix that links together the same-odor representations in each hemisphere. We combined this Hebbian matrix (*G*_Struct_) with a random matrix (*G*_Rand_), which models unstructured elements of connectivity and whose elements are drawn independently from a Gaussian distribution. We vary the degree of structure using a parameter *α*. Explicitly,

$$G=\alpha G_{\mathrm{Struct}}+(1-\alpha )G_{\mathrm{Rand}}.$$

The parameter *α* took values between 0 and 1 with *α *= 0 corresponding to purely random connectivity and *α *= 1 to purely structured connectivity. The random and Hebbian components were scaled appropriately such that *α* represents the proportion of contralateral input magnitude that is formed by structured connectivity.

**Figure 6.**
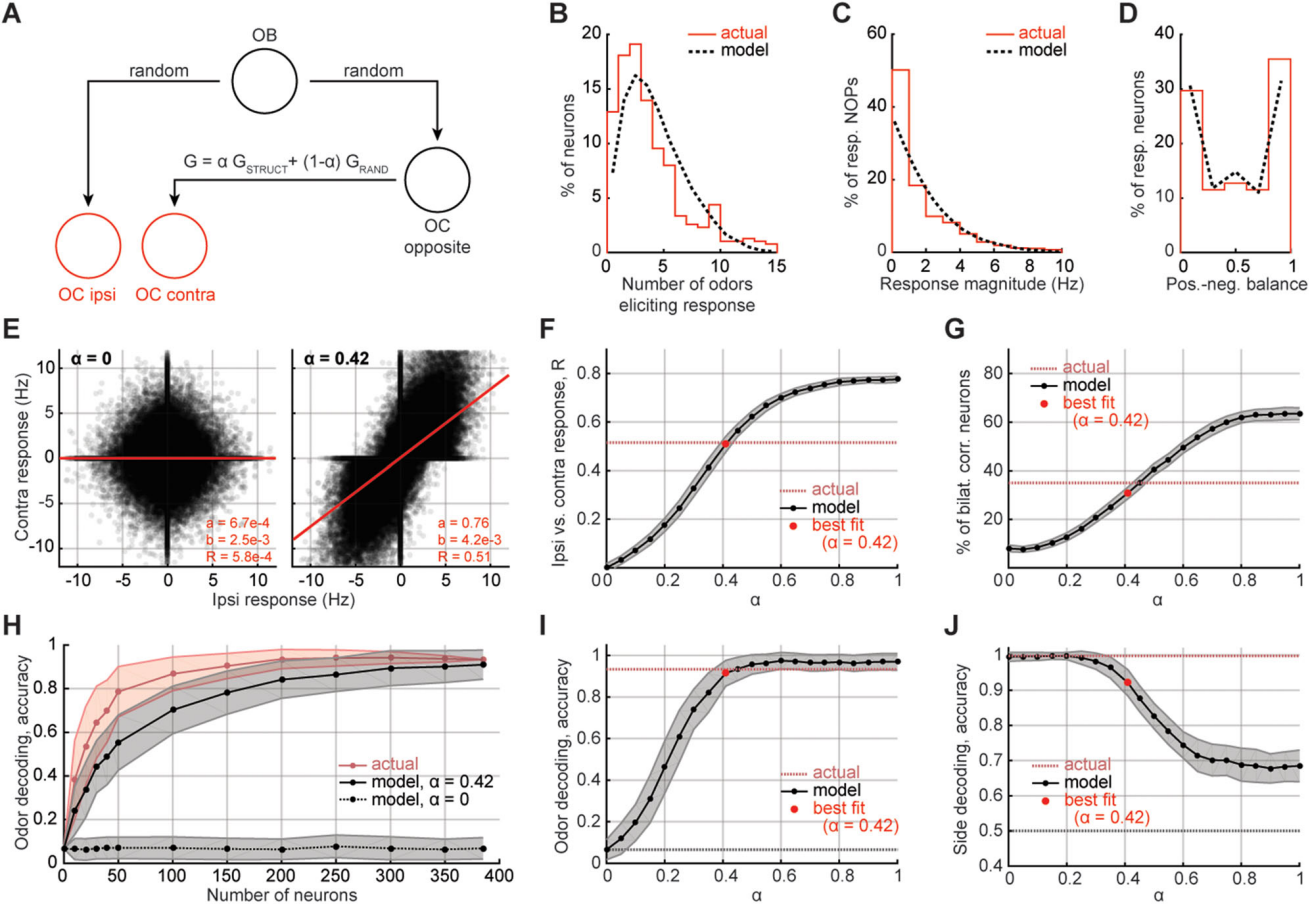
Modeling olfactory cross-cortical connections. ***A***, Diagram of the model. ***B–D***, Model validation. Contralateral odor responses from the model are overlaid with actual contralateral AON responses (Extended Data Fig. 2*E–G*). ***E–J***, Various measurements of cross-cortical alignment from the model. ***E***, Contra- versus ipsilateral responses for random (*α* = 0) and semistructured (*α* = 0.42) projections. ***F***, Contra- versus ipsilateral response correlations R for various *α* values. ***G***, The percentage of BCNs for various *α* values. ***H***, Odor decoding accuracy for random (*α* = 0) and semistructured (*α* = 0.42) projections. Red, Same as Figure 3*B*, AON, “test contra.” ***I***, Odor decoding accuracy for various *α* values. ***J***, Side decoding accuracy for various *α* values. For ***E*–*H***, measurements from the model were calculated by sampling 385 neurons 200 times. The best fit *α* (0.42) was found by comparing to AON data (*n* = 385 neurons from 3 mice). See also Extended Data Figures 6-1 and 6-2.

Our modeling strategy began by creating a cortical representation of odors presented in the ipsilateral nostril, based on the AON. Random OB-to-OC connectivity indicates that each cortical olfactory neuron samples a random subset of glomeruli. This implies that the input to each cortical neuron is statistically independent and that the response of one cortical neuron to different odorants can be modeled as a thresholded multivariate Gaussian whose covariance matrix can be extracted from activities in the OB (Fig. 6*A*; see Materials and Methods). We validated this model of a single AON against neuronal recordings: our model replicated the population statistics remarkably well with minimal parameter adjustment (Fig. 6*B*–*D*; Extended Data Fig. 6-1*A*–*C*).

10.1523/ENEURO.0155-24.2024.f6-1 Figure 6-1 **Modeling Olfactory Cross Cortical Connections**. Related to Figure 6. (A) to (C) Same as Figures 6B-6D, except for we are comparing the distributions of mouse ipsilateral data and our simulations of ipsilateral responses with optimal alpha (0.42). Mouse data is identical to the contralateral lines from Figures 2E-2F, top panels. (D) Same as Figure 6I, except we decoded ipsilateral odors using the contralateral presentations. (Left) Using closest Euclidean neighbor decoding is markedly worse in simulations than mice, due mainly to the difference in magnitude of responses that results as you increase the structure parameter. (Right) Using closest angular neighbors instead recaptures better performance, and the best alpha overlaps with the AON performance. This suggests that, in the left panel, the decoder limited performance rather than lack of information. (E) to (H) Same as Figures 6F, 6G, 6I, and 6J respectively, except each OC layer contains 25,000 neurons (instead of 50,000 neurons in Figure 6, see Methods). These show that the optimal alpha level (0.42) is robust to changes in the number of cortical neurons simulated. Download Figure 6-1, TIF file.

10.1523/ENEURO.0155-24.2024.f6-2 Figure 6-2 **Low-dimensional structured connectivity is sufficient to produce alignment.** Related to Figure 6. (A) and (B) Odor decoding accuracy using 385 neurons as Q is varied, averaged over 200 iterations of decoding and 10 different creations of G_Struct, (A) approximates using SVD and (B) using PCA. (C) – (F) are the same as Figure 6F, G, I, and J but using an approximate G_Struct created with 7 singular values. (G) – (J) are the same but using 6 PCA components. Download Figure 6-2, TIF file.

We then linked two simulated AONs using the connectivity matrix *G*. The computational analog of the cortical representations of an odor presented contralaterally is the representation after mapping through the interhemispheric matrix, *G*. Thus, by comparing the simulated and measured alignment of ipsi- and contralaterally presented odors as we vary *α*, we can ask if this simple Hebbian structure is sufficient to recapture the degree of alignment observed.

We compared the alignment using four metrics: the overall correlation of odor population representation across sides (Fig. 6*E*,*F*; similar to Fig. 3*G*), the fraction of BCNs (Fig. 6*G*; similar to Fig. 3*B*), the accuracy of odor identity decoding (Fig. 6*H*,*I* and Extended Data Fig. 6-1*D*; similar to Fig. 4*B*,*C*), and the accuracy of side identity decoding (Fig. 6*J*; similar to Fig. 4*D*).

The four computed measurements best matched our actual AON neuronal recordings with a nonzero *α* (∼0.4). This result held true, even when we fitted each animal separately (AON1 *α* = 0.3; AON2 *α* = 0.1; AON3 *α* = 0.3; APC1 *α* = 0.1; APC2 *α* = 0.1; APC3: *α* = 0.25; APC4 *α* = 0.35). In summary, for all animals, α was greater than zero.

This result did not vary when we changed the size of the modeled OC population (Extended Data Fig. 6-1*E*–*H*). This suggests that a significantly Hebbian connectivity between cortices is sufficient to explain the observations.

We chose this particular structured connectivity, the 15 odor Hebbian matrix *G*_Struct_, for its appealing simplicity and biological plausibility. For example, early in life, the animal might have smelt these odors in both nostrils and associatively matched the responses between hemispheres. However, there are many other sufficient alignment schemes that do not rely on having experienced specifically these 15 odors; we implemented two such examples in the supplementary material. The first uses a set of Hebbian matching experiences that span the subspace in which the 15 odorants lie. Roughly six of these are needed to achieve similar levels of alignment (Extended Data Fig. 6-2*G*–*J*) showing that a smaller number of more general early learning experiences could also explain the alignment seen in the data. The second technique uses singular value decomposition to create a low-rank approximation of *G*_Struct_; again, a rank of about 7 is necessary to match the data (Extended Data Fig. 6-2*C*–*F*). Effectively, any connectivity that links same-odor representations in each hemisphere together will be able to match the alignment seen in the data.

## Discussion

In many animals, odor information collected by the two nostrils remains separate until it reaches secondary brain areas (Bojsen-Moller and Fahrenkrug, 1971; Eccles, 2000; Kikuta et al., 2008). How the two streams of information are integrated, as well as the functional role of this integration, remain unclear.

Here, we reveal that a sizeable fraction of neurons in the OC are bilaterally correlated, meaning that they have matched ipsi- and contralateral odor tuning. Functional alignment of responses was also confirmed by the ability to decode odor identity from neural populations trained using only odor responses from the opposite nostril. Finally, our computational model suggests that such bilateral matching requires structured interhemispheric projections. Our findings shed light on how brains make sense of this bilateral experience in the olfactory system.

### Importance of the integration of bilateral information

In many senses, information is collected through a pair of sensors and is integrated within the brain. Bilateral perception allows us to perform vital behavioral computations such as sound localization or depth perception. Since the two nostrils may sample independent pockets of air (Wilson and Sullivan, 1999; Eccles, 2000; Kikuta et al., 2008), can bilateral information be used to discover features of the odor stimulus not readily available to single nostrils? Animals may compare odor concentration across both nostrils to locate odor sources (Catania, 2013; Parthasarathy and Bhalla, 2013; Gire et al., 2016; Rabell et al., 2017), which requires bilateral integration and comparison of different signals. Two brain regions that seem to fulfil these criteria are the AON and APC (Wilson, 1997; Kikuta et al., 2008, 2010). Recent studies have indicated that the AON may be sensitive to the differential activation of left and right nostrils, therefore computing the direction of the stimulus in front of the animal (Rabell et al., 2017). Our own data show that side identity can be decoded from odor responses, which provides further evidence for differential processing (Fig. 4*D*).

In contrast, many behaviors require the animal to make inferences independent of the nostril stimulated. One simple example can be found in our everyday life: one nostril is usually more blocked than the other, and which nostril gets clogged changes several times a day (Eccles, 2000). Nevertheless, we can identify smells and have a unified olfactory experience. In the same fashion, animals need to be able to identify relevant smells like food or a predator, no matter where the odor source is relative to their nose. Indeed, there is evidence for nostril-independent recognition of odors: rats trained to associate odors with reward using only one naris can generalize to stimuli delivered through the other nostril (Kucharski and Hall, 1987, 1988; Yan et al., 2008). This behavior requires interhemispheric communication, suggesting a convergence of ipsilateral and contralateral representations in higher brain areas (Dalal et al., 2020).

### Robust contralateral odor responses in the OC

Here, we developed and tested a new method to deliver odor stimuli selectively to individual nostrils in awake, freely breathing mice (Fig. 1). We performed several experiments to confirm selective stimulation. First, PID recordings indicated that odors were delivered at the same concentration on either side and are not detectable on the side contralateral to the stimulus. Second, direct measurements of sensory input to the OB indicated that glomerular responses to contralateral stimulation were below detection threshold. It is possible that some low-level cross-contamination went undetected by the PID and glomerular recordings, but it is unlikely that such weak inputs could contribute to contralateral responses that are as strong as those to ipsilateral stimulation, as shown by our recordings in AON to 10-fold diluted odors (Extended Data Fig. 3-1*K*).

Third, recordings from putative M/T cells in the OB using tetrodes revealed few detectable responses to contralateral nares stimulation. These results seem at odds with a recent report suggesting that topographic projections from contralateral AONpE to M/T cells support bilaterally matched odor responses (Grobman et al., 2018). A possible source of the discrepancy is the much lower concentration of odor used in our mask. However our observations confirm former work from various teams regarding the near absence of contralateral M/T response in the OB (Wilson, 1997; Kikuta et al., 2008, 2010). Our results are also consistent with the known projections from the AON pars externa to the GABAergic granule cells of the contralateral OB (Schoenfeld and Macrides, 1984; Yan et al., 2008), since the infrequent significant responses we observed in M/T cells to contralateral nostril stimulation were inhibitory. The minimal responses in glomeruli and M/T cells also argue strongly against bilateral return of odors from the respiratory system during exhalation.

A recent study in humans (Dikeçligil et al., 2023), published after completion of our study, strongly corroborates our findings. Intracranial electrocorticogram recordings in the piriform cortex from subjects presented with odors separately in each nostril revealed significant contralateral responses, which were delayed compared with ipsilateral ones. Nevertheless, the signals also exhibited odor identity-related correlations. These results complement our findings that ipsilateral and contralateral responses are correlated but have enough differences to allow decoding laterality.

### Bilateral integration in the OC is not random

Our first key finding is that responses to the stimulation of the contralateral nostril could readily be detected in the AON and APC (Fig. 2). This is not entirely surprising, since crossed projections already occur at the level of the AON and APC. Previous studies indicated that contralateral stimulations evoked fewer and weaker responses in the AON (Kikuta et al., 2008), and the responses from the two sides appeared to be significantly correlated. However, these studies reported a relatively small number of neurons in anesthetized animals, and the matching responses were characterized in terms of similarity of odor chemical categories, rather than firing rate responses. While we confirmed the presence of weaker and fewer responses to contralateral stimulations in the AON, the differences were smaller than previously reported. Another recent study implicated the AON in interhemispheric communication using a variety of methods, but the responses of neurons in this area were not measured (Yan et al., 2008). Finally, one previous study reported the presence of neurons responsive to contralateral naris stimulations in the APC of anesthetized rats, but did not test the correlation of the responses between the two nares (Wilson, 1997).

We found that the odor information arriving ipsilaterally to the OC strongly matches that arriving contralaterally (Fig. 3). Furthermore, our data offer strong hints that principal neurons are a major part of the ensemble of neurons that have matched responses to stimuli from both nostrils (Extended Data Fig. 3-1). This feature would allow downstream areas receiving olfactory cortical inputs to readily have access to side-stable odor responses. Indeed, we showed explicitly that odor decoding is transferable across sides (Fig. 4), as expected from the presence of matched responses in the OC.

Finally, we considered how the prevalent view of OC connectivity could explain our data (Fig. 5). Recent studies have shown that two representations projected through the same random matrix remain correlated (Babadi and Sompolinsky, 2014; Schaffer et al., 2018). In addition, another study reported that population response correlations between odors is shaped in the cortex, presumably by experience, such that some response similarities in the OB are enhanced and others are suppressed (Pashkovski et al., 2020). However, these studies do not address alignment due to projections between cortices. Here we implemented a computational model of the olfactory system to test matching of ipsilateral and contralateral odor tuning in the OC as a function of structure in bilateral projections. We showed that, in our model, purely random projections cannot explain our OC results. That is, even if the two hemispheres have the same ordered representations for similar odors, unstructured interhemispheric cortical projections will disrupt these correlations.

### Limitations and potential future directions

A key feature of our experimental design is the selective stimulation of either nostril; therefore, it is important to consider the possibility of cross-contamination of odors to other nostril such that responses to the intended contralateral stimulation may arise through ipsilateral neural pathway. While our tests with miniPID recordings indicated virtually no contralateral odor contamination in the mask (Extended Data Fig. 1-1*B*), the control experiments we performed in the OB suggest that up to 10% of an odor delivered to one nostril may be sampled by the other nostril within the mask (Fig. 1). One could argue that the ipsi- and contralateral stimulations could activate similar neuron populations solely by the potential odor cross-contamination reported here. In other words, the contralateral odor stimulations might act as diluted ipsilateral stimuli and, as such, activate the same OC neurons as when odors are presented ipsilaterally. The diluted ipsilateral stimuli might then be potentially boosted by the normalization mechanisms proposed in recent studies (Bolding and Franks, 2018; Stern et al., 2018), thus explaining the side-invariant responses we observed. However, we consider this unlikely because individual neurons in the APC have complex and often nonmonotonic tuning to odor concentration (Chae et al., 2022; Zak et al., 2023), such that responses to the same odor at different concentrations may not be strongly correlated. Finally, our control experiments revealed that responses in AON to 10-fold more dilute odors are highly attenuated (Extended Data Fig. 3-1*J*,*K*). Collectively, these features argue against contamination playing a significant role in the aligned responses, but we acknowledge this possibility as a limitation. Future experiments in mice with lesion of the anterior commissure linking the two OC hemispheres could help resolve this issue.

We were unable to extract any differences in the latency of responses for stimuli from the two sides. A major reason for this is likely to be the variable spiking responses and the inadequate control of odor delivery in relation to timing of sniffs. We note, however, that the small size of mouse brains and the known conduction and dendritic integration speeds may lead to very little delays—for example, the latencies of odor responses in the entorhinal cortex are barely different from those in the piriform cortex (Bitzenhofer et al., 2022).

We also acknowledge the variability in the number of correlated neurons across animals. Such differences could be due to a sampling issue, in which an overall lower responsiveness to odors in the finite sample in each animal could mask underlying correlations. It is also possible that there is genuine biological variability related to individual experiences of the animals. Larger recording samples in future studies, combined with manipulation of experiences, might help overcome such statistical limitations and uncover any biological variability.

Finally, we note that our simplified model considers dense connectivity across the two hemispheres, but the actual underlying connectivity might be sparse (Hagiwara et al., 2012). The model is presented as a plausible explanation and not as a proof, and future work must investigate the effectiveness and adequacy of sparse interhemispheric connectivity.

### Reconciling our results with known connectivity schemes

How can the responses of cortical neurons to selective stimulation of each of the two nostrils be matched? First, there may be topographically matched crossed projections somewhere in the olfactory system allowing bilateral response matching. The only known candidate for such matching is the projection from AON pars externa to OB (Schoenfeld and Macrides, 1984; Yan et al., 2008). This projection, however, should result in net inhibition of M/T cells since its major target are the GABAergic granule cells (Schoenfeld and Macrides, 1984; Yan et al., 2008; but see Grobman et al., 2018). Furthermore, previous studies (Wilson, 1997; Kikuta et al., 2008, 2010) and our own data (Fig. 1) suggest that this projection is too weak to support the widespread bilateral matching we observed in the OC. However, M/T cells that respond to contralateral odor presentations in a correlated way (Grobman et al., 2018) may report to ipsilateral OC neurons the same information for ipsi- and contralateral stimulation under some conditions, for example, high odor concentrations. Even so, unstructured interhemispheric cortical projections will disrupt this potential alignment in the cortex. Therefore, additional mechanisms, such as Hebbian plasticity, are needed to explain our observations.

A second way in which matched responses may arise is through fine-scale order in anatomical connectivity that somehow allows the same glomerular output channels from both OBs to converge on individual neurons. Such structure seems implausible and has no precedent but remains formally possible.

A third explanation that seems likely because of its simplicity and local nature is that synaptic connectivity in cortical regions is refined by Hebbian plasticity (Best and Wilson, 2003; Franks and Isaacson, 2005; Johenning et al., 2009) to allow matched responses from both sides. During their normal experience, mice will frequently inhale similar odors through both nostrils. This can allow associative plasticity in cortical areas to select for matched inputs from both hemispheres, similar to, for example, the well-studied map development in the visual system (Hubel and Wiesel, 1962; Bednar and Wilson, 2016; Gu and Cang, 2016). This hypothesis of experience-dependent matching of bilateral inputs to individual cortical neurons is open to experimental testing.

## Synthesis

Reviewing Editor: David Schoppik, New York University Grossman School of Medicine Decisions are customarily a result of the Reviewing Editor and the peer reviewers coming together and discussing their recommendations until a consensus is reached. When revisions are invited, a fact-based synthesis statement explaining their decision and outlining what is needed to prepare a revision will be listed below. The following reviewer(s) agreed to reveal their identity: Alexander Fleischmann.

Major points

1. Cross Contamination

While the authors present evidence that lateral cross contamination of odors in their face mask is minimal at most, any cross contamination undermines their findings; contralateral odor responses might arise from contamination, instead of from interhemispheric information transfer. While the authors made an attempt to validate the hemispheric isolation of the mask using PID measurements and neural recordings, much detail is still missing from their treatment of the calibration of this device.

The manuscript needs additional detail on the dimensions of the odor delivery mask. Specifically: the distance between odor inlets and vacuum outlets, the diameter of the device, the length of the tubing to the pressure monitoring outlet, etc. This information would permit reproduction and evaluation of the design and function. We recommend that the authors include the dimensions, ideally as part of the schematic in Figure 1B, as well as in the Methods. Additionally, information about the fittings, tubing diameter, and tubing material are all missing and would be very useful for someone reproducing the device. While the airflow into the device was 50 cc/min, the flow rate of the vacuum lines is also missing. This is very relevant for understanding the hemispheric isolation achieved by the device.

What is the actual cross-talk between the two nostrils? We do not imagine it is zero. Should the reader consider the concentration of the contralateral naris to be e.g. 10-fold lower than in ipsilateral naris?

We recommend that authors emulate a mouse's inhalation in order to simulate concentration conditions experienced in the experiment. One can position the PID suction tubing at approximately the same position as one of the mouse's nostrils relative to the center of the port, while also adding a second length of suction tubing to emulate the mouse's other nostril. At the peak of its inhalation, the airflow in the mouse's nose is approximately 0.5 L/min per naris, which is easy to emulate by adjusting the suction of both the PID and the second line to be 0.5 L/min each. In such a setup, one can record the PID value for both the contra and ipsilateral odor delivery to estimate the cross-talk between the nostrils under conditions that may more accurately simulate the experiment.

Alternatively, one can deliver an odor which evokes dorsal glomerular responses at very low concentrations (see Burton et al, eLife 2022), record glomeruli as in the manuscript, and compare the responses to a concentration series for contra and ipsilateral odor delivery. Matching contralateral responses at a higher concentration condition to ipsilateral responses at a lower concentration would allow for an estimate of the equivalent concentration to the contralateral cross-talk, which may be quite low, but nevertheless needs to be estimated to support the subsequent findings.

Finally, nasal occlusion is an established intervention that could be used to accurately quantify the magnitude of cross contamination in the face mask, or to present odors unilaterally in general. We recommend that the authors perform a small set of tetrode or calcium imaging recordings to compare neural activity in the presence or absence of naris occlusion. Furthermore, we recommend that authors discuss the differences between their unilateral odor delivery method and methods using naris occlusion.

2. Null model for bilaterally correlated neurons

The Poisson processes outlined in lines 453-460 should be explained in more detail, including the underlying motivation and null hypothesis used to compare with the experimental observations. For example, the authors should elaborate on why "the ideal response correlations should be 100%", when the Poisson processes generated are independent, and whether / why this is the null hypothesis to test for. The authors should support this with a few more panels for visualization / illustration in the extended figures, in addition to Extended Figure 3-1D. Moreover, the authors should include more details in Methods, e.g. whether this is a homogeneous or heterogeneous Poisson process, with or without refractory period.

We recommend that the authors compare correlations between ipsi/contra population responses with correlation between ipsi/ipsi (or contra/contra) population responses from the data without the need to model. One could intuit that the latter would set an upper bound for the former. To do this, the authors could split trials of ips into 2 separate sets randomly and then compute correlations between these sets multiple times. This may be useful to assess animal variability issues as well.

3. Animal variability and bilateral correlated neurons

The authors discuss limitations regarding animal variability, along with some analyses in extended figures. However, it is difficult to grapple with 4 animals having good population decoding with high % bilateral correlation neurons, and 3 animals having very bad population decoding with low % bilateral correlated neurons. Consequently, pooling the animals to form a pseudo-population may not represent the general decoding performance.

Below are some suggestions to investigate it further by analyzing existing data, or in the Discussion.

a) The features/response metrics are constructed using the blank trials. The authors should inspect whether the blank trials are "equal" across animals, e.g. whether they are on average at the same rate as pre-stimulus baseline rate. The authors can also remove blank trial normalization or perform different normalization (e.g. instead of "R_o - AVR(R_b)" do "(R_o - AVR(R_b)) / STD(R_b)" or z-score "R_o" only) to examine potential effects of these blank trials, as well as to normalize activity across neurons being feed into decoders.

b) The authors can use the model in Fig 6 to fit each animal separately, and look for how different parameters (alpha, or sparsity related parameters), or resulting properties (e.g. Fig 6, panels B-G) may be correlated with the decoding performance.

c) It seems that % bilaterally correlation neurons may be positively associated with both identity and laterality decoding performance. It would be interesting to test the potential causal relationship by gradually removing them from the population, both for the pseudo-population and for each animal separately, then inspecting the effects of such removal on the decoding performance. Similarly, it would be interesting to compare the difference of contribution between bilaterally correlated and uncorrelated neurons, by performing removal of only the latter neurons gradually while keeping the former neurons in the population.

d) The authors might investigate whether differential nasal cycling, well documented in humans but not in mice, explains any of their variability across animals. Nasal cycling within one animal would appear as changes in the symmetry of odors responses from session to session.

4. Neural recordings

Mitral/tufted cells: It surprised us to see odor responses with such a low rates (mean of 1.6 Hz) in mitral/tufted cells which tend to respond to odors in bursts of fairly high rates (10's of Hz). In Figure 1J it seems that even the strongest responders recorded still didn't exceed 6 Hz, which raises questions of how these experiments were performed and whether the odor responses recorded could be considered representative of how mitral/tufted cells would broadly encode ipsilateral vs contralateral odor stimuli. More detailed information about the recordings and analysis would be useful to be able to judge the lateral isolation of the odor delivery and the generality of the findings to other mitral/tufted cells. The manuscript would benefit from PSTHs, spontaneous rates, and to know the time window when firing rate changes were estimated.

The cortical recordings are similarly concerning. The odor response rates of PC and AON neurons are considerably lower than reported in other studies (i.e, Bolding &Franks, Science, 2018). The authors make it clear that average odor responses are computed with respect to a pre-odor baseline and responses to the 'blank' condition, yet it is still unclear what is being averaged. Is the measurement reported a spike count of the excess spikes within a 2 second window (compared to baseline and blank conditions) summed over trials and divided by the number of trials (7)? If so, it is unclear how to interpret a response on the order of 1-2 Hz. While statistically significant over a very limited number of trials, is a cell producing one excess spike per second of odor delivery meaningfully tuned to a given odor? How would an analysis excluding weak odor responses, or setting them to zero, change the results? Please consider &address these issues.

5. Computational Model

Why is the matrix computed for bilateral alignment referred to as a Hebbian matrix? There is no Hebbian learning in the model, and this matrix appears to be a projection matrix that maps each contralateral odor response vector to its correspondent ipsilateral response vector. While the resultant matrix could be the result of Hebbian learning in a dynamic model, I found this terminology confusing and expected to see some form of plasticity or learning rule explored in the model.

Minor points

1. Line 156-157: should it be "Wilcoxon rank-sum test" (or Mann-Whitney U test) rather than "Wilcoxon signed-rank test"?

2. Line 246: should it be "if h_{o,i} < -phi" for the last condition?

3. The authors should clarify the difference between a measure and magnitude of a measure in a few different cases, e.g. "dF/F" vs "dF/F magnitude".

4. The spelling of "%age" can be confusing in figures. The authors can use "percentage", "%" or "percent." as alternatives.

5. The equations should be labeled.

6. Fig 3-B and Table 1-1: "45/119" looks like from mouse AON3, which only has 118, so there may be a typo either in the table or the figure label.

7. To be consistent, use "G_struct" rather than "G_structured" and "G_struct".

8. Fig 5-C/D: y-axis labels should be "ipsi ... contra", not "contra ... ispi".

9. Red/blue color pairs are used in multiple different settings across all figures (pos/neg, ipsi/contra, AON/APC). At the same time, ipsi/contra sometimes is encoded by dash/solid lines or different markers. This can be confusing for readers. The authors can be more consistent by choosing red/blue for only one setting throughout (e.g. ips/contra), another simpler one (gray/black) for miscellaneous settings (e.g. pos/neg). Sometimes color scheme may not even be necessary, especially when they are not used in the same plot, e.g. Figs 2-4 for AON/APC.

10. Fig 2-C: since the response metric contains normalization with the blank trials, the authors should include them for illustration in the exemplar raster plot/PSTH.

11. Asterisks are conventionally reserved for statistical significance, instead of visually similarity as used in Fig 2-D. The authors can use other markers to indicate this to avoid confusion.

12. The dotted/dashed lines for chance-level (e.g. in Fig 4) should be darker / ticker to more easily distinguish with grid lines.

13. Equation in Line 273 could use an example visualization on the effect of these different parameters on sparsity (e.g. a heatmap with biologically-plausible ranges of these parameters), especially for biologists, to interpret this model better.

14. Consistency with comparison

The manuscript could use more consistent statistical significance analyses, for example sometimes statistical tests were performed between AON/APC in some panels (e.g. Fig 2-B/F) but not in others (e.g. Fig 2-E/G). Sometimes AON/APC are combined with each other without any comparison between them, e.g. Fig 3-D/E/F. Since the authors are already comparing between these 2 populations, the authors should be consistent and show comparison, as well as performing statistical tests where appropriate, between them.

15. In line 471, the authors state 'When testing our mask for cross-contamination (Fig. 1) we estimated that at most 10% of odors puffed on one side of the mask could leak to the other side.' This is not the argument made in the treatment of Fig. 1, which makes the claim that the concentration delivered to the ipsilateral nostril during contralateral delivery should be at most 10% of the concentration delivered to the contralateral side (and vice versa). Nowhere is it indicated that different odors have different propensities to leak across the device.

16. Line 1219-1223 &Extended Note 5: it's assumed that (a) J and G are random and (b) J and G are not correlated; and lines 1222-1223 implies from the observed data that the "cross-cortical connectivity must be structured". Is there anything in the manuscript that supports both assumptions?

17. Please consider discussing potential effects / consequences of recurrent connectivity within each olfactory cortex region and bilateral correlated neurons.

## References

[B1] Álvarez-Salvado E, Licata AM, Connor EG, McHugh MK, King BM, Stavropoulos N, Victor JD, Crimaldi JP, Nagel KI (2018) Elementary sensory-motor transformations underlying olfactory navigation in walking fruit-flies. Elife 7:e37815. 10.7554/eLife.37815 30129438 PMC6103744

[B2] Babadi B, Sompolinsky H (2014) Sparseness and expansion in sensory representations. Neuron 83:1213–1226. 10.1016/j.neuron.2014.07.035 25155954

[B3] Baker KL, Dickinson M, Findley TM, Gire DH, Louis M, Suver MP, Verhagen J V, Nagel KI, Smear MC (2018) Algorithms for olfactory search across species. J Neurosci 38:9383–9389. 10.1523/JNEUROSCI.1668-18.2018 30381430 PMC6209839

[B4] Banerjee A, et al. (2015) An interglomerular circuit gates glomerular output and implements gain control in the mouse olfactory bulb. Neuron 87:193–207. 10.1016/j.neuron.2015.06.019 26139373 PMC4633092

[B5] Bednar JA, Wilson SP (2016) Cortical maps. Neuroscientist 22:604–617. 10.1177/1073858415597645 26290447

[B6] Bekkers JM, Suzuki N (2013) Neurons and circuits for odor processing in the piriform cortex. Trends Neurosci 36:429–438. 10.1016/j.tins.2013.04.005 23648377

[B7] Berens P (2009) Circstat : a MATLAB toolbox for circular statistics. J Stat Softw 31:1–21. 10.18637/jss.v031.i10

[B8] Best AR, Wilson DA (2003) A postnatal sensitive period for plasticity of cortical afferents but not cortical association fibers in rat piriform cortex. Brain Res 961:81–87. 10.1016/S0006-8993(02)03847-7 12535779

[B9] Bitzenhofer SH, Westeinde EA, Zhang H-XB, Isaacson JS (2022) Rapid odor processing by layer 2 subcircuits in lateral entorhinal cortex. Elife 11:e75065. 10.7554/eLife.75065 35129439 PMC8860446

[B10] Blake R, Wilson H (2011) Binocular vision. Vision Res 51:754–770. 10.1016/j.visres.2010.10.009 20951722 PMC3050089

[B11] Bojsen-Moller F, Fahrenkrug J (1971) Nasal swell-bodies and cyclic changes in the air passage of the rat and rabbit nose. J Anat 110:25–37. 4110864 PMC1271026

[B12] Bolding KA, Franks KM (2017) Complementary codes for odor identity and intensity in olfactory cortex. Elife 6:e22630. 10.7554/eLife.22630 28379135 PMC5438247

[B13] Bolding KA, Franks KM (2018) Recurrent cortical circuits implement concentration-invariant odor coding. Science 361:eaat6904. 10.1126/science.aat6904 30213885 PMC6492549

[B14] Boyd AM, Sturgill JF, Poo C, Isaacson JS (2012) Cortical feedback control of olfactory bulb circuits. Neuron 76:1161–1174. 10.1016/j.neuron.2012.10.020 23259951 PMC3725136

[B15] Cang J, Feldheim DA (2013) Developmental mechanisms of topographic map formation and alignment. Annu Rev Neurosci 36:51–77. 10.1146/annurev-neuro-062012-170341 23642132

[B16] Catania KC (2013) Stereo and serial sniffing guide navigation to an odour source in a mammal. Nat Commun 4:1441. 10.1038/ncomms2444 23385586

[B17] Chae H, Banerjee A, Dussauze M, Albeanu DF (2022) Long-range functional loops in the mouse olfactory system and their roles in computing odor identity. Neuron 110:3970–3985.e7. 10.1016/j.neuron.2022.09.005 36174573 PMC9742324

[B18] Chang EH, Frattini SA, Robbiati S, Huerta PT (2013) Construction of microdrive arrays for chronic neural recordings in awake behaving mice. J Vis Exp 77:e50470. 10.3791/50470 23851569 PMC3731431

[B19] Cumming BG, DeAngelis GC (2001) The physiology of stereopsis. Annu Rev Neurosci 24:203–238. 10.1146/annurev.neuro.24.1.203 11283310

[B20] Dalal T, Gupta N, Haddad R (2020) Bilateral and unilateral odor processing and odor perception. Commun Biol 3:150. 10.1038/s42003-020-0876-6 32238904 PMC7113286

[B21] Dayan P, Abbott LF (2001) *Theoretical neuroscience: computational and mathematical modeling of neural systems*. Cambridge, MA: The MIT Press.

[B22] Dikeçligil GN, Yang AI, Sanghani N, Lucas T, Chen HI, Davis KA, Gottfried JA (2023) Odor representations from the two nostrils are temporally segregated in human piriform cortex. Curr Biol 33:5275–5287.e5. 10.1016/j.cub.2023.10.021 37924807 PMC13035057

[B23] Eccles R (2000) Nasal airflow in health and disease. Acta Otolaryngol 120:580–595. 10.1080/000164800750000388 11039867

[B24] Frank LM, Brown EN, Wilson MA (2001) A comparison of the firing properties of putative excitatory and inhibitory neurons from CA1 and the entorhinal cortex. J Neurophysiol 86:2029–2040. 10.1152/jn.2001.86.4.2029 11600659

[B25] Franks KM, Isaacson JS (2005) Synapse-specific downregulation of NMDA receptors by early experience: a critical period for plasticity of sensory input to olfactory cortex. Neuron 47:101–114. 10.1016/j.neuron.2005.05.024 15996551

[B26] Ghosh S, Larson SD, Hefzi H, Marnoy Z, Cutforth T, Dokka K, Baldwin KK (2011) Sensory maps in the olfactory cortex defined by long-range viral tracing of single neurons. Nature 472:217–220. 10.1038/nature09945 21451523

[B27] Giessel AJ, Datta SR (2014) Olfactory maps, circuits and computations. Curr Opin Neurobiol 24:120–132. 10.1016/j.conb.2013.09.010 24492088 PMC3913910

[B28] Gire DH, Kapoor V, Arrighi-Allisan A, Seminara A, Murthy VN (2016) Mice develop efficient strategies for foraging and navigation using complex natural stimuli. Curr Biol 26:1261–1273. 10.1016/j.cub.2016.03.040 27112299 PMC4951102

[B29] Grobman M, Dalal T, Lavian H, Shmuel R, Belelovsky K, Xu F, Korngreen A, Haddad R (2018) A mirror-symmetric excitatory link coordinates odor maps across olfactory bulbs and enables odor perceptual unity. Neuron 99:800–813. 10.1016/j.neuron.2018.07.012 30078580

[B30] Grothe B, Pecka M (2014) The natural history of sound localization in mammals–a story of neuronal inhibition. Front Neural Circuits 8:116. 10.3389/fncir.2014.00116 25324726 PMC4181121

[B31] Gu Y, Cang J (2016) Binocular matching of thalamocortical and intracortical circuits in the mouse visual cortex. Elife 5:e22032. 10.7554/eLife.22032 28033094 PMC5199194

[B32] Gur M, Beylin A, Snodderly DM (1999) Physiological properties of macaque V1 neurons are correlated with extracellular spike amplitude, duration, and polarity. J Neurophysiol 82:1451–1464. 10.1152/jn.1999.82.3.1451 10482761

[B33] Haberly LB, Price JL (1978) Association and commissural fiber systems of the olfactory cortex of the rat. I. systems originating in the piriform cortex and adjacent areas. J Comp Neurol 178:711–740. 10.1002/cne.901780408 632378

[B34] Hagiwara A, Pal SK, Sato TF, Wienisch M, Murthy VN (2012) Optophysiological analysis of associational circuits in the olfactory cortex. Front Neural Circuits 6:18. 10.3389/fncir.2012.00018 22529781 PMC3329886

[B35] Hassani OK, Lee MG, Henny P, Jones BE (2009) Discharge profiles of identified GABAergic in comparison to cholinergic and putative glutamatergic basal forebrain neurons across the sleep-wake cycle. J Neurosci 29:11828–11840. 10.1523/JNEUROSCI.1259-09.2009 19776269 PMC2790860

[B36] Hu R, Zhang J, Luo M, Hu J (2017) Response patterns of GABAergic neurons in the anterior piriform cortex of awake mice. Cereb Cortex 27:3110–3124. 10.1093/cercor/bhw175 27252353

[B37] Hubel DH, Wiesel TN (1962) Receptive fields, binocular interaction and functional architecture in the cat’s visual cortex. J Physiol 160:106–154. 10.1113/jphysiol.1962.sp006837 14449617 PMC1359523

[B38] Illig KR, Haberly LB (2003) Odor-evoked activity is spatially distributed in piriform cortex. J Comp Neurol 457:361–373. 10.1002/cne.10557 12561076

[B39] Isogai Y, Si S, Pont-Lezica L, Tan T, Kapoor V, Murthy VN, Dulac C (2011) Molecular organization of vomeronasal chemoreception. Nature 478:241–245. 10.1038/nature10437 21937988 PMC3192931

[B40] Iurilli G, Datta SR (2017) Population coding in an innately relevant olfactory area. Neuron 93:1180–1197. 10.1016/j.neuron.2017.02.010 28238549 PMC5370575

[B41] Johenning FW, Beed PS, Trimbuch T, Bendels MHK, Winterer J, Schmitz D (2009) Dendritic compartment and neuronal output mode determine pathway-specific long-term potentiation in the piriform cortex. J Neurosci 29:13649–13661. 10.1523/JNEUROSCI.2672-09.2009 19864577 PMC6664992

[B42] Kikuta S, Kashiwadani H, Mori K (2008) Compensatory rapid switching of binasal inputs in the olfactory cortex. J Neurosci 28:11989–11997. 10.1523/JNEUROSCI.3106-08.2008 19005064 PMC6671652

[B43] Kikuta S, Sato K, Kashiwadani H, Tsunoda K, Yamasoba T, Mori K (2010) Neurons in the anterior olfactory nucleus pars externa detect right or left localization of odor sources. Proc Natl Acad Sci 107:12363–12368. 10.1073/pnas.1003999107 20616091 PMC2901466

[B44] Kucharski D, Hall WG (1987) New routes to early memories. Science 238:786–788. 10.1126/science.3672125 3672125

[B45] Kucharski D, Hall WG (1988) Developmental change in the access to olfactory memories. Behav Neurosci 102:340–348. 10.1037/0735-7044.102.3.340 3395445

[B46] Litaudon P, Amat C, Bertrand B, Vigouroux M, Buonviso N (2003) Piriform cortex functional heterogeneity revealed by cellular responses to odours. Eur J Neurosci 17:2457–2461. 10.1046/j.1460-9568.2003.02654.x 12814377

[B47] Luo L, Flanagan JG (2007) Development of continuous and discrete neural maps. Neuron 56:284–300. 10.1016/j.neuron.2007.10.014 17964246

[B48] Mainland JD, Bremner EA, Young N, Johnson BN, Khan RM, Bensafi M, Sobel N (2002) Olfactory plasticity: one nostril knows what the other learns. Nature 419:802. 10.1038/419802a 12397347

[B49] Mechler F, Ringach DL (2002) On the classification of simple and complex cells. Vision Res 42:1017–1033. 10.1016/S0042-6989(02)00025-1 11934453

[B50] Miyamichi K, et al. (2011) Cortical representations of olfactory input by trans-synaptic tracing. Nature 472:191–196. 10.1038/nature09714 21179085 PMC3073090

[B51] Mori K, Nagao H, Yoshihara Y (1999) The olfactory bulb: coding and processing of odor molecule information. Science 286:711–715. 10.1126/science.286.5440.711 10531048

[B52] Nagayama S, Homma R, Imamura F (2014) Neuronal organization of olfactory bulb circuits. Front Neural Circuits 8:98. 10.3389/fncir.2014.00098 25232305 PMC4153298

[B53] Otazu GH, Chae H, Davis MB, Albeanu DF (2015) Cortical feedback decorrelates olfactory bulb output in awake mice. Neuron 86:1461–1477. 10.1016/j.neuron.2015.05.023 26051422 PMC7448302

[B54] Parthasarathy K, Bhalla US (2013) Laterality and symmetry in rat olfactory behavior and in physiology of olfactory input. J Neurosci 33:5750–5760. 10.1523/JNEUROSCI.1781-12.2013 23536088 PMC6705078

[B55] Pashkovski SL, Iurilli G, Brann D, Chicharro D, Drummey K, Franks KM, Panzeri S, Datta SR (2020) Structure and flexibility in cortical representations of odour space. Nature 583:253–258. 10.1038/s41586-020-2451-1 32612230 PMC7450987

[B56] Poo C, Isaacson JS (2009) Odor representations in olfactory cortex: “sparse” coding, global inhibition, and oscillations. Neuron 62:850–861. 10.1016/j.neuron.2009.05.022 19555653 PMC2702531

[B57] Porter J, Anand T, Johnson B, Khan RM, Sobel N (2005) Brain mechanisms for extracting spatial information from smell. Neuron 47:581–592. 10.1016/j.neuron.2005.06.028 16102540

[B58] Povysheva NV, Gonzalez-Burgos G, Zaitsev AV, Kröner S, Barrionuevo G, Lewis DA, Krimer LS (2006) Properties of excitatory synaptic responses in fast-spiking interneurons and pyramidal cells from monkey and rat prefrontal cortex. Cereb Cortex 16:541–552. 10.1093/cercor/bhj002 16033926

[B59] Rabell JE, Mutlu K, Noutel J, del Olmo PM, Haesler S (2017) Spontaneous rapid odor source localization behavior requires interhemispheric communication. Curr Biol 27:1542–1548.e4. 10.1016/j.cub.2017.04.027 28502658

[B60] Rajan R, Clement JP, Bhalla US (2006) Rats smell in stereo. Science 311:666–670. 10.1126/science.1122096 16456082

[B61] Reisert J, Golden GJ, Matsumura K, Smear M, Rinberg D, Gelperin A (2014) Comparing thoracic and intra-nasal pressure transients to monitor active odor sampling during odor-guided decision making in the mouse. J Neurosci Methods 221:8–14. 10.1016/j.jneumeth.2013.09.006 24056232 PMC3858470

[B62] Rey HG, Pedreira C, Quian Quiroga R (2015) Past, present and future of spike sorting techniques. Brain Res Bull 119:106–117. 10.1016/j.brainresbull.2015.04.007 25931392 PMC4674014

[B63] Schaffer ES, Stettler DD, Kato D, Choi GB, Axel R, Abbott LF (2018) Odor perception on the two sides of the brain: consistency despite randomness. Neuron 98:736–742. 10.1016/j.neuron.2018.04.004 29706585 PMC6026547

[B64] Schmitzer-Torbert N, Jackson J, Henze D, Harris K, Redish AD (2005) Quantitative measures of cluster quality for use in extracellular recordings. Neuroscience 131:1–11. 10.1016/j.neuroscience.2004.09.066 15680687

[B65] Schoenfeld TA, Macrides F (1984) Topographic organization of connections between the main olfactory bulb and pars externa of the anterior olfactory nucleus in the hamster. J Comp Neurol 227:121–135. 10.1002/cne.902270113 6470206

[B66] Scott JW, McBride RL, Schneider SP (1980) The organization of projections from the olfactory bulb to the piriform cortex and olfactory tubercle in the rat. J Comp Neurol 194:519–534. 10.1002/cne.901940304 7451680

[B67] Sosulski DL, Lissitsyna Bloom M, Cutforth T, Axel R, Datta SR (2011) Distinct representations of olfactory information in different cortical centres. Nature 472:213–216. 10.1038/nature09868 21451525 PMC3354569

[B68] Srinivasan S, Stevens CF (2017) A quantitative description of the mouse piriform cortex. bioRxiv 99002.

[B69] Stern M, Bolding KA, Abbott L, Franks KM (2018) A transformation from temporal to ensemble coding in a model of piriform cortex. Elife 7:e34831. 10.7554/eLife.34831 29595470 PMC5902166

[B70] Stettler DD, Axel R (2009) Representations of odor in the piriform cortex. Neuron 63:854–864. 10.1016/j.neuron.2009.09.005 19778513

[B71] Suzuki SS, Smith GK (1985) Burst characteristics of hippocampal complex spike cells in the awake rat. Exp Neurol 89:90–95. 10.1016/0014-4886(85)90267-5 4007119

[B72] Swadlow HA (2003) Fast-spike interneurons and feedforward inhibition in awake sensory neocortex. Cereb Cortex 13:25–32. 10.1093/cercor/13.1.25 12466212

[B73] Weir K, Blanquie O, Kilb W, Luhmann HJ, Sinning A (2015) Comparison of spike parameters from optically identified GABAergic and glutamatergic neurons in sparse cortical cultures. Front Cell Neurosci 8:460. 10.3389/fncel.2014.00460 25642167 PMC4294161

[B74] Wilson DA (1997) Binaral interactions in the rat piriform cortex. J Neurophysiol 78:160–169. 10.1152/jn.1997.78.1.160 9242270

[B75] Wilson DA, Sullivan RM (1999) Respiratory airflow pattern at the rat’s snout and an hypothesis regarding its role in olfaction. Physiol Behav 66:41–44. 10.1016/S0031-9384(98)00269-8 10222471

[B76] Wilson DA, Sullivan RM (2011) Cortical processing of odor objects. Neuron 72:506–519. 10.1016/j.neuron.2011.10.027 22099455 PMC3223720

[B77] Yan Z, Tan J, Qin C, Lu Y, Ding C, Luo M (2008) Precise circuitry links bilaterally symmetric olfactory maps. Neuron 58:613–624. 10.1016/j.neuron.2008.03.012 18498741

[B78] Zak JD, Reddy G, Konanur V, Murthy VN (2023) Distinct information conveyed to the olfactory bulb by feedforward input from the nose and feedback from the cortex. bioRxiv 2023.10.09.560787. 10.1038/s41467-024-47366-6 PMC11021479 38627390

[B79] Zhan C, Luo M (2010) Diverse patterns of odor representation by neurons in the anterior piriform cortex of awake mice. J Neurosci 30:16662–16672. 10.1523/JNEUROSCI.4400-10.2010 21148005 PMC6634870

[B80] Zhu P, Frank T, Friedrich RW (2013) Equalization of odor representations by a network of electrically coupled inhibitory interneurons. Nat Neurosci 16:1678–1686. 10.1038/nn.3528 24077563

