# Supplementary figures and images for: Bilateral Alignment of Receptive Fields in the Olfactory Cortex
Source: eNeuro. 2024 Nov 5;11(11):ENEURO.0155-24.2024. doi: 10.1523/ENEURO.0155-24.2024 (PMC11540595; doi:10.1523/ENEURO.0155-24.2024)

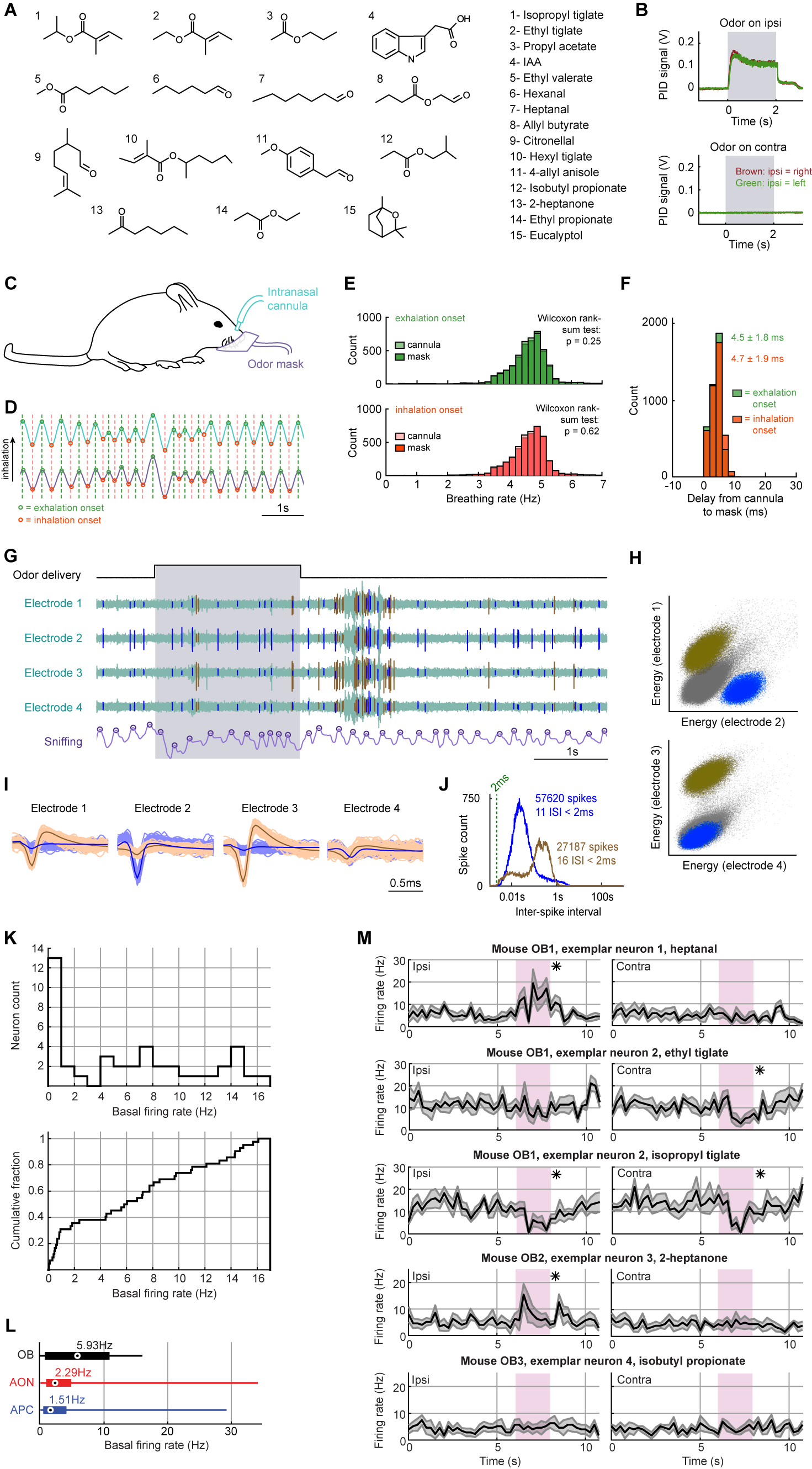

Supplement: Figure 1-1 — A New Method for Unilateral Odor Delivery. Related to Figure 1. (A) Odorants used in this study. (B) Control of the symmetry of the olfactometer. A PID measured the changes of odor concentration over time at the end of the right or the left end of the facial mask. Each measurement was repeated three times; therefore, each graph shows six over-imposed traces, three with the PID on the right, three on the left. Note the similarity between the traces obtained on the left and right of the mask, and the absence of odor detected on the contralateral side. Grey: odor presentation (here: isopropyl tiglate). All 15 odors were tested and showed similar results. (C) Diagram of the setup used for assessing the reliability of the face mask for respiration monitoring. The breathing of awake mice was monitored simultaneously with the mask and a pressure sensor connected to a chronic intranasal cannula (n = 3 animals, 15 min of continuous recording per mouse). (D) Exemplar breathing traces, simultaneously recorded from the intranasal cannula (light blue) and the face mask (purple). Note the synchrony of both signals and the apparent absence of phase shift. (E) Histogram of instantaneous breathing rates, calculated with the exhalation (top panel) or inhalation (bottom panel) onsets. The data shown here comes from the full monitoring session of one exemplar mouse. The distributions obtained from the intranasal cannula (light bars) or the face mask (dark bars) are almost identical. (F) Delay from cannula to mask, calculated with the exhalation (green) or inhalation (orange) onsets. The data from the three mice were combined here. Values: median ± SD. The phase shift between the intranasal cannula and the face mask is small and reliable. (G) Example of tetrode recording. The four electrodes belong to the same tetrode. Grey: odor delivery. Green: unsorted electrode traces. Blue and red: two single units. Purple: sniffing acquired simultaneously (up: inhalation). These exemplar traces we [file eneuro-11-ENEURO.0155-24.2024-s002.tif]

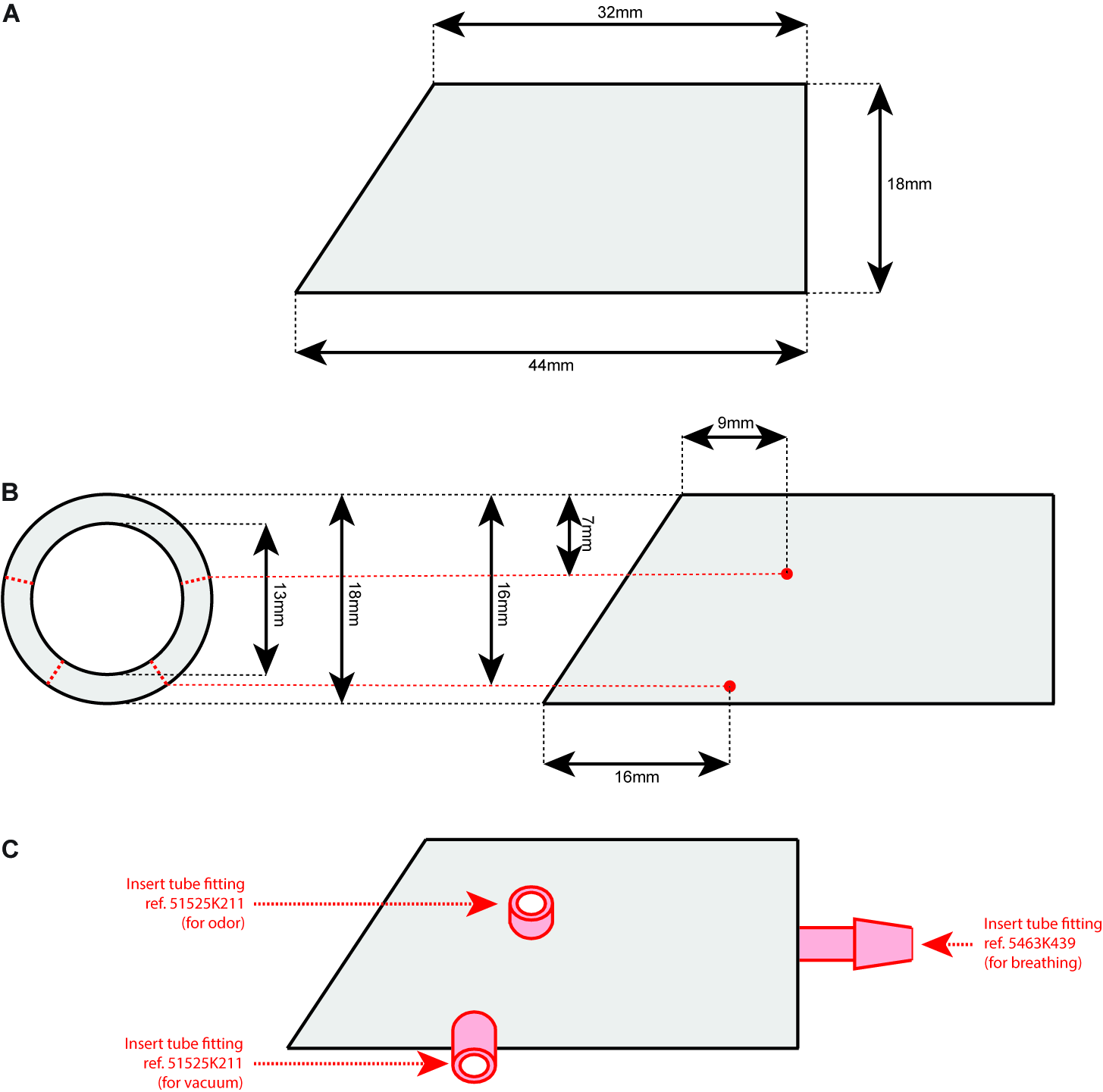

Supplement: Figure 1-2 — Instructions to Build an Odor Delivery Mask. Related to Figure 1. Below we explain how to build a mask, including all necessary measurements and materials. (A) Cut a piece of PVC tubing (outer diameter: 18 mm; inner diameter: 13 mm) as indicated on the diagram. PVC allows for a gentle yet firm seal around the mouse's face. (B) Using injection needles of gradually increasing diameters, puncture the piece of tubing at the positions indicated by the red marks on the diagram. When puncturing, insert the needle tangentially to the piece of tubing. Each hole should be less than 0.09in (2.2 mm) in diameter, to ensure that the tube fitting fits snugly. (C) Insert the nylon tube fittings as indicated on the diagram. For the odors and vacuums, use tube fittings ref. 51525K211 (McMaster-Carr, USA). For the breathing, use a tube fitting ref. 5463K439 (McMaster-Carr, USA), and cover the threads with teflon tape before insertion. Secure all tube fittings in position by applying a few drops of epoxy glue around each of them, on the outside of the mask. Let the mask rest for at least a week in a well-ventilated area, so as to eliminate any remaining epoxy fumes. Download Figure 1-2, TIF file. [file eneuro-11-ENEURO.0155-24.2024-s003.tif]

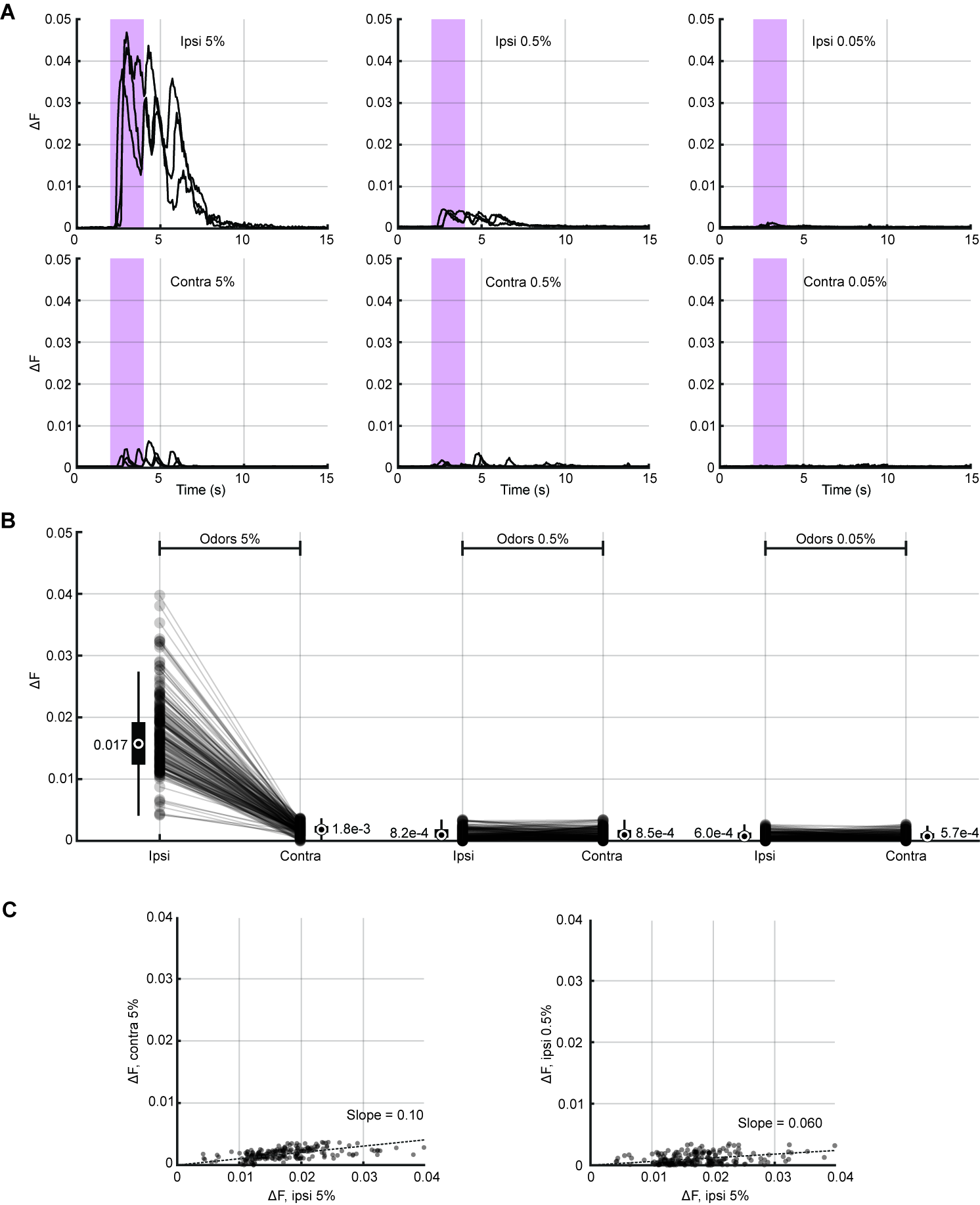

Supplement: Figure 1-3 — Glomerular Activity at Various Odor Concentrations. Related to Figure 1. (A) Activity of one exemplary glomerulus to unilateral presentations of ethyl tiglate at various concentrations. Purple: odor delivery. Each trace is one odor delivery (3 repeats per side, per concentration). (B) Glomerular activity. Each line represents the activity of one glomerulus to one odor, averaged over three repeats on each side (N = 52 glomeruli from 3 mice). Four odors were tested in total (ethyl tiglate, isopropyl tiglate, hexanal, allyl butyrate), each odor was tested at three different concentrations (5%, 0.5%, and 0.05% volume/volume in diethyl phthalate), with three repeats for each condition. All recordings were performed on awake mice. Responses are shown as “delta F” (mean fluorescence during the 2 s of odor presentation minus mean fluorescence over the two seconds before) rather than “delta F over F0” as some glomeruli showed no detectable activity before odor presentations. The distributions of glomerular responses are also shown as box plots (center dot: median; thick bar: quartiles; thin bar: 95% confidence interval). (C) Left: ipsi- versus contralateral responses at 5% concentration. Right: ipsilateral responses at 5% concentration versus ipsilateral responses at 0.5% concentration. Dotted line: linear regression (response on the y-axis = slope x response on the x-axis). The regression slope is given on each graph. Download Figure 1-3, TIF file. [file eneuro-11-ENEURO.0155-24.2024-s004.tif]

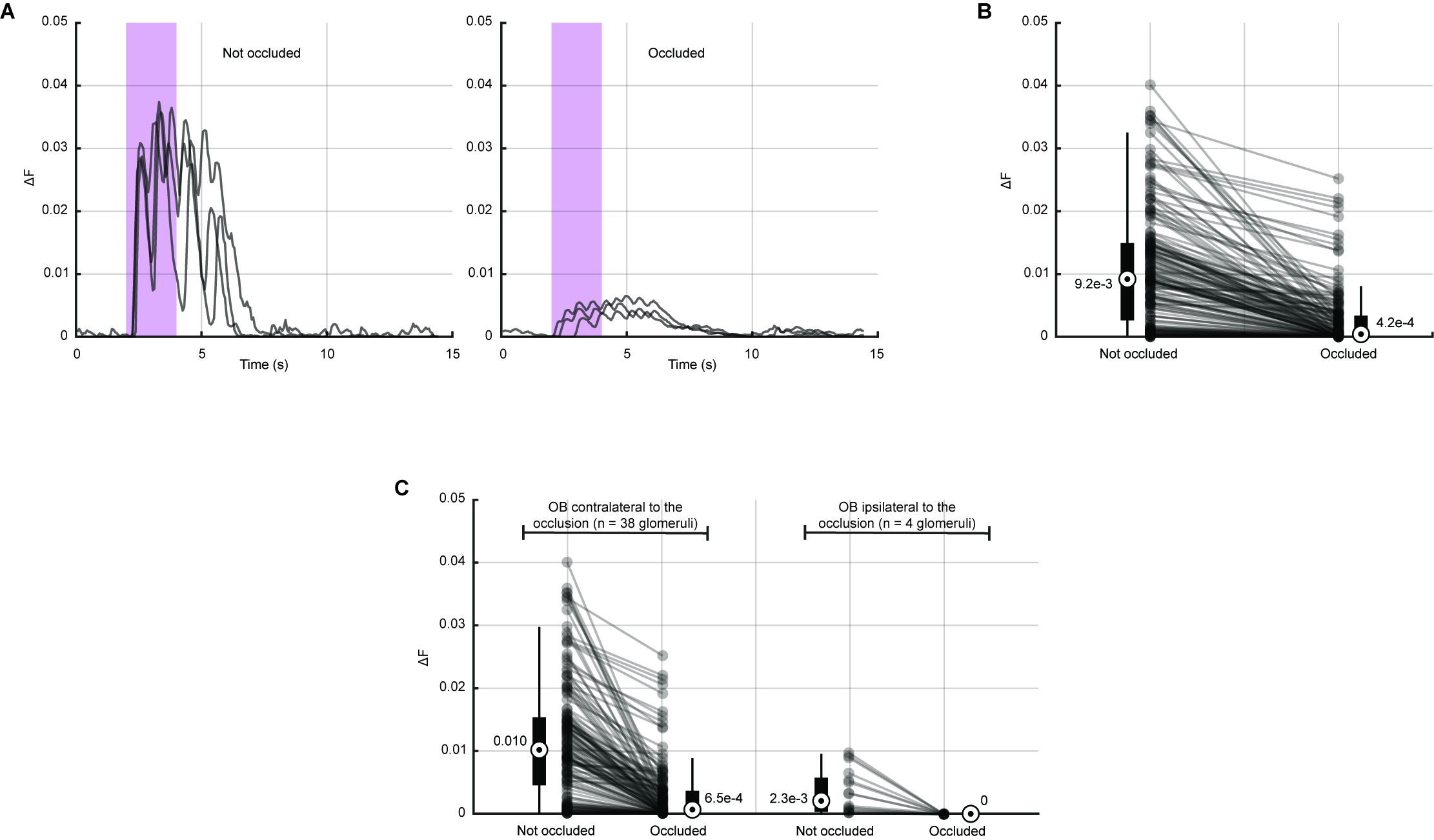

Supplement: Figure 1-4 — Glomerular Activity After Naris Occlusion. Related to Figure 1. (A) Activity of one exemplary glomerulus to unilateral presentations of ethyl tiglate, after the mouse's contralateral nostril has been occluded. Purple: odor delivery. Each trace is one odor delivery (3 repeats per side). (B) Glomerular activity after naris occlusion. Each line represents the activity of one glomerulus to one odor, averaged over three repeats on each side (N = 42 glomeruli from 4 imaging sessions - two mice were imaged once with their right nostril occluded, while a third mouse was imaged on two separate sessions, once with its right naris occluded and once with its left naris). While we blindly looked for glomeruli on both OB, only 4 glomeruli out of 42 were located on the OB ipsilateral to the occlusion. Four odors were tested in total, three repeats each (ethyl tiglate, isopropyl tiglate, hexanal, allyl butyrate; 5% volume/volume dilution in diethyl phtalate). All recordings were performed on awake mice, the day following the naris stitching. Responses are shown as “delta F” (mean fluorescence during the 2 s of odor presentation minus mean fluorescence over the two seconds before) rather than “delta F over F0” as some glomeruli showed no detectable activity before odor presentations. The distribution of glomerular responses to odors delivered to the not occluded and occluded nostrils are also shown as box plots (center dot: median; thick bar: quartiles; thin bar: 95% confidence interval). The median response to the non occluded nostril is 20.7 times greater than to the occluded nostril. (C) Same as (B), except we show the glomeruli from the OB contra- and ipsilateral to the naris occlusion separately. On the OB contralateral to the occlusion, the median response to the non occluded nostril is 15.4 times greater than to the occluded nostril. Download Figure 1-4, TIF file. [file eneuro-11-ENEURO.0155-24.2024-s005.tif]

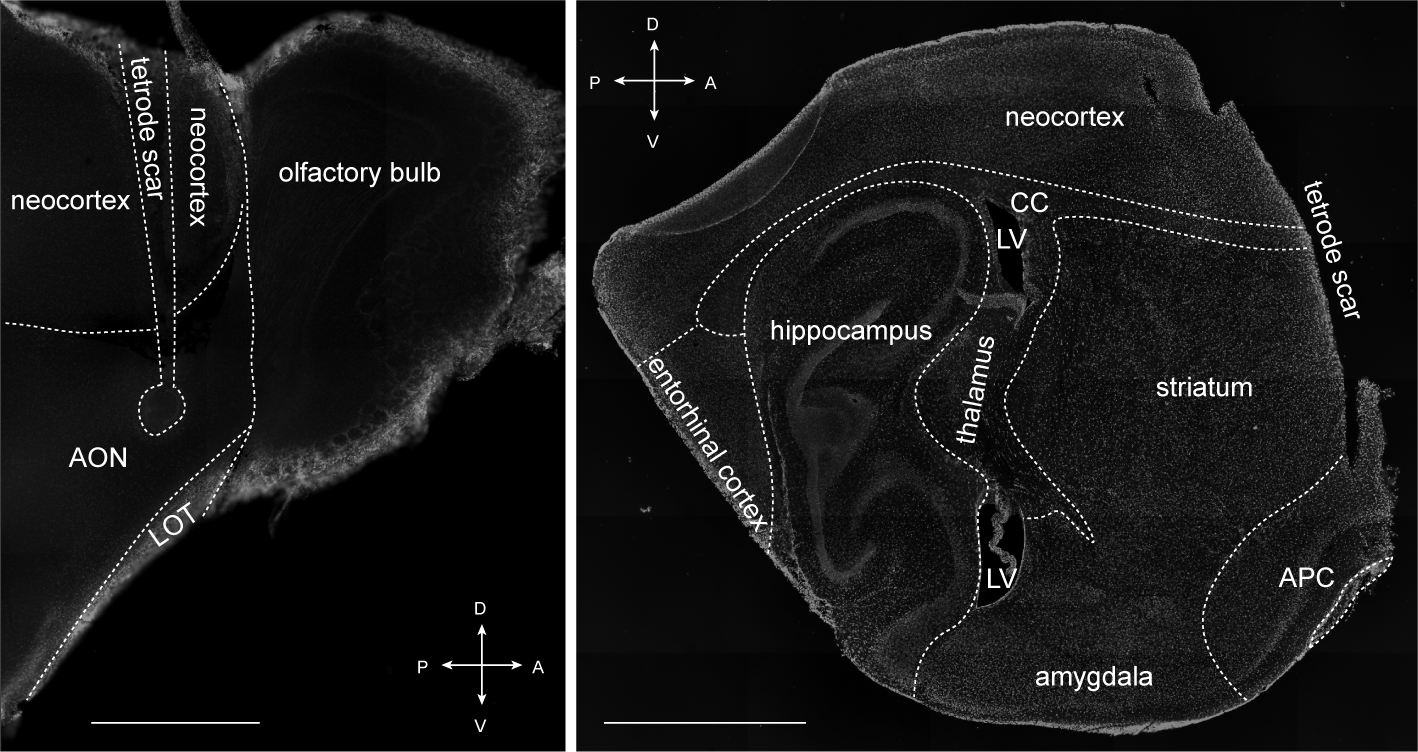

Supplement: Figure 2-1 — Post-Mortem Confirmation of Tetrode Placement in the Olfactory Cortex. Related to Figure 2. Sagittal sections of two exemplar animals, implanted in the AON (left) and APC (right). The round shape on the ventral end of the tetrode scar in the left panel was caused by post-mortem electrolesion. For animals recorded in the APC, the tetrodes went all the way through the brain, until they reached the ventral part of the skull. As a consequence, it was not possible to save the most anterior part of the section in the right panel. A: anterior. P: posterior. D: dorsal. V: ventral. CC: corpus callosum. LV: lateral ventricle. LOT: lateral olfactory tract. Scale bar: 0.25 mm (left) or 1 mm (center, right). DAPI staining. We did not trace the border between striatum and amygdala as we could not confidently determine it. Download Figure 2-1, TIF file. [file eneuro-11-ENEURO.0155-24.2024-s006.tif]

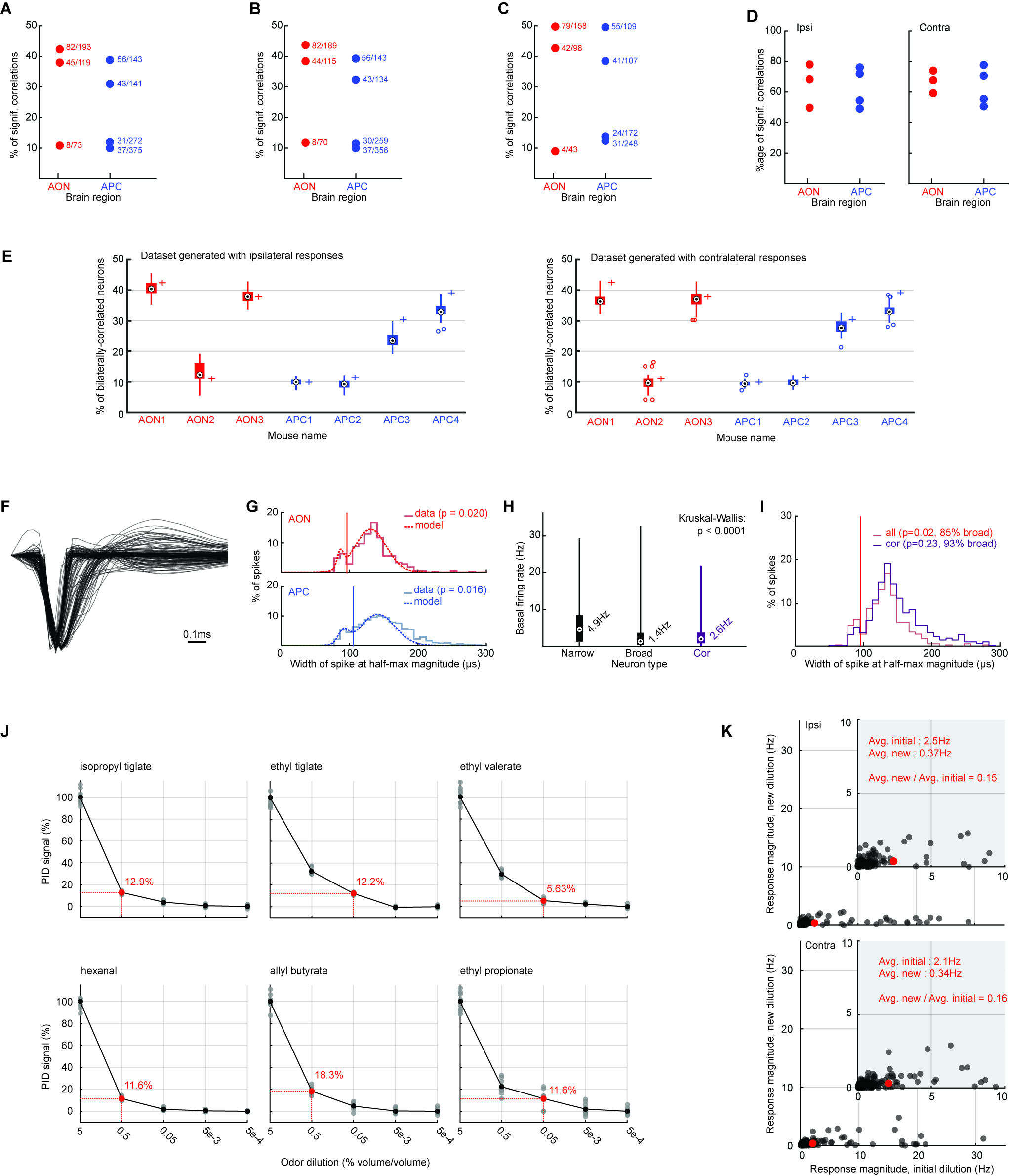

Supplement: Figure 3-1 — Bilaterally-Correlated Neurons. Related to Figure 3. (A) to (D) Percentage of bilaterally-correlated neurons per mouse. (A) Identical to Figure 3B. (B) Same as Figure 3B, except we kept only the neurons responding to at least one odor on one side for this analysis. (C) Same as Figure 3B, except we kept only the neurons responding to at least one odor on each side for this analysis (note that the odors eliciting responses can be different on each side). (D) Correlation across artificially-generated trials. For each mouse, each side, each neuron, each odor, two sets of 7 artificial trials were generated using a Poisson process. Then for each side, an analysis similar to Figure 3B was performed, except we looked for correlations between the first and second sets of 7 artificial trials, instead of correlations between ipsi and contralateral trials. A full circle means the percentage is higher than chance (significance is determined with a bootstrapping method similar to Figure 3C). Panel (D) allows us to estimate the apparent percentage of bilaterally-correlated neurons one would expect if all neurons were bilaterally-correlated. (E) Upper estimates of bilateral correlations in ideal conditions. For each mouse, each side, each neuron, we randomly split the 7 trials into two groups of 3 and 4, to calculate the correlation between the two groups of trials. We then computed the percentage of bilateral correlations among all neurons. We repeated this process for all the possibilities of trial splits. The + signs to the right of each distribution show the actual values from Figure 3B for comparison. The goal of this test is to estimate an upper bound for the percentage of bilateral correlations one could expect in each mouse. (F) Spike traces from mouse PPC1. The average spike of all the neurons recorded from APC1 are shown. The spike amplitudes were normalized for display. Note the apparent bimodal distribution of spike widths. (G) Histograms of the spike widths at half-max [file eneuro-11-ENEURO.0155-24.2024-s007.tif]

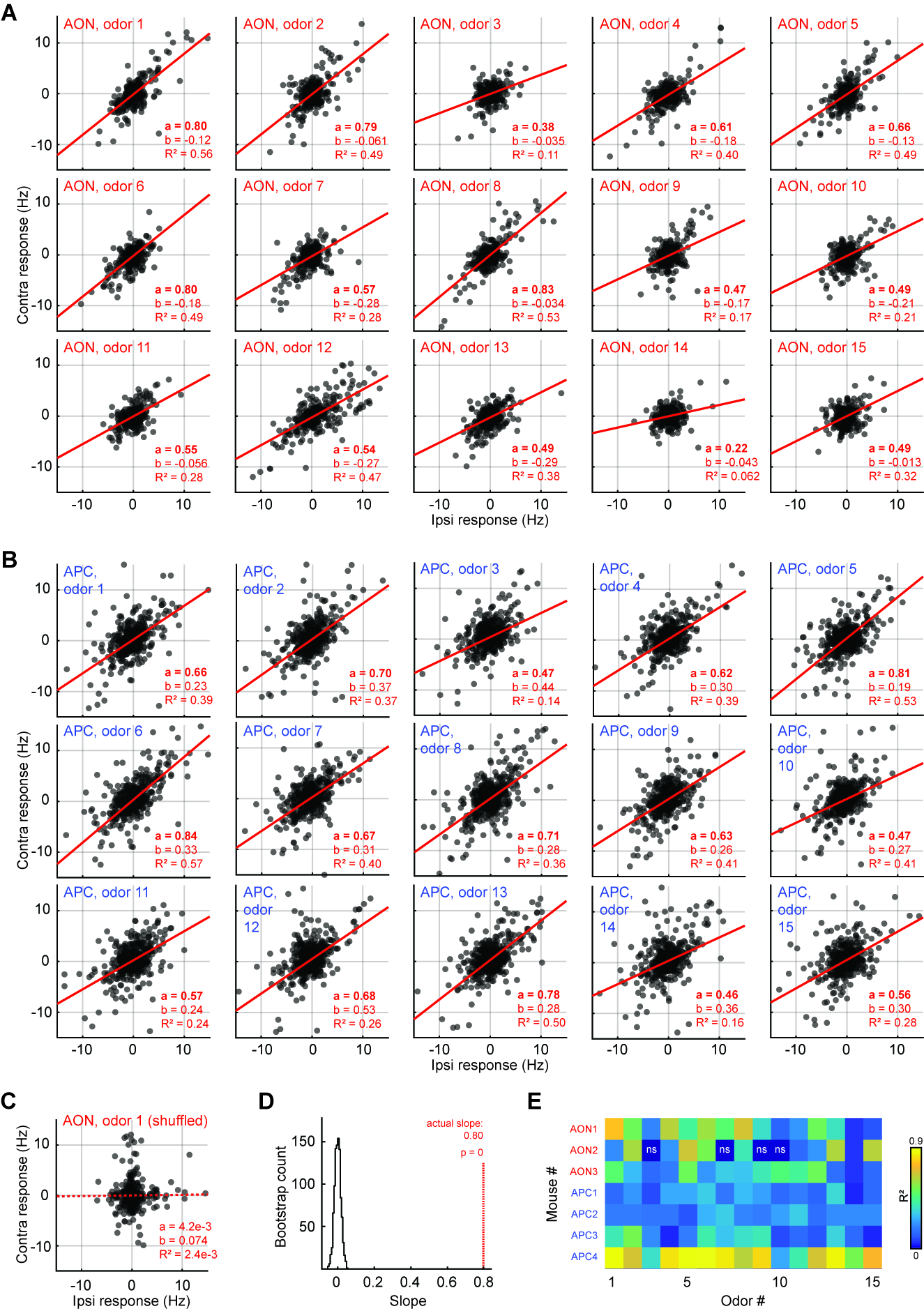

Supplement: Figure 3-2 — Correlation of Population Odor Representations. Related to Figure 3. (A) and (B) Ipsi- versus contralateral responses per odor in both OC regions tested. (A) AON. (B) APC. Red line: linear regression (contra = a x ipsi + b). Solid lines show significant regressions (F-test, p < 5%). Odor # is the same as Figure 1-1A. Each dot is one neuron. The top left graph for each region is also shown in Figure 3G. (C) Same as panel (A), odor 1, except neuron identity has been randomly shuffled on each side. Note the absence of significant correlation. (D) Significance of the regression slope for panel (A), odor 1. We built a chance distribution by repeating the shuffling shown in panel (C) 1000 times. We then compared the actual value of the slope to this distribution to determine its significance. Here we show the distribution of chance slopes for the AON, odor 1. The results of our bootstrap strategy matched the results of the F-test mentioned above. (E) We performed the same analysis as panels (A) and (B), but we looked at each mouse separately. Here we report the R2 value of each regression, as well as whether the correlation was significant (F-test). N.s. means that the correlation was not statistically significant (p ≥ 5%). Download Figure 3-2, TIF file. [file eneuro-11-ENEURO.0155-24.2024-s008.tif]

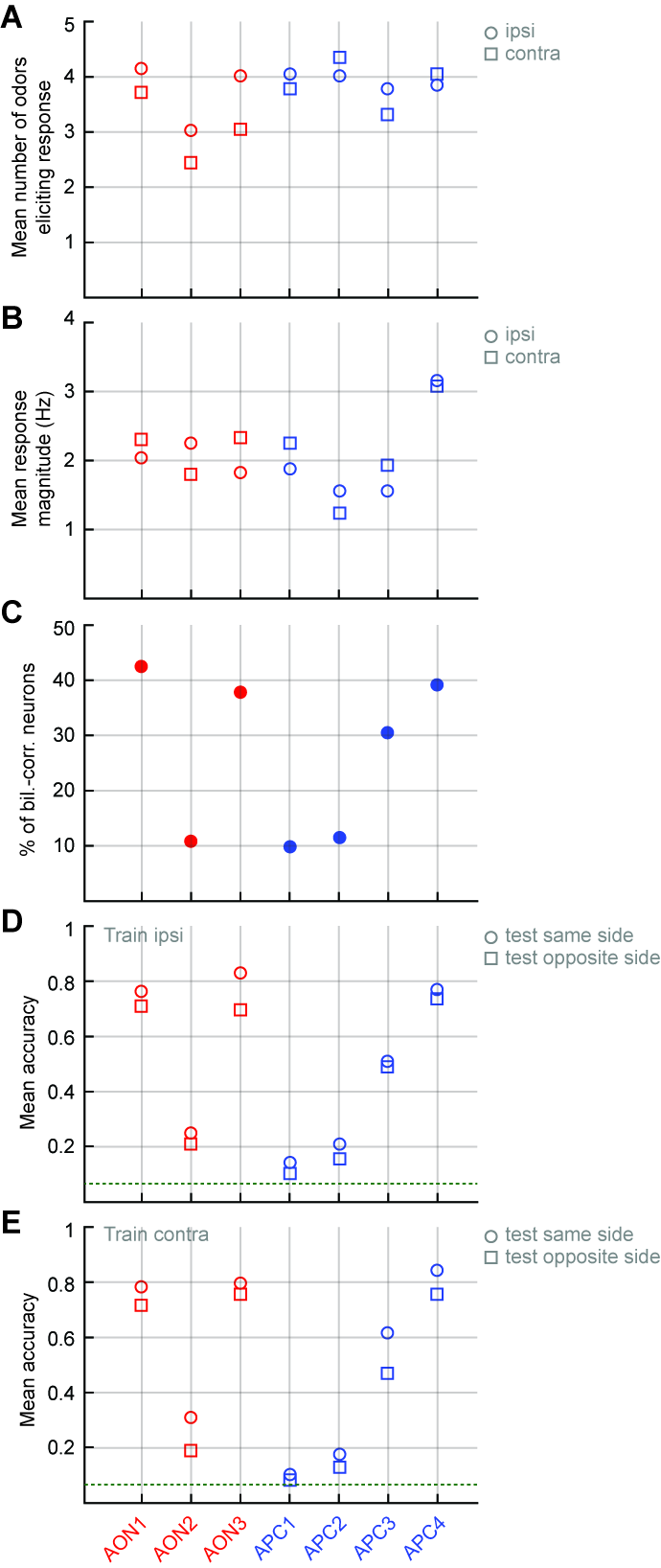

Supplement: Figure 3-3 — Variability Across Mice. Related to Figures 3 and 4. In this figure we point at factors that may explain the variability we observed across mice in terms of percentage of bilaterally-correlated neurons, as well as odor decoding accuracy. (A) Mean number of odors eliciting response (related to Figure 2E). (B) Mean response magnitude (related to Figure 2F). (C) Percentage of bilaterally-correlated neurons (related to Figure 3B). Solid dot: significant percentage. (D) Mean accuracy of the odor decoding process with model trained on responses to ipsilateral presentations (related to Figure 4-1B). (E) Similar to (D) with training on contralateral presentations. Download Figure 3-3, TIF file. [file eneuro-11-ENEURO.0155-24.2024-s009.tif]

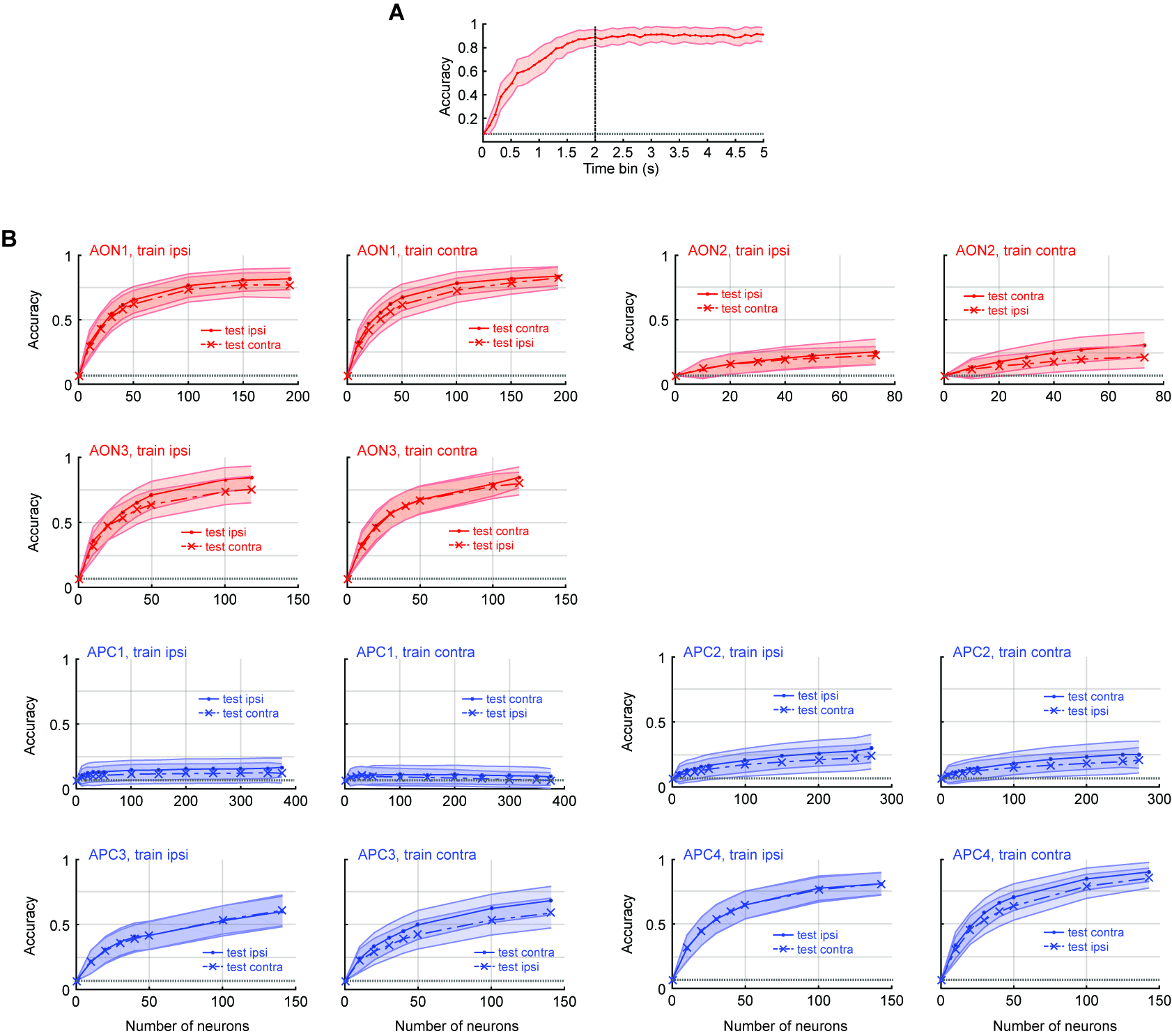

Supplement: Figure 4-1 — Ipsi- and Contralateral Odor Decoding. Related to Figure 4. (A) Decoding accuracy with varying odor response time windows. For each data point, response vectors were built by summing spikes from 0 s to the time indicated on the x-axis (responses aligned to the first sniff after odor onset, see Methods for details). This example shows the odor decoding for all neurons in the AON, train ipsi, test ipsi. Odor decoding accuracy is maximal at 2 s, therefore we decided to perform all our decoding analysis over an odor response window of 2 s. (B) Same as Figures 4B-4C, except the decoding process was applied to each mouse separately. Accuracy at n = 50 neurons is significantly higher than chance for all mice. The mice with poorer decoding accuracy were usually the ones in which neurons showed fewer / weaker responses on average, as well as fewer bilaterally-correlated neurons (see Figure 3-3). Download Figure 4-1, TIF file. [file eneuro-11-ENEURO.0155-24.2024-s010.tif]

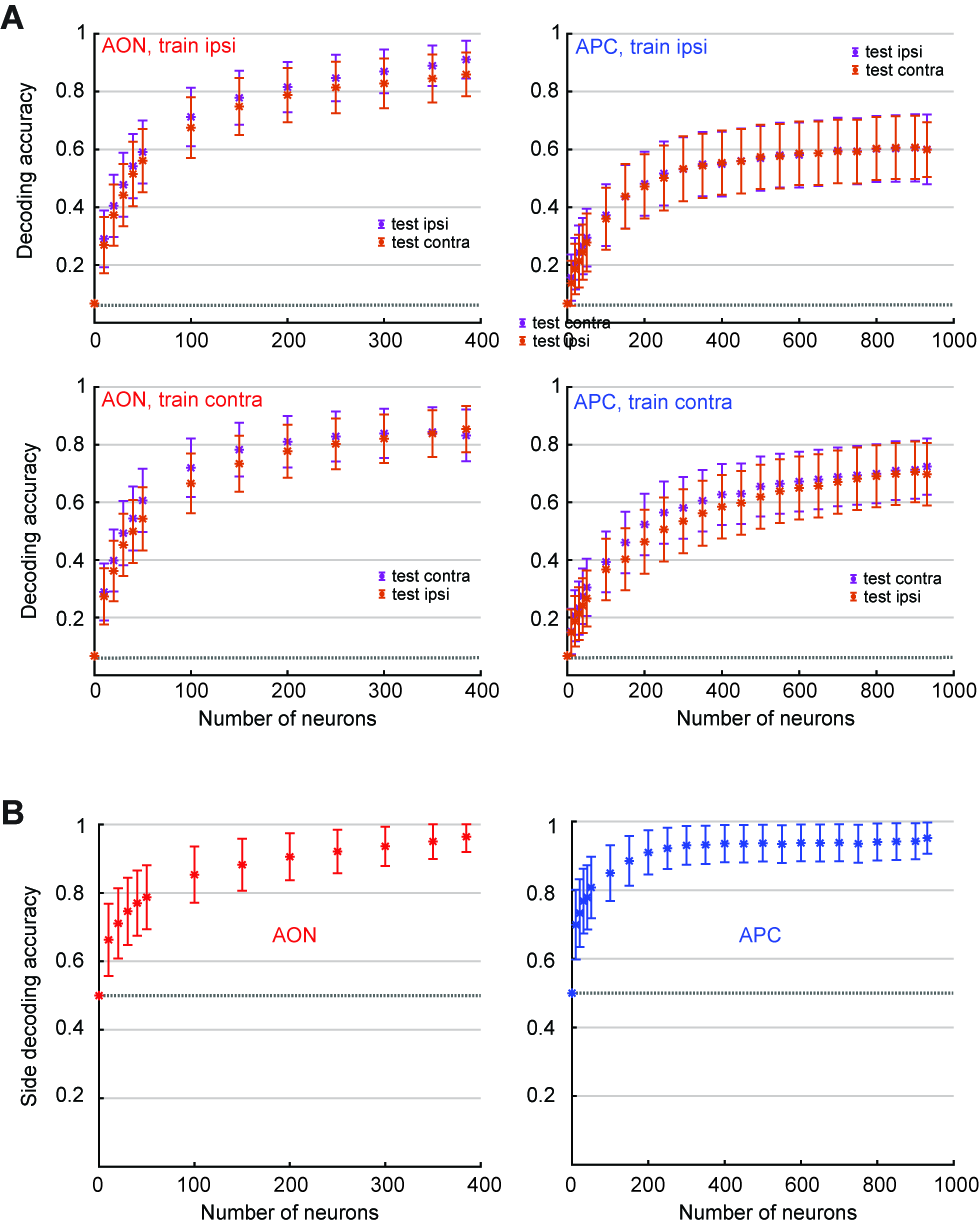

Supplement: Figure 4-2 — Ipsi- and Contralateral Odor Decoding with No Blank Subtraction. Related to Figure 4. We replicate the decoding results from Figure 4B-D, using a new metric to compute odor responses with no blank trial subtraction. More precisely, we performed decoding on odor responses R calculated using the following formula: R=∑t=17(Durot−Befot)/7 (see Methods for the significance of each symbol). The goal of this analysis is to verify that the way we compute odor responses does not influence the decoder’s results. (A) Similar to Figure 4B-C (odor decoding). (B) Similar to Figure 4D (side decoding). Download Figure 4-2, TIF file. [file eneuro-11-ENEURO.0155-24.2024-s011.tif]

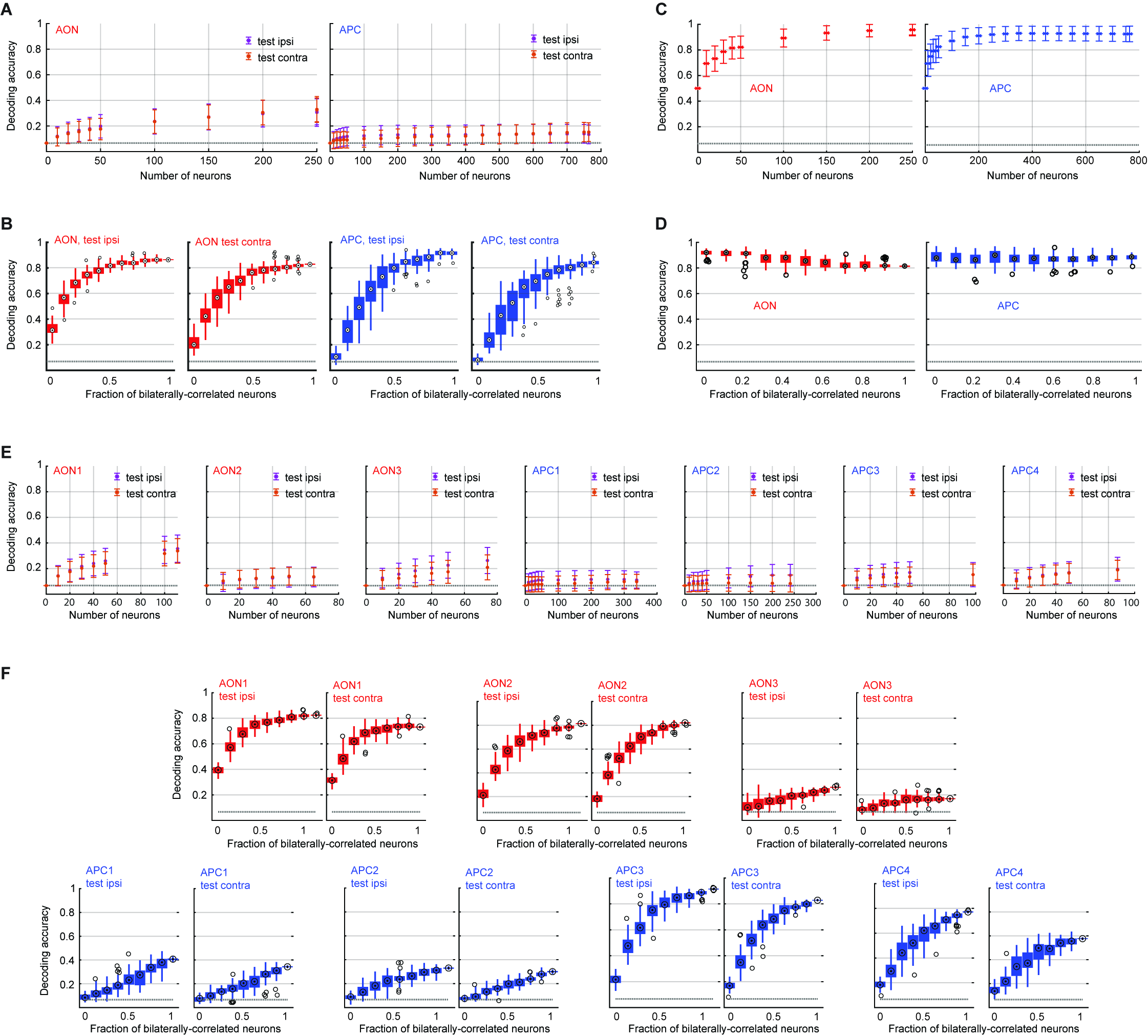

Supplement: Figure 4-3 — Bilaterally-Correlated Neurons and Decoding Performance. Related to Figure 4. (A) Same as Figure 4B, except we removed all the bilaterally-correlated neurons before computing the accuracies. (B) For each decoding, we randomly took 135 neurons, including a certain fraction of bilaterally-correlated neurons (as indicated on the y-axis). For each region, each fraction of bilaterally-correlated neurons, each test side, we repeated this decoding 50 times, each time with a new set of randomly-picked neurons. The box plots show the median (white and black dot), 1st and 3rd quartiles (thick bar), 95% confidence interval (thin bar), and the outliers (black circles). All training data used for this panel are from the ipsilateral side. (C) Same as panel (A), for side decoding instead of odor decoding. (D) Same as panel (B), for side decoding instead of odor decoding. (E) Same as panel (A), except the analysis was performed on each individual mouse. (F) Same as panel (B), except the analysis was performed on each individual mouse. Here, for each decoding, instead of 135 neurons, we took N neurons, N being the total number of bilaterally-correlated found in each mouse. For all panels, the gray dotted line shows chance level. Download Figure 4-3, TIF file. [file eneuro-11-ENEURO.0155-24.2024-s012.tif]

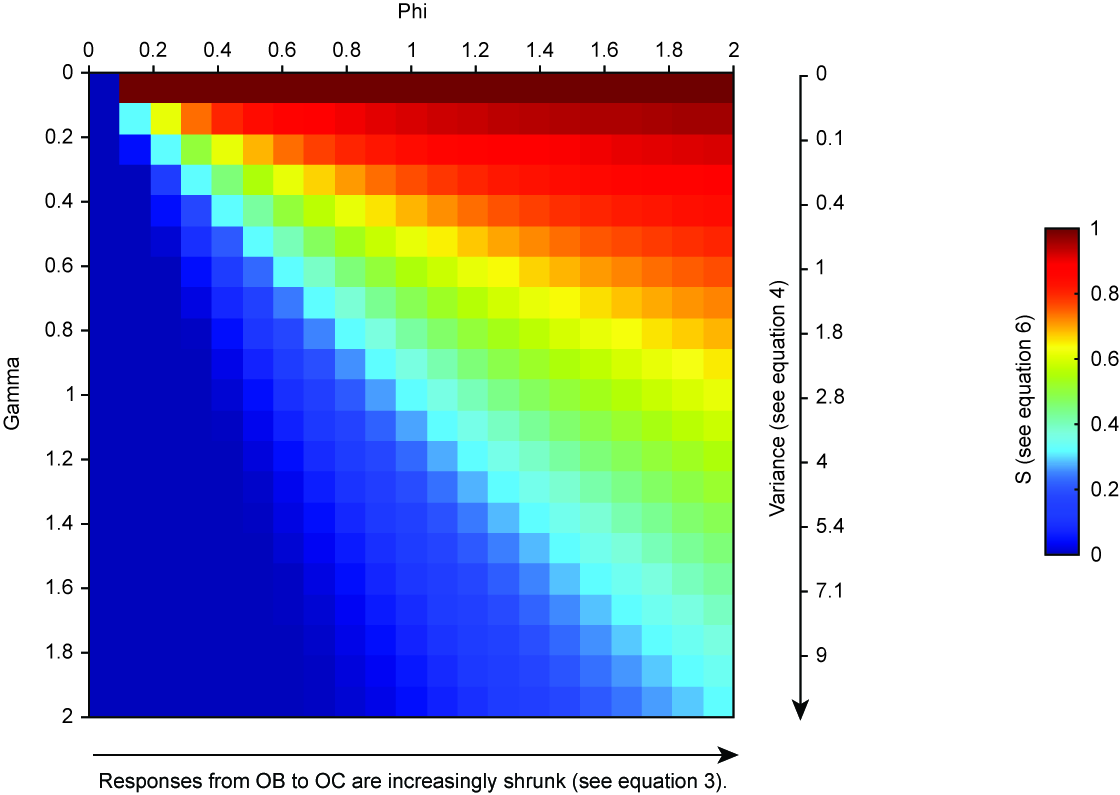

Supplement: Figure 5-1 — Visualization of the Relationship Between Phi and Gamma. Related to Figure 5. This figure shows a heatmap representation of the relationship between gamma, phi, and S, as shown in equation 7. In addition, we provide variance equivalents to the gamma values, for an exemplar data set with the following characteristics: 50 glomeruli, 15% chance that a given neuron-odor pair is significantly responding, magnitude of average odor response 1.6 Hz. Download Figure 5-1, TIF file. [file eneuro-11-ENEURO.0155-24.2024-s013.tif]

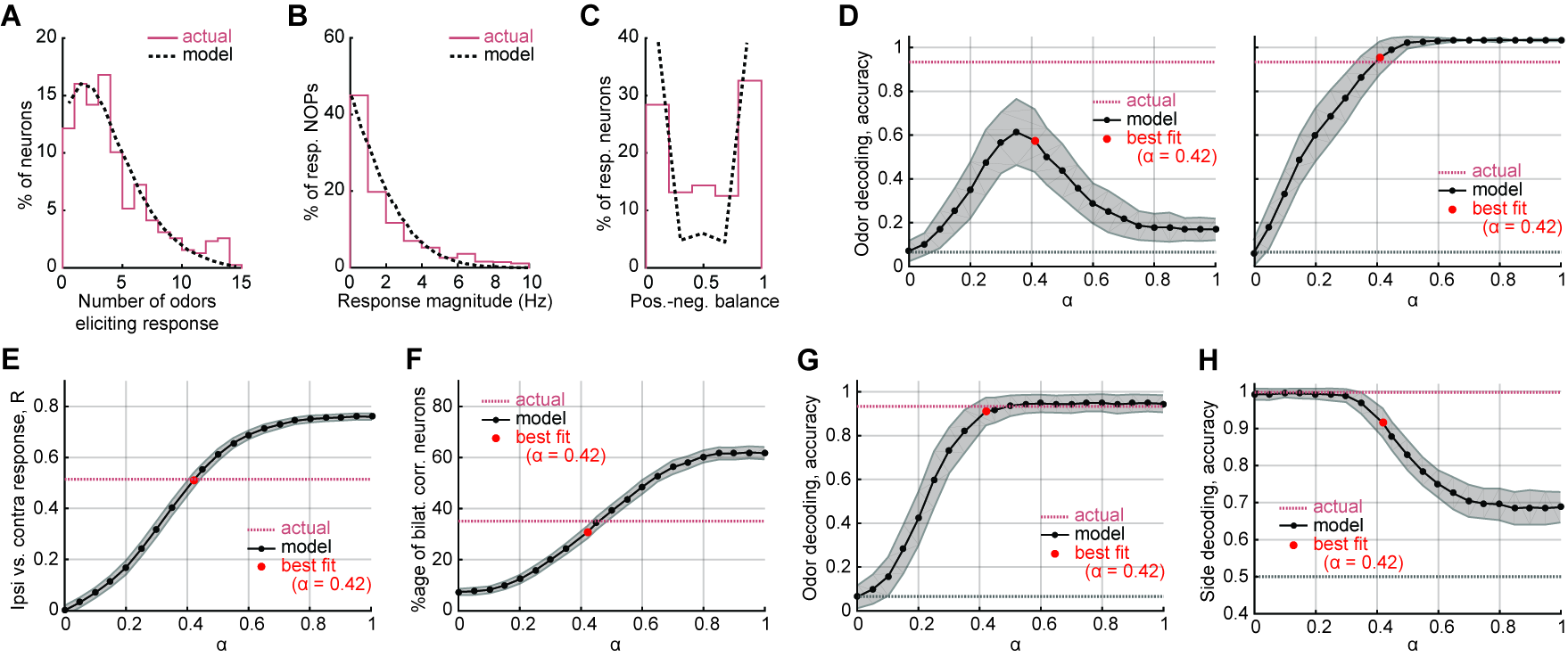

Supplement: Figure 6-1 — Modeling Olfactory Cross Cortical Connections. Related to Figure 6. (A) to (C) Same as Figures 6B-6D, except for we are comparing the distributions of mouse ipsilateral data and our simulations of ipsilateral responses with optimal alpha (0.42). Mouse data is identical to the contralateral lines from Figures 2E-2F, top panels. (D) Same as Figure 6I, except we decoded ipsilateral odors using the contralateral presentations. (Left) Using closest Euclidean neighbor decoding is markedly worse in simulations than mice, due mainly to the difference in magnitude of responses that results as you increase the structure parameter. (Right) Using closest angular neighbors instead recaptures better performance, and the best alpha overlaps with the AON performance. This suggests that, in the left panel, the decoder limited performance rather than lack of information. (E) to (H) Same as Figures 6F, 6G, 6I, and 6J respectively, except each OC layer contains 25,000 neurons (instead of 50,000 neurons in Figure 6, see Methods). These show that the optimal alpha level (0.42) is robust to changes in the number of cortical neurons simulated. Download Figure 6-1, TIF file. [file eneuro-11-ENEURO.0155-24.2024-s014.tif]

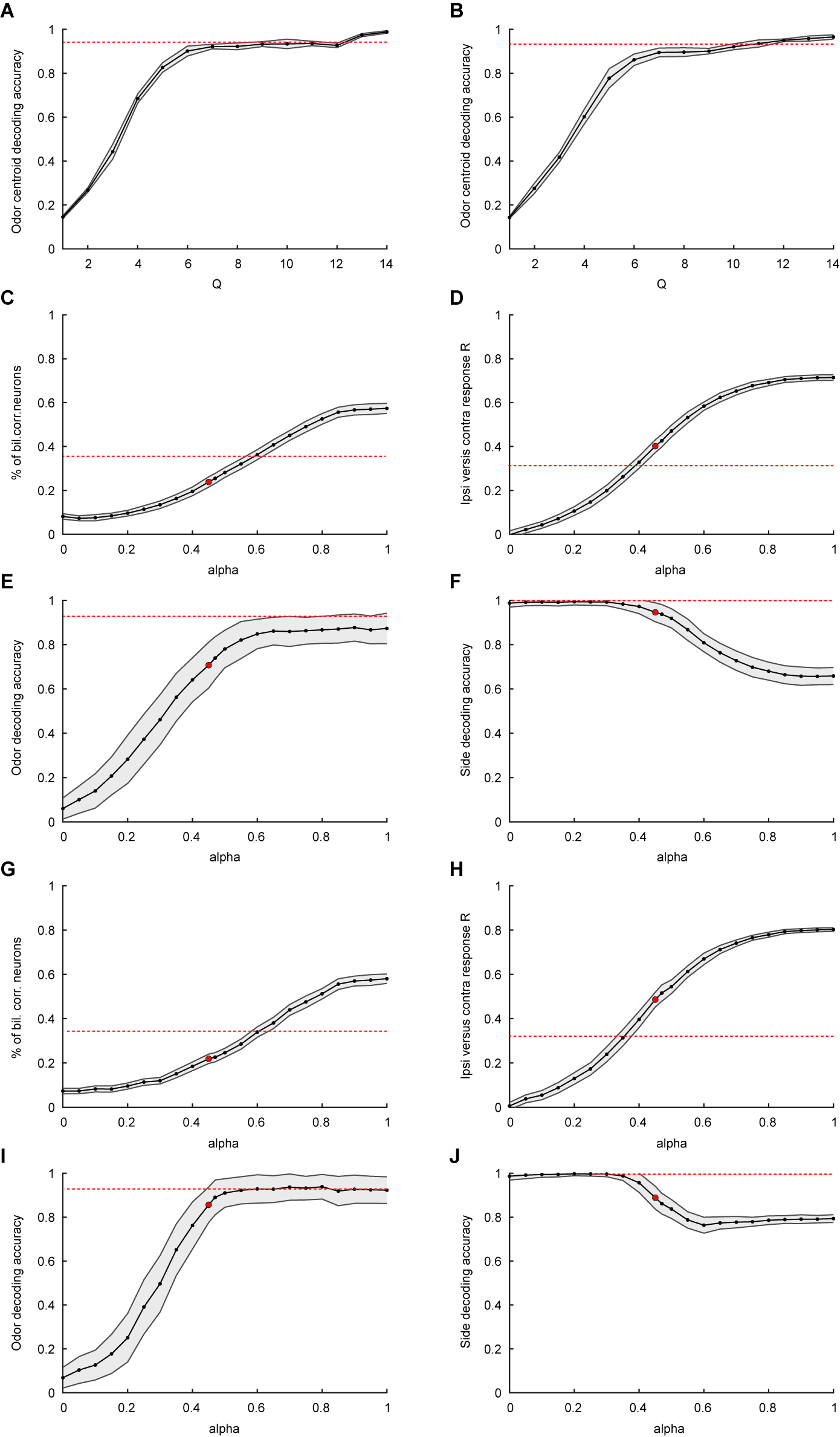

Supplement: Figure 6-2 — Low-dimensional structured connectivity is sufficient to produce alignment. Related to Figure 6. (A) and (B) Odor decoding accuracy using 385 neurons as Q is varied, averaged over 200 iterations of decoding and 10 different creations of G_Struct, (A) approximates using SVD and (B) using PCA. (C) – (F) are the same as Figure 6F, G, I, and J but using an approximate G_Struct created with 7 singular values. (G) – (J) are the same but using 6 PCA components. Download Figure 6-2, TIF file. [file eneuro-11-ENEURO.0155-24.2024-s015.tif]
