# Supplementary material for: Bilateral Alignment of Receptive Fields in the Olfactory Cortex
Source: eNeuro. 2024 Nov 5;11(11):ENEURO.0155-24.2024. doi: 10.1523/ENEURO.0155-24.2024 (PMC11540595; doi:10.1523/ENEURO.0155-24.2024)
Supplement: Table 1-1 — Mice Recorded in this Study. All tetrode recordings were performed in awake, head-restrained mice. Download Table 1-1, DOCX file. [file eneuro-11-ENEURO.0155-24.2024-s016.docx]

**Extended Data Table 1-1: Mice Recorded in this Study**. All tetrode recordings were performed in awake, head-restrained mice.

| Brain region | Mouse name | Age at the first recording | Number of sessions | Number of neurons |
| --- | --- | --- | --- | --- |
| AON | AON1 | 3 m.o. | 11 | 193 |
|  | AON2 | 3 m.o. | 17 | 73 |
|  | AON3 | 3 m.o. | 14 | 119 |
| APC | APC1 | 3 m.o. | 15 | 375 |
|  | APC2 | 4 m.o. | 12 | 272 |
|  | APC3 | 3 m.o. | 11 | 141 |
|  | APC4 | 3 m.o. | 11 | 143 |
| OB | OB1 | 7 m.o. | 1 | 7 |
|  | OB2 | 7 m.o. | 1 | 9 |
|  | OB3 | 8 m.o. | 1 | 11 |
|  | OB4 | 8 m.o. | 2 | 15 |
