# Supplementary material for: Bilateral Alignment of Receptive Fields in the Olfactory Cortex
Source: eNeuro. 2024 Nov 5;11(11):ENEURO.0155-24.2024. doi: 10.1523/ENEURO.0155-24.2024 (PMC11540595; doi:10.1523/ENEURO.0155-24.2024)
Supplement: Table 1-2 — Statistical Tests Performed in this Study. The statistical tests showing significant differences are in red (critical value: 5%). For post-hoc tests, the p-values reported in the table have been corrected for multiple testing problem (Bonferroni method). Download Table 1-2, DOCX file. [file eneuro-11-ENEURO.0155-24.2024-s017.docx]

**Extended Data Table 1-2:** **Statistical Tests Performed in this Study**. The statistical tests showing significant differences are in red (critical value: 5%). For post-hoc tests, the p-values reported in the table have been corrected for multiple testing problem (Bonferroni method).

| Figure | Test | P-value | Post-hoc test | Post-hoc p-values |
| --- | --- | --- | --- | --- |
| Figure 1F, histogram | Z-test for the null hypothesis that the contralateral responses come from a Gaussian distribution (μ = 0, σ = 0.56). | p = 0.84 | N/A | N/A |
|  |  |  |  |  |
| Figure 2B | Wilcoxon rank-sum test, AON versus APC | p < 1.0e-4 | N/A | N/A |
| Figure 2E | Wikcoxon signed-rank test, ipsi versus contra, per region | AON, p = 9.4e-3 | N/A | N/A |
|  |  | APC, p = 0.51 |  |  |
| Figure 2E | Wilcoxon rank-sum test, AON versus APC | "ipsi" distributions, p = 0.55 | N/A | N/A |
|  |  | "contra" distributions, p = 0.68 | N/A | N/A |
| Figure 2F | Wilcoxon signed-rank test, ipsi versus contra, per region | AON, p = 8.2e-3 | N/A | N/A |
|  |  | APC, p = 0.94 |  |  |
| Figure 2F | Wilcoxon rank-sum test, AON versus APC | "ipsi" distributions, p < 1.0e-4 | N/A | N/A |
|  |  | "contra" distributions, p < 1.0e-4 | N/A | N/A |
| Figure 2G | Wilcoxon rank-sum test, ipsi versus contra, per region | AON, p = 0.99 | N/A | N/A |
|  |  | APC, p = 0.41 |  |  |
| Figure 2G | Wilcoxon rank-sum test, AON versus APC | "ipsi" distributions, p = 0.10 | N/A | N/A |
|  |  | "contra" distributions, p = 0.22 |  |  |
| Figure 3A | F-test, significance of the linear model | Top row, from left to right: | N/A | N/A |
|  |  | p = 0.41; p = 0.84; p = 0.54; p = 0.59 |  |  |
|  |  | Bottom row, from left to right: |  |  |
|  |  | p = 5.3e-6; p = 5.5e-4; p = 6.6e-3; p = 2.2e-4 |  |  |
| Figure 3D | Wilcoxon rank-sum test, AON versus APC | p = 0.013 | N/A | N/A |
| Figure 3E | Wilcoxon rank-sum test, AON versus APC | p = 0.25 | N/A | N/A |
| Figure 3F | Wilcoxon rank-sum test, AON versus APC | p = 3.9e-3 | N/A | N/A |
| Figure 3G | F-test, significance of the linear model | AON, p < 1.0e-4 | N/A | N/A |
|  |  | APC, p < 1.0e-4 |  |  |
| Figures 4B and 4C | Wikcoxon signed-rank test, distribution of accuracies obtained at n = 350 neurons versus chance accucacy (1/15). | p < 1.0e-4 for all regions, all conditions | N/A | N/A |
|  |  |  |  |  |
| Figure 4D | Wikcoxon signed-rank test, distribution of accuracies obtained at n = 350 neurons versus chance accucacy (1/2). | p < 1.0e-4 for all regions | N/A | N/A |
|  |  |  |  |  |
| Figure 1-1E top panel | Wilcoxon rank-sum test between the two distributions | p = 0.25 | N/A | N/A |
| Figure 1-1E bottom panel | Wilcoxon rank-sum test between the two distributions | p = 0.62 | N/A | N/A |
| Figures 3-1F and 3-1H | Hartigan dip test of unimodality | AON, p = 0.020 | N/A | N/A |
|  |  | APC, p = 0.016 |  |  |
|  |  | AON bilat-corr neurons, p = 0.23 |  |  |
| Figure 3-1G | Kruskal-Wallis test across neuron types (narrow, broad, bilatetally-correlated) | p < 1.0e-4 | Wilcoxon rank-sum test | narrow vs broad, p < 1.0e-4 |
|  |  |  |  | narrow vs bilat-corr, p < 1.0e-4 |
|  |  |  |  | broad vs bilat-corr, p < 1.0e-4 |
| Figures 3-2A and 3-2B | F-test, significance of the linear model | p < 1.0e-4 for regions, all odors | N/A | N/A |
| Figure 3-2C | F-test, significance of the linear model | p = 0.42 | N/A | N/A |
| Figure 4-1B | Wikcoxon signed-rank test, distribution of accuracies obtained at n = 50 neurons versus chance accucacy (1/15). | AON, p < 1.0e-4 for all mice, all conditions | N/A | N/A |
|  |  | APC, p < 1.0e-4 for all mice, all conditions |  |  |

## Extended Data Note 5-1: Random Connections Produce Zero Correlations – a Derivation.

We will prove that if the same response is mapped through two different random matrices the resulting representations will be uncorrelated. Let $\mathbf{x}$ be our $N$-dimensional input pattern, for example the bulb response to an odor. Project it through two different random matrices to the same set of cortical neurons. The two matrices, $\mathbf{J}$and $\mathbf{G}$, each have independently identically distributed elements drawn from a mean zero distribution with finite variance. This creates two further representations which we shall later pass through an activation function. First, however, we shall consider the pre-thresholded representations:

|  | $h_{i}=\sum_{j=1}^{N} J_{ij}x_{j}$ and $g_{i}=\sum_{j=1}^{N} G_{ij}x_{j}$. | (17) |
| --- | --- | --- |

We can use the Multivariate Central Limit Theorem to argue that these two representations are jointly normally distributed with zero mean:

|  | $\left( \begin{matrix} \begin{matrix} h_{i} \end{matrix} \\ g_{i} \end{matrix} \right)=\sum_{j=1}^{N} \left( \begin{matrix} \begin{matrix} J_{ij}x_{j} \\ G_{ij}x_{j} \end{matrix} \end{matrix} \right)\sim\mathcal{N}\left( 0,\Sigma\right)$ | (18) |
| --- | --- | --- |

The correlation matrix can be derived and shown to be diagonal:

|  | $\Sigma_{hh}=\mathbb{E}_{i}\left[ \sum_{j} J_{ij}x_{j}\sum_{k} J_{ik}x_{k} \right]\neq0$  $\Sigma_{\mathrm{hg}}=\mathbb{E}_{i}\left[ \sum_{j} J_{\mathrm{ij}}x_{j}\sum_{k} G_{\mathrm{ik}}x_{k} \right]=0$ | (19) |
| --- | --- | --- |

where the last equality follows from the independent, zero mean, nature of elements of **G** and **J**.

Now, since $h_{i}$ and $g_{i}$ are jointly normally distributed random variables with a diagonal covariance matrix, they are also independent. We then pass these representations through a nonlinearity. Using the fact that functions of independent variables are independent, the resulting output representations are also independent and hence uncorrelated.

This demonstration can be extended to many cases of direct interest. If we let $h$ and $g$ depend on different odors they are still independent and uncorrelated by a simple generalization of the previous argument:

|  | $h_{i}=\sum_{j} J_{ij}x_{1,j}$ and $g_{i}=\sum_{j} G_{ij}x_{2,j}$. | (20) |
| --- | --- | --- |

The experiments consider another similar case. Interpret h (g) as the unthresholded representation in the ipsilateral (contralateral) AON to an odor presented ipsilaterally (contralaterally). If the cross cortical matrix is random then thresholding these responses and mapping them cross cortically will not change their independence from one and other.

Previous work has shown that, if two correlated representations are projected through a random matrix, the resulting representations remain correlated (Babadi and Sompolinsky, 2014; Schaffer et al., 2018). The key difference in our work is that we are considering two different random matrices, one in each hemisphere. Hence, this derivation has shown that projecting a pair of correlated representations through two different random matrices eliminates the correlations between resulting outputs.

Therefore, since observed correlations between odors presented ipsilaterally and contralaterally are not zero, the cross cortical connectivity must be structured in some way.
