# Supplementary material for: Bilateral Alignment of Receptive Fields in the Olfactory Cortex
Source: eNeuro. 2024 Nov 5;11(11):ENEURO.0155-24.2024. doi: 10.1523/ENEURO.0155-24.2024 (PMC11540595; doi:10.1523/ENEURO.0155-24.2024)
Supplement: Extended Data Note 5-1 — Random Connections Produce Zero Correlations – a Derivation. Download Extended Data Note 5-1, DOCX file. [file eneuro-11-ENEURO.0155-24.2024-s018.docx]

## Extended Data Note 5-1: Random Connections Produce Zero Correlations – a Derivation.

We will prove that if the same response is mapped through two different random matrices the resulting representations will be uncorrelated. Let $\mathbf{x}$ be our $N$-dimensional input pattern, for example the bulb response to an odor. Project it through two different random matrices to the same set of cortical neurons. The two matrices, $\mathbf{J}$and $\mathbf{G}$, each have independently identically distributed elements drawn from a mean zero distribution with finite variance. This creates two further representations which we shall later pass through an activation function. First, however, we shall consider the pre-thresholded representations:

|  | $h_{i}=\sum_{j=1}^{N} J_{ij}x_{j}$ and $g_{i}=\sum_{j=1}^{N} G_{ij}x_{j}$. | (17) |
| --- | --- | --- |

We can use the Multivariate Central Limit Theorem to argue that these two representations are jointly normally distributed with zero mean:

|  | $\left( \begin{matrix} \begin{matrix} h_{i} \end{matrix} \\ g_{i} \end{matrix} \right)=\sum_{j=1}^{N} \left( \begin{matrix} \begin{matrix} J_{ij}x_{j} \\ G_{ij}x_{j} \end{matrix} \end{matrix} \right)\sim\mathcal{N}\left( 0,\Sigma\right)$ | (18) |
| --- | --- | --- |

The correlation matrix can be derived and shown to be diagonal:

|  | $\Sigma_{hh}=\mathbb{E}_{i}\left[ \sum_{j} J_{ij}x_{j}\sum_{k} J_{ik}x_{k} \right]\neq0$  $\Sigma_{\mathrm{hg}}=\mathbb{E}_{i}\left[ \sum_{j} J_{\mathrm{ij}}x_{j}\sum_{k} G_{\mathrm{ik}}x_{k} \right]=0$ | (19) |
| --- | --- | --- |

where the last equality follows from the independent, zero mean, nature of elements of **G** and **J**.

Now, since $h_{i}$ and $g_{i}$ are jointly normally distributed random variables with a diagonal covariance matrix, they are also independent. We then pass these representations through a nonlinearity. Using the fact that functions of independent variables are independent, the resulting output representations are also independent and hence uncorrelated.

This demonstration can be extended to many cases of direct interest. If we let $h$ and $g$ depend on different odors they are still independent and uncorrelated by a simple generalization of the previous argument:

|  | $h_{i}=\sum_{j} J_{ij}x_{1,j}$ and $g_{i}=\sum_{j} G_{ij}x_{2,j}$. | (20) |
| --- | --- | --- |

The experiments consider another similar case. Interpret h (g) as the unthresholded representation in the ipsilateral (contralateral) AON to an odor presented ipsilaterally (contralaterally). If the cross cortical matrix is random then thresholding these responses and mapping them cross cortically will not change their independence from one and other.

Previous work has shown that, if two correlated representations are projected through a random matrix, the resulting representations remain correlated (Babadi and Sompolinsky, 2014; Schaffer et al., 2018). The key difference in our work is that we are considering two different random matrices, one in each hemisphere. Hence, this derivation has shown that projecting a pair of correlated representations through two different random matrices eliminates the correlations between resulting outputs.

Therefore, since observed correlations between odors presented ipsilaterally and contralaterally are not zero, the cross cortical connectivity must be structured in some way.
